# Long-Term Lactulose Administration Improves Dysbiosis Induced by Antibiotic and *C. difficile* in the PathoGut^TM^ SHIME Model

**DOI:** 10.3390/antibiotics11111464

**Published:** 2022-10-24

**Authors:** Marta Calatayud, Cindy Duysburgh, Pieter Van den Abbeele, Dennis Franckenstein, Angelika Kuchina-Koch, Massimo Marzorati

**Affiliations:** 1 ProDigest BV, Technologiepark 82, 9052 Ghent, Belgium; 2 Center of Microbial Ecology and Technology (CMET), Ghent University, Coupure Links 653, 9000 Ghent, Belgium; 3 Fresenius-Kabi Deutschland GmbH, Else-Kröner-Str. 1, 64352 Bad Homburg, Germany; 4 Fresenius-Kabi Austria GmbH, Estermannstrasse 17, 4020 Linz, Austria

**Keywords:** *Clostridioides difficile*, gut microbiota, mucosal simulator of the human intestinal microbial ecosystem, in vitro, antibiotic, lactulose, prebiotic

## Abstract

*Clostridioides difficile* infection (CDI) is the leading cause of antibiotic-associated diarrhea and an important nosocomial infection with different severity degrees. Disruption of the gut microbiota by broad-spectrum antibiotics creates a proper environment for *C. difficile* colonization, proliferation, and clinical disease onset. Restoration of the gut microbial ecosystem through prebiotic interventions can constitute an effective complementary treatment of CDI. Using an adapted simulator of the human gut microbial ecosystem, the PathoGut^TM^ SHIME, the effect of different long-term and repeated dose lactulose treatments was tested on *C. difficile* germination and growth in antibiotic-induced dysbiotic gut microbiota environments. The results showed that lactulose reduced the growth of viable *C. difficile* cells following clindamycin treatment, shifted the antibiotic-induced dysbiotic microbial community, and stimulated the production of health-promoting metabolites (especially butyrate). Recovery of the gut microenvironment by long-term lactulose administration following CDI was also linked to lactate production, decrease in pH and modulation of bile salt metabolism. At a structural level, lactulose showed a significant bifidogenic potential and restored key commensal members of the gut ecosystem such as *Lactobacillaceae*, *Veillonellaceae* and *Lachnospiraceae*. These results support further human intervention studies aiming to validate the in vitro beneficial effects of lactulose on gut microbiome recovery during antibiotic exposure and CDI.

## 1. Introduction

*Clostridioides difficile* is a spore-forming, anaerobic pathogen, associated with healthcare-associated infections accompanied by substantial morbidity and mortality among individuals of different ages. It is considered to impact public health worldwide [1]. Based on a systematic review in 2015, it was estimated that the rate of overall health care facility-associated *C. difficile* infections (CDI) was at 3.54 per 10,000 patient-days per year increasing up to 11.08 in intensive care units [1]. Clinical illness can present different degrees of severity, ranging from mild diarrhea to severe colitis and death [2]. Some research has shown more diverse acquisition sources than previously thought, with shared occurrence of ribotypes between healthcare settings and the community, increasing the evidence of transmission in the intra-hospital environment and outside the hospital [3]. 

Due to strict anaerobic conditions required for the vegetative forms’ growth, spores are the main source of transmission and infection. Sporulation and further germination are essential for CDI establishment [4,5], whereas production of toxins is required to initiate symptomatology. Mechanisms and factors controlling *C. difficile* sporulation are not fully understood, but under certain stress conditions, the pathogen produces aerotolerant dormant spores to survive extreme environments [5,6]. 

A resilient gut microbial ecosystem confers resistance to CDI. However, antibiotic-treated patients, persons with non-antibiotic dysbiosis, immunocompromised or elderly individuals have a higher risk of developing CDI [7,8], especially during hospitalization course. The microbial metabolic repertoire can affect both, dormant spores and vegetative *C. difficile* cells. Moreover, primary bile acids taurocholic (TCA) and cholic acid (CA) have been described as bacterial metabolites directly linked to germination [9,10]. On the other hand, secondary bile acids and competition with commensal microbiota can hamper *C. difficile* outgrowth and colonization [10,11]. 

The massive use of antibiotics due to the coronavirus disease 2019 (COVID-19) pandemic could cause an increase in CDI, particularly in the elderly population [12]. It has been estimated that more than 70% of COVID-19 patients were treated with broad-spectrum antibiotics, such as clindamycin [13,14] or vancomycin [15] among others, to prevent bacterial co-infections [16,17]. Gut imbalance due to antibiotics [18] or severe acute respiratory syndrome coronavirus 2 (SARS-CoV-2) infection [19] might facilitate the occurrence of CDI, particularly in asymptomatic *C. difficile* carriers [12,20].

At present, the standard treatment for CDI includes antibiotic intake, with the most commonly used antibiotics being vancomycin, metronidazole, and more recently fidaxomicin, the latter showing lower antimicrobial resistance [21,22] and recurrence rates (15%) [23]. The risk of CDI recurrence is high, ranging from 20% after an initial episode to 60% after multiple prior recurrences, and it is potentially linked to the persistence of *C. difficile* spores in the gut and a defective immune response [23]. Other therapeutic options have also been described as promising tools for recurrence prevention, such as bezlotoxumab, a human monoclonal antibody blocking *C. difficile* toxin B [24,25] or fecal microbiota transplantation to restore gut ecosystem resilience to infection [26]. Despite effectiveness, the routine use of these therapies is hindered by high cost or lack of approved procedures and regulatory standards [26]. Therefore, the need for complementary strategies to prevent CDI and recurrence are still needed. In that sense, modulation of the gut microbial ecosystem through prebiotic, probiotic or synbiotic administration, especially in antibiotic-induced dysbiosis can improve intestinal homeostasis and confer prevention to infection or recurrence.

Prebiotics, substrates that are selectively utilized by host microorganisms conferring a health benefit [27], are well recognized modulators of gut microbiota activity and structure. Multiple mechanisms linking prebiotic compounds and health effects have been described, most of them intermingled between modulation of health-related bacteria, bacteria-derived metabolites and mucosal or systemic responses at the host level [28,29,30]. The most commonly recognized prebiotics are lactulose, inulin, fructo-oligosaccharides, galacto-oligosaccharides and human milk oligosaccharides [30]. Lactulose is a synthetic derivative of lactose, composed of β-1,4-glycosidic bounded galactose and fructose, which is resistant to digestion and absorption during small intestinal transit [31]. Upon entering in the colonic environment, lactulose exerts strong prebiotic properties by stimulating health-promoting bacterial groups (e.g., *Bifidobacterium* and *Lactobacillus* species) as well as inhibiting pathogenic colonization [32,33]. In addition, recent in vitro [34] and in vivo [35] studies have shown its potential in limiting detrimental effects of antibiotic therapy on the gut microbiome by inducting faster restoration of intestinal homeostasis, thereby enhancing the abundance of saccharolytic bacteria such as *Lactobacillus*, *Anaerostipes* and *Roseburia*. Furthermore, lactulose has been proven effective in reducing *C. difficile*-related diarrhea among patients receiving antibiotics [36], whereas other prebiotic compounds such as fructo-oligosaccharides and mannose have been shown to inhibit *C. difficile* adhesion and biofilm formation in an in vitro model of the gut epithelium [37]. In this study, we evaluated the effect of different long-term dosage regimens of lactulose on the instauration of CDI and *C. difficile* spore germination and recurrence using the in vitro PathoGut^TM^ Simulator of the Human Intestinal Microbial Ecosystem (SHIME) model mimicking antibiotic-induced gut dysbiosis. As the proximal colon (PC) is the main colonic area of fermentation of lactulose [38], effects of repeated lactulose dosing on microbial metabolic activity and structure were specifically assessed in the PC area. However, CDI typically occurs in the distal colon (DC) region, as *C. difficile* is not able to germinate under acidic conditions [39]. Furthermore, as secondary bile acids (mainly produced in the DC) can impact *C. difficile* sporulation and germination, *C. difficile* colonization and bile acid metabolism in the presence or absence of lactulose supplementation were specifically investigated in the in vitro DC, thereby assessing the effect of lactulose and/or produced metabolites of colonic lactulose fermentation on CDI onset and recurrence.

## 2. Results

### 2.1. Quality Control Measures for PathoGut^TM^ SHIME Stability Assessment

During the 12 weeks of the experiment, the total count of bacteria was evaluated by flow cytometry at different time points. In contrast to the antibiotic-treated reactors, where bacterial counts decreased during antibiotic exposure, lactulose supplementation increased bacterial counts, but changes were not statistically significant for any test condition (Supplementary Figure S1). Furthermore, the blank control (CTRL) arm showed expected stable values of short-chain fatty acids (SCFA) and branched-chain fatty acids (BCFA) during 12 weeks of the experiment, whereas any antibiotic treatment resulted in significant drops in bacterial metabolic activity during the clindamycin treatment (CLI) period or the vancomycin treatment (VNC) period, especially in the PC (Supplementary Figure S2). These results are indicative for the validity of the test system. 

### 2.2. Effect of Lactulose on the Establishment of CDI in the PathoGut™ SHIME

After the addition of *C. difficile* spores to the PC reactors during the CLI period, reproducible viable and spore counts between 4.83 and 4.78 colony forming units (CFU)/mL log units were obtained (Supplementary Figure S3).

**Figure 1:**
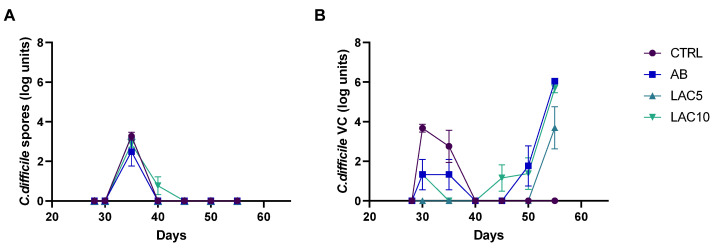
*C. difficile* spore counts (**A**) and viable cell (VC) counts (**B**) quantified by plating during the course of the control period (C; d28), the clindamycin treatment period (CLI; d30) and the CDI stabilization period (d35–55) of the PathoGut™ SHIME experiment in the distal colon reactors for four of the experimental arms, including arm 1 (CTRL), arm 2 (AB), arm 3 (LAC5) and arm 4 (LAC10). Dots represent mean ± SEM (*n* = 3) at different time points of the experiment. Two-way ANOVA was used to calculate statistically significant differences between the experimental test arms, with significance highlighted in the text (*p* < 0.05).

At the start of the CDI stabilization period (d35), *C. difficile* spores could be detected in the DC for all test conditions, though quickly decreased below the limit of detection in any of the conditions (Figure 1A). Only in the arm receiving lactulose treatment with 10 g/d during the CLI and CDI stabilization period (LAC10), *C. difficile* spores were still detectable at day 40, reaching significance compared to the other test conditions (*p* = 0.0122).

*C. difficile* viable cells were not detected in the CTRL arm during the experiment. In the antibiotic-treated control (AB) arm, on the other hand, viable cells gradually increased, reaching the maximum values obtained for all the test conditions at the end of the CDI stabilization period (6.7 ± 0.02 CFU log units). Lactulose treatment with 5 g/day during the CLI and CDI stabilization period (LAC5) significantly reduced the number of viable *C. difficile* cells (3.69 ± 1.85 CFU log units) compared to the AB (6.04 ± 0.12 CFU log units; *p* = 0.0031) or LAC10 (5.68 ± 0.37 CFU log units; *p* = 0.0158) arm on day 56, corresponding to the end of the CDI stabilization period (Figure 1B).

### 2.3. Effect of Lactulose on the Recurrence of CDI in the PathoGut™ SHIME

**Figure 2:**
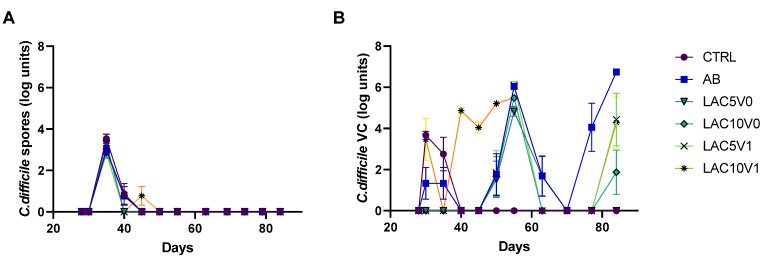
*C. difficile* spore counts (**A**) and viable cell (VC) counts (**B**) quantified by plating during the course of the control period (C; d28), the clindamycin treatment period (CLI; d30), the CDI stabilization period (d35–55), the vancomycin treatment period (VNC; d63) and the post-intervention period (PI; d70–84) of the PathoGut™ SHIME experiment in the distal colon reactors for six of the experimental arms, including arm 1 (CTRL), arm 2 (AB), arm 5 (LAC5V0), arm 6 (LAC10V0), arm 7 (LAC5V1) and arm 8 (LAC10V1). Dots represent mean ± SEM (*n* = 3) at different time points of the experiment. Two-way ANOVA was used to calculate statistically significant differences between the experimental test arms, with significance highlighted in the text (*p* < 0.05).

In arms 5 (receiving lactulose treatment with 5 g/d during the VNC and post-intervention (PI) period; LAC5V0), 6 (receiving lactulose treatment with 10 g/d during the VNC and PI period; LAC10V0), 7 (receiving lactulose treatment with 5 g/d during the VNC and PI period with lactulose supplementation initiated one day before starting vancomycin treatment; LAC5V1) and 8 (receiving lactulose treatment with 10 g/d during the VNC and PI period with lactulose supplementation initiated one day before starting vancomycin treatment; LAC10V1) of the PathoGut™ SHIME experiment, recurrence of *C. difficile* was evaluated after one week of VNC, combined with different lactulose dosage regimens. Following VNC, *C. difficile* spore counts remained below the limit of detection during the complete PI period (Figure 2A). Furthermore, *C. difficile* viable counts remained undetectable in any of the investigated arms of the PathoGut™ SHIME experiment until the end of the first week of the PI period (d70) (Figure 2B). However, following d70, the AB arm showed a gradual increase in *C. difficile* viable cell counts until the end of the investigation period (6.7 ± 0.02 CFU log units; d84). LAC5V0had a significant impact on *C. difficile* behavior (*p* < 0.001), as observed by the absence of viable cells during the complete PI period (d70-d84) (Figure 2B). In the LAC10V0 arm, viable cells were detected at the end of the PI period (d84), though at lower levels (1.87 ± 0.5 CFU log units) compared to the AB arm, reaching significance (*p* < 0.0001) (Figure 2B). When lactulose was administered 1 day before starting VNC (LAC5V1 and LAC10V1, respectively), the recurrence of viable cells was delayed compared to the AB arm and lower levels were detected at the end of the PI period (4.28–4.43 CFU log units), reaching significance for both test conditions compared with the AB arm (*p* = 0.0089 for LAC5V1, and *p* = 0.0042 for LAC10V1) (Figure 2B).

### 2.4. Effect of Long-Term Lactulose Intervention on Metabolic Activity after Clindamycin Application and CDI in the PathoGut™ SHIME

The PathoGut™ SHIME LAC5 and LAC10 arms were challenged with *C. difficile*, clindamycin and different lactulose dosage regimens during the CLI period, and subsequently stabilized during the CDI stabilization period upon further supplementation of lactulose. This section therefore focuses on the effect of lactulose fermentation on clindamycin-dysbiotic reactors. In general, microbial metabolic activity and structure response showed similar trends in the PC and DC. However, as the PC is the main area of fermentation of lactulose, results focus on this specific colonic region, with data of the DC only presented in case of specific biological significance (e.g., bile acid metabolism). 

#### 2.4.1. Markers of Microbial Activity

**Figure 3:**
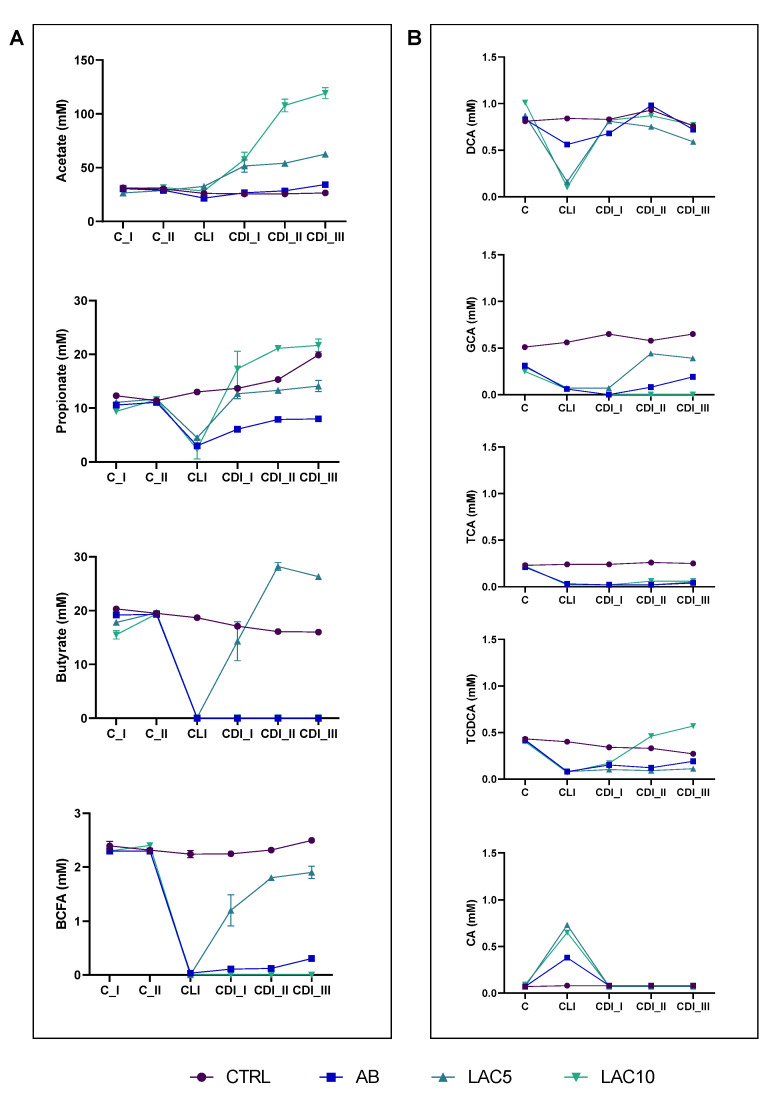
(**A**) Acetate, propionate, butyrate and branched-chain fatty acid (BCFA) levels (mM) in the proximal colon reactors and (**B**) taurocholic acid (TCA), taurochenodeoxycholic acid (TCDCA), glycocholic acid (GCA), deoxycholic acid (DCA) and cholic acid (CA) in the distal colon reactors during the course of the control period (C_I-II), the clindamycin treatment period (CLI) and the CDI stabilization period (CDI_I-III) of the PathoGut™ SHIME experiment for four of the experimental arms, including arm 1 (CTRL), arm 2 (AB), arm 3 (LAC5) and arm 4 (LAC10). Dots represent mean ± SEM (*n* = 3) for acetate, propionate, butyrate and BCFA, whereas *n* = 1 for TCA, TCDCA, GCA, DCA and CA. Two-way ANOVA was used to calculate statistically significant differences between the experimental test arms, with significance highlighted in the text (*p* < 0.05).

Acetate and propionate were increased in the lactulose supplemented arms LAC5 (acetate = 51.5–62.6 mM, propionate = 12.7–14.1 mM) and LAC10 (acetate = 57.6–119.2 mM, propionate = 17.3–21.7 mM) during the CLI period compared to the AB arm (acetate = 26.7–34.3 mM, propionate = 6.1–8.0 mM) and this until the end of the CDI stabilization period (Figure 3A), thereby reaching significance (*p* < 0.0001 for acetate and propionate at the third week of the CDI stabilization period (CDI_III) when comparing LAC5 and LAC10 with AB). Butyrate, on the other hand, only recovered in the LAC5 arm during the CDI stabilization period, reaching levels between 14.3 mM and 26.3 mM, compared to the AB arm and the LAC 10 arm, respectively (*p* < 0.0001) (Figure 3A). Regarding markers of proteolytic fermentation, LAC5 increased BCFA levels after antibiotic exposure (2.2–2.5 mM; *p* < 0.0001). In contrast, the AB and LAC10 arms showed BCFA levels close to zero after CLI and until the end of the CDI stabilization period (Figure 3A). Furthermore, lower ammonium values were quantified in LAC5 (112.6–156.0 mg/L) and LAC10 (45.4–83.1 mg/L) reactors compared to the AB (165.1–211.8 mg/L) and CTRL (320.2–340.7 mg/L) arm, respectively, reaching significance (*p* < 0.0001) (Supplementary Figure S4). 

When analyzing all time points together, LAC10 significantly increased acetate (*p* = 0.0460 vs. CTRL and *p* = 0.0410 vs. AB) and propionate (*p* = 0.0387 vs. AB) levels, whereas LAC5 had a significant effect on increasing butyrate (*p* < 0.0001 vs. AB) and BCFA (*p* < 0.0001 vs. CTRL and AB) levels compared with the control conditions (Supplementary Figure S5).

In the DC, a similar increase in acetate (*p* < 0.0001 vs. AB for LAC5 and LAC10) and propionate (*p* = 0.0002 for LAC5 vs. AB and *p* < 0.0001 for LAC 10 vs. AB) levels was observed during the CDI stabilization period following lactulose supplementation at both dosing rates tested. However, butyrate production was enhanced following lactulose administration, with LAC5 (17.7–32.6 mM; *p* < 0.0001) and LAC10 (12.7–13.6 mM; *p* > 0.9999) reaching similar and/or higher levels compared to the CTRL arm (11.2–12.7 mM), and significantly higher levels compared to the AB condition (5.6–6.4 mM; *p* < 0.0001 for LAC5 and *p* = 0.0004 for LAC10) (Supplementary Figure S6).

Finally, other general markers of microbial metabolic activity (base consumption and lactate) were recovered following CLI upon supplementing lactulose at either of the concentrations tested (LAC5 and LAC10), thereby reaching similar or even higher levels compared to the CTRL and AB arms, with significance being reached at the end of the CDI stabilization period for all test conditions (*p* < 0.0001) (Supplementary Figure S4). 

#### 2.4.2. Bile Acids Profile

The major bile acids in the DC reactor of the CTRL condition in the PathoGut^TM^ SHIME model were deoxycholic acid (DCA; 0.77 ± 0.06 mM) and glycocholic acid (GCA; 0.6 ± 0.06 mM). TCA and taurochenodeoxycholic acid (TCDCA) were found in lower amounts (0.23 ± 0.02 mM and 0.32 ± 0.05 mM, respectively), similarly to CA (0.08 ± 0.02 mM) (Figure 3B and Supplementary Figure S5). TCDCA showed a non-significant decrease within the course of the CLI and CDI stabilization periods, whereas non-significant increases in GCA were observed (Figure 3B, Supplementary Figure S5). CLI significantly reduced total bile acid content (*p* = 0.0372 in the AB arm) and only a slight recovery was observed for TCDCA and GCA at the end of CDI_III (Figure 3B). Co-administration of lactulose with clindamycin did not recover the bile acid profile, with LAC5 (1.4 ± 0.11 mM) and LAC10 (1.3 ± 0.06 mM) reactors having overall significantly lower values of total bile acids compared to the CTRL arm (2.1 ± 0.08 mM; *p* = 0.0375 for LAC5 and *p* = 0.0449 for LAC10), especially related to TCA (*p* = 0.0255 for LAC5 and *p* = 0.0307 for LAC10), GCA (*p* = 0.0542 for LAC5 and *p* = 0.0065 for LAC10) and TCDCA (*p* = 0.0591 for LAC5 and *p* = 0.9975 for LAC10) (Figure 3B and Supplementary Figure S5). However, LAC10 showed recovery of TCDCA levels, reaching similar values (0.32 ± 0.12 mM) than CTRL (0.29 ± 0.02 mM) when averaging over the CLI and CDI stabilization period (Supplementary Figure S5).

### 2.5. Effect of Long-Term Lactulose Intervention on Metabolic Activity after Vancomycin Treatment in the PathoGut™ SHIME

The PathoGut™ SHIME arms 5 to 8 were exposed to *C. difficile* spores and clindamycin during the CLI period, subsequently stabilized during the CDI stabilization period without any test product addition, and further challenged with vancomycin and different lactulose dosage regimens during VNC and PI periods. This section focuses on the effect of lactulose on vancomycin-dysbiotic reactors, however representative data from CLI and CDI periods are presented in the graphs to have an overall view of the time-course evolution during the experiment. As mentioned before, results focus on the PC region, with data of the DC only presented in case of significance (e.g., bile acid metabolism).

#### 2.5.1. Markers of Microbial Activity

**Figure 4:**
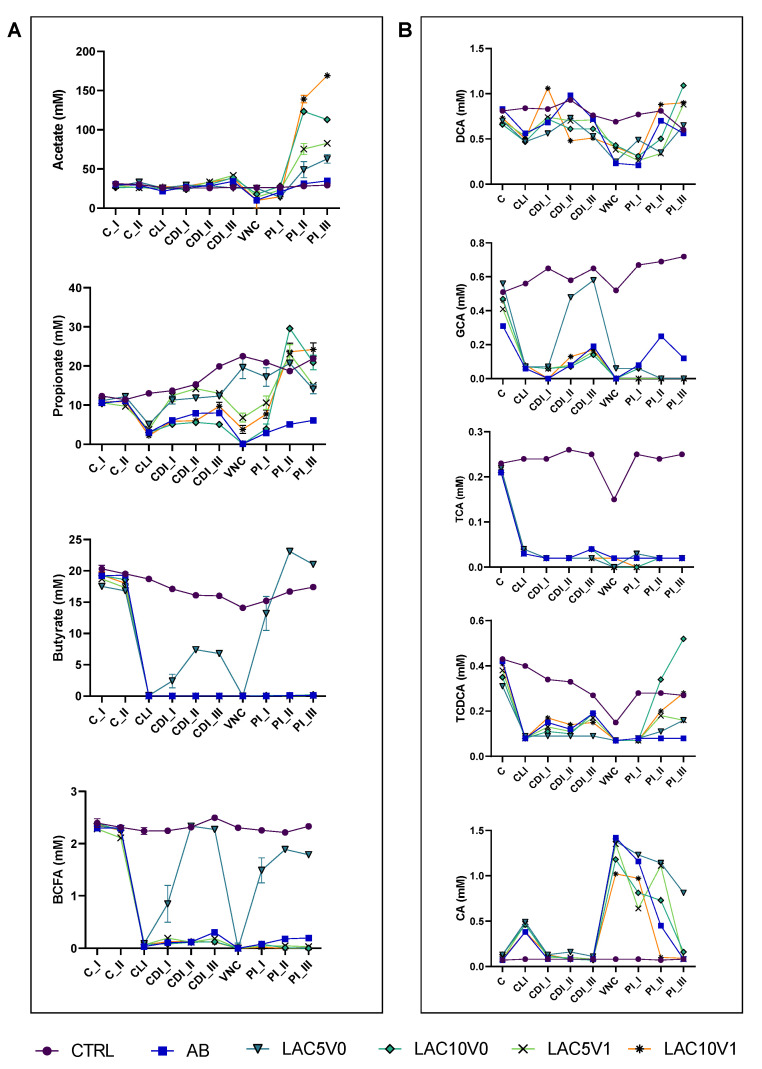
(**A**) Acetate, propionate, butyrate and branched-chain fatty acid (BCFA) levels (mM) in the proximal colon reactors and (**B**) taurocholic acid (TCA), taurochenodeoxycholic acid (TCDCA), glycocholic acid (GCA), deoxycholic acid (DCA) and cholic acid (CA) in the distal colon reactors during the course of the control period (C_I-II), the clindamycin treatment period (CLI), the CDI stabilization period (CDI_I-III), the vancomycin treatment period (VNC) and the post-intervention period (PI_I-III) of the PathoGut™ SHIME experiment for six of the experimental arms, including arm 1 (CTRL), arm 2 (AB), arm 5 (LAC5V0), arm 6 (LAC10V0), arm 7 (LAC5V1) and arm 8 (LAC10V1). Dots represent mean ± SEM (*n* = 3) for acetate, propionate, butyrate and BCFA, whereas *n* = 1 for TCA, TCDCA, GCA, DCA and CA. Two-way ANOVA was used to calculate statistically significant differences between the experimental test arms, with significance highlighted in the text (*p* < 0.05).

In LAC5V0 and LAC10V0 arms, a recovery of microbial activity was observed, especially at the highest dose of lactulose tested, when compared to the CTRL or AB arm (Figure 4). Concretely, higher levels of acetate (i.e., 14–123.4 mM) were observed during the course of the PI period for both lactulose doses compared with the control conditions, reaching significance (*p* < 0.0001) (Figure 4A). Only LAC5V0 was able to recover butyrate production (13.2–23.1 mM) above control levels (<LOQ) following antibiotic treatment, reaching significance compared to the other test conditions (*p* < 0.0001) (Figure 4A). Propionate levels were significantly increased by all lactulose dosing regimens (3.9–29.6 mM) compared to the AB arm (*p* < 0.0001), with highest values observed for LAC10V0 during the second week of the PI period (PI_II) and for LAC10V1 during the third week of the PI period (PI_III) (Figure 4A). 

When considering all timepoints, the only significant impact on SCFA production was observed following LAC5V0, increasing butyrate and propionate levels compared to the AB arm (*p* = 0.0266 and *p* = 0.0003 for propionate and butyrate, respectively) (Supplementary Figure S7). 

With regard to proteolytic fermentation markers, only the LAC5V0 arm showed a recovery of BCFA levels above concentrations obtained during the control (C) period, which was not observed for the other test conditions (Figure 4A, Supplementary Figure S8). Ammonium levels were significantly reduced following antibiotic treatment (144.8–159.1 mg/L) and were maintained below the levels for the CTRL condition (325.5–331.6 mM) (*p* < 0.0001). Treatment with lactulose at the highest dose tested, i.e., in the LAC10V0 and LAC10V1 arms of the PathoGut™ SHIME experiment, lowered ammonium levels even further towards the end of the PI period (29.5–79.5 mg/L), reaching significance compared to the AB arm (*p* < 0.0001 for both LAC10V0 and LAC10V1) (Supplementary Figure S8).

Other general markers of microbial activity such as base consumption and lactate production were increased by lactulose treatments at different regimens, reaching significance for most test conditions at the end of the PI period (*p* < 0.0001 for all test conditions compared with CTRL and AB with respect to base consumption, with respect to lactate production *p* < 0.0001 for LAC5V0, LAC10V0 and LAC10V1 vs. CTRL, *p* = 0.0278 for LAC5V1 vs. CTRL, *p* < 0.0001 for LAC10V0 and LAC10V1 vs. AB, *p* = 0.0004 for LAC5V0 vs. AB and *p* = 0.1290 for LAC5V1 vs. AB) (Supplementary Figure S8).

#### 2.5.2. Bile Acids Profile

Following VNC, overall bile acid metabolism was only partially recovered, except for LAC5V0, in which total bile acid production was similar to the CTRL (1.87 ± 0.33 mM). For specific bile acids, TCA values did not reach CTRL levels for any of the test conditions, reaching significance (*p* = 0.0002 for LAC5V0, LAC10V0 and LAC5V1, and *p* = 0.0003 for LAC10V1) (Figure 4B, Supplementary Figure S7). Furthermore, upon dosing 10 g lactulose per day (i.e., both LAC10V0 and LAC10V1), TCDCA (*p* = 0.5359 and *p* = 0.0398 for LAC10V0 and LAC10V1 vs. CTRL, respectively) and DCA (*p* = 0.2596 and *p* = 0.4480 for LAC10V0 and LAC10V1 vs. CTRL, respectively) levels raised to similar or even higher values compared with the CTRL arm at the end of the PI period (Figure 4B).

### 2.6. Dysbiosis Induction in the PathoGut^TM^ SHIME Model by Clindamycin and Vancomycin

Discriminant Analysis of Principal Components (DAPC) showed significant effects of clindamycin and vancomycin, both clustering separately from the CTRL condition (Supplementary Figure S9).

A linear discriminant analysis (LDA) Effect Size (LEfSe) analysis at the phylum level (Supplementary Figure S10) exhibited enrichment of Bacteroidota and Proteobacteria phylum in clindamycin- and vancomycin-treated reactors in the AB arm, respectively, whereas the CTRL condition was enriched in Firmicutes. Reduction of Firmicutes levels after antibiotic application in the AB arm was indeed confirmed when comparing the CLI period (in which clindamycin was dosed) with the C period, as well as when comparing the VNC period (in which vancomycin was dosed) and the CDI stabilization period (Supplementary Table S1). Compared to the CTRL condition, antibiotic treatment in the AB arm, especially vancomycin, reduced diversity indices Chao1 and Shannon, evenness and richness (Supplementary Figure S11). 

When analyzing microbial data at lower phylogenetic levels, a LefSe analysis identified the strongest impact on microbial communities for vancomycin. *Escherichia coli* (OTU9) and *Paraprevotella clara* (OTU12, OTU17) were enriched after CLI, whereas among other microbial members, vancomycin induced increases in *Klebsiella oxytoca* (OTU11), *Klebsiella pneumoniae* (OTU2) and *Citrobacter freundii* (OTU8) (Supplementary Table S2).

Notably, *Bifidobacteriaceae* OTU5 (related to *Bifidobacterium adolescentis*) and OTU14 (related to *Bifidobacterium longum*), as well as *Akkermansia muciniphila* in the DC were significantly reduced with both antibiotics (Supplementary Table S2).

### 2.7. Effect of Lactulose on Gut Dysbiosis Induced by Clindamycin and C. difficile

**Figure 5:**
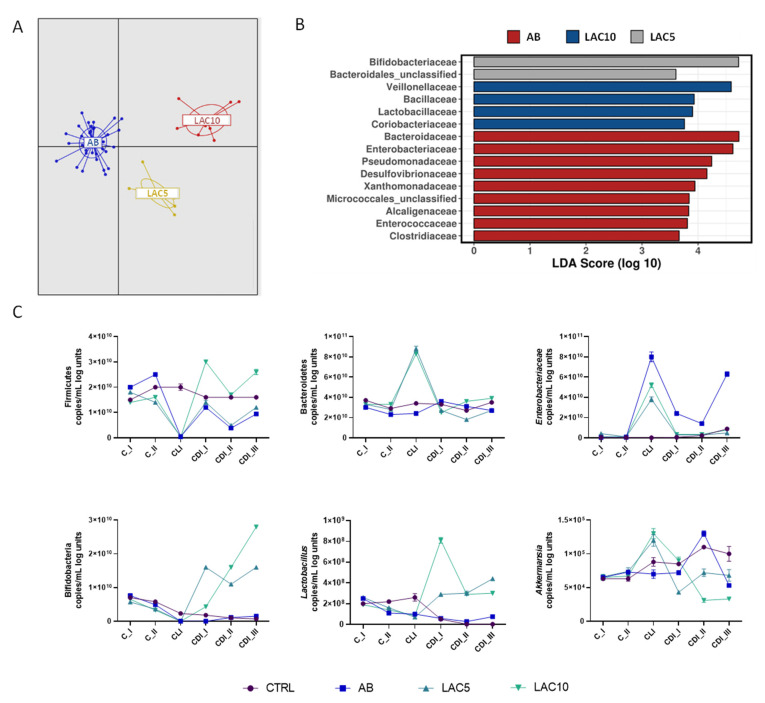
Effect of lactulose on clindamycin and *C. difficile*-induced dysbiosis in the proximal colon of the PathoGut^TM^ SHIME model. (**A**) Discriminant Analysis of Principal Components (DAPC) for three of the experimental arms of the PathoGut™ SHIME experiment, including arm 2 (AB), arm 3 (LAC5) and arm 4 (LAC10). (**B**) Linear discriminant analysis Effect Size (LEfSe) at family level for three of the experimental arms of the PathoGut™ SHIME experiment, including arm 2 (AB), arm 3 (LAC5) and arm 4 (LAC10). (**C**) qPCR counts (copies/mL log units) of Firmicutes, Bacteroidetes, *Enterobacteriaceae*, lactobacilli, bifidobacteria and *Akkermansia muciniphila* during the course of the control period (C_I-II), the clindamycin treatment period (CLI) and the CDI stabilization period (CDI_I-III) of the PathoGut™ SHIME experiment for four of the experimental arms, including arm 1 (CTRL), arm 2 (AB), arm 3 (LAC5) and arm 4 (LAC10). Dots represent mean ± SEM of technical replicas of qPCR of one time point per period. Two-way ANOVA was used to calculate statistically significant differences between the experimental test arms, with significance highlighted in the text (*p* < 0.05).

Overall, the effect of lactulose treatments on gut microbial community composition is given in Figure 5A. LAC5 and LAC10 arms clustered separately from the AB arm (Adonis test based on Bray–Curtis distance, *p* < 0.001). At family level, a LefSe analysis showed enhancement of *Bacteroidaceae*, *Enterobacteriaceae* and *Pseudomonaceae* during CLI. LAC5 resulted in enrichment of *Bifidobacteriaceae* and *Bacteroidales_unclasified*, whereas LAC10 enhanced *Veillonellaceae*, *Bacillaceae*, *Lactobacillaceae* and *Coriobacteriaceae* (Figure 5B). In the PC, LAC10 induced a reduction of *Bacteroidaceae* and, in the DC, an increase in *Eubacteriaceae* (Supplementary Table S3). At lower phylogenetic level, lactulose at both doses (LAC5 and LAC10) increased *Bifidobacteriaceae* OTU5 (related to *Bifidobacterium adolescentis*) and *Veillonellaceae* OTU7 (related to *Veillonella dispar*), which remained below the limit of detection in the AB arm following CLI and until the end of the 3-week CDI stabilization period in the PC (Supplementary Table S4). *Faecalibacterium prausnitzii*, on the other hand, was stimulated following lactulose supplementation, with strongest effects observed for the highest dose tested (10 g/d) during CLI (Supplementary Table S5). In addition, prolonged lactulose supplementation inhibited *Clostridiaceae* outgrowth compared to AB reactors, where *Clostridiaceae* increases were mainly caused by OTU99 (*Clostridium tertium*) in the DC (Supplementary Figure S12). 

qPCR results showed that Firmicutes levels, significantly reduced by CLI (*p* < 0.0001), were recovered by LAC10 treatment during the CDI stabilization period, whereas LAC5 showed the same trend as the AB condition (Figure 5C). Bacteroidetes levels were significantly higher in the LAC5 and LAC10 arms during the CLI period (*p* < 0.0001 vs. CTRL and AB, respectively). This effect was not observed for the CTRL or AB arms. *Enterobacteriaceae* were strongly increased during antibiotic exposure in all reactors. Lower *Enterobacteriaceae* levels were observed for LAC5 and LAC10 during the CLI period compared to the AB condition (*p* < 0.0001 for both test conditions). A recovery to levels obtained during the C period was observed during the CDI stabilization period following prolonged lactulose supplementation at both doses tested (*p* = 0.1384 and *p* = 0.9347 vs. CTRL for LAC5 and LAC10, respectively), but not in AB reactors which showed higher *Enterobacteriaceae* levels especially during CDI_III (*p* < 0.0001 vs. CTRL) (Figure 5C). qPCR data confirmed the stimulatory effect of both lactulose doses on *Bifidobacterium* spp. and *Lactobacillus* spp., with the strongest effect observed for LAC10 (Figure 5C). LAC5 and LAC10 raised *Bifidobacterium* spp. and *Lactobacillus* spp. above levels seen in the CTRL and AB arms (*p* < 0.0001 for all test conditions). During the CLI period, *Akkermansia muciniphila* was maintained at a higher level than in the CTRL and AB arms for both lactulose-supplemented reactors (LAC5 and LAC10; *p* = 0.0003 for LAC5 vs. CTRL and *p* < 0.0001 for LAC10 vs. CTRL and LAC5/LAC10 vs. AB). However, at the end of the CDI stabilization period, *Akkermansia muciniphila* decreased to similar (LAC5; *p* = 0.1728) or lower (LAC10; *p* = 0.0376) levels than in the AB arm. 

Similar trends were observed in the DC (Supplementary Figure S13), with some taxon-specific differences. *Enterobacteriaceae* levels were not reduced by lactulose treatments during CLI period and *Akkermansia muciniphila* was significantly supported by LAC5 during the first (CDI_I) and second (CDI_II) week of the CDI stabilization period, reaching control levels at the end of CDI_III (*p* = 0.0572 for LAC5 vs. CTRL at CDI_III).

### 2.8. Effect of Lactulose on Gut Dysbiosis Induced by Vancomycin and C. difficile

**Figure 6:**
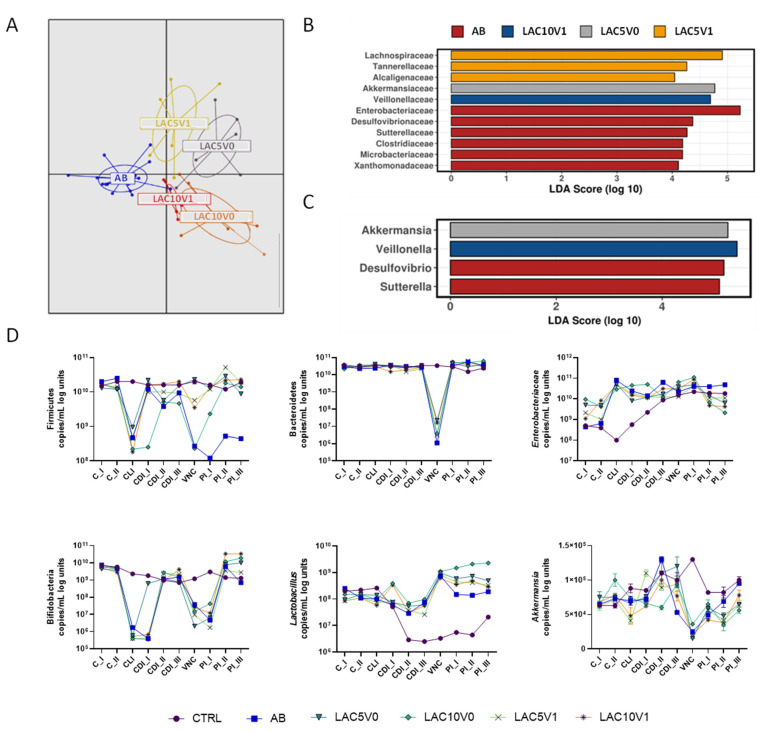
Effect of lactulose on vancomycin and *C. difficile*-induced dysbiosis in the proximal colon of the PathoGut^TM^ SHIME model. (**A**) Discriminant Analysis of Principal Components (DAPC) for five of the experimental arms of the PathoGut™ SHIME experiment, including arm 2 (AB), arm 5 (LAC5V0), arm 6 (LAC10V0), arm 7 (LAC5V1) and arm 8 (LAC10V1) following vancomycin treatment. (B + C) Linear discriminant analysis Effect Size (LEfSe) at family (**B**) and genus (**C**) level for five of the experimental arms of the PathoGut™ SHIME experiment, including arm 2 (AB), arm 5 (LAC5V0), arm 6 (LAC10V0), arm 7 (LAC5V1) and arm 8 (LAC10V1) following vancomycin treatment. (**D**) qPCR counts (copies/mL log units) of Firmicutes, Bacteroidetes, *Enterobacteriaceae*, lactobacilli, bifidobacteria and *Akkermansia muciniphila* during the course of the control period (C_I-II), the clindamycin treatment period (CLI), the CDI stabilization period (CDI_I-III), the vancomycin treatment period (VNC) and the post-intervention period (PI_I-III) of the PathoGut™ SHIME experiment for six of the experimental arms, including arm 1 (CTRL), arm 2 (AB), arm 5 (LAC5V0), arm 6 (LAC10V0), arm 7 (LAC5V1) and arm 8 (LAC10V1). Dots represent mean ± SEM of technical replicas of qPCR of one time point per period. Two-way ANOVA was used to calculate statistically significant differences between the experimental test arms, with significance highlighted in the text (*p* < 0.05).

Overall, both lactulose supplementation dosages (LAC5V0, LAC10V0, LAC5V1 and LAC10V1 arms) had a significant effect on the gut microbial community, compared to vancomycin alone (AB arm) (*p* < 0.001, Adonis test based on Bray–Curtis distance, Figure 6A). AB reactors encounted high numbers of *Enterobacteriacea*, *Desulfovibrionaceae*, *Sutterellaceae*, *Clostridiaceae*, *Microbacteriaceae* and *Xanthomonadaceae* (Figure 6B), whereas LAC5V1 induced an enrichment of *Lachnospiraceae*, *Tannerellaceae* and *Alcaligenaceae* (Figure 6B). 

Similar to the effect of lactulose in clindamycin-induced dysbiosis, lactulose administration, especially at higher doses, recovered *Eubacteriaceae* abundance in the DC (for LAC10V0 and LAC10V1) and decreased *Bacteroidaceae* levels in the PC (for LAC10V1) (Supplementary Table S6–S9). Moreover, LAC10V1 also enriched *Veillonellaceae* family (Figure 6B) and *Bifidobacteriaceae* (Supplementary Figure S14). At genus level, *Akkermansia* had a LDA score above 4 in the LAC5V0 arm, whereas *Veillonella* was increased in LAC10V1 reactors (Figure 6C). At lower phylogenetic level, a rise in *Lachnospiraceae* family was mainly related to lactulose-stimulatory effect on OTU1 (*Clostridium clostridioforme/bolteae*) in the PC and DC (Supplementary Tables S10 and S11). LAC5V0 augmented *Bacteroides salyersiae* (OTU66) and *Megasphaera* spp. (OTU28), whereas for LAC5V1 an enrichment in *Bacteriodes* spp., with augmentations of OTU20 (*Bacteroides salyersiae*), OTU25 (*Bacteroides finegoldii*) and OTU105 (*Bacteroides ovatus*), as well as OTU1 (*Clostridium clostridioforme/bolteae*) and OTU95 (*Bifidobacterium faecale*) could be detected (Supplementary Figure S15). At higher doses, LAC10V0 increased OTUs related to *Bifidobacterium longum* (OTU14), *Bifidobacterium longum subsp. suillum* (OTU38, OTU53) and *Lactiplantibacillus plantarum* (OTU41). LAC10V1 showed a higher effect on *Bifidobacterium adolescentis* (OTU5) and *Enterococcus faecalis* (OTU88) (Supplementary Figure S15).

Diversity indices Chao1 and Shannon, evenness and richness, were reduced by VNC (in the AB arm), compared with the CTRL condition, whereas lactulose co-administration (i.e., for LAC5V0, LAC10V0, LAC5V1 and LAC10V1) showed a slight tendency to increase different indices during the PI period (Supplementary Figure S16).

qPCR showed a significant reduction in Firmicutes, Bacteroidetes, bifidobacteria and *Akkermansia muciniphila* after VNC (*p* < 0.0001 AB vs. CTRL). All lactulose doses (i.e., for LAC5V0, LAC10V0, LAC5V1 and LAC10V1) recovered Firmicutes levels above AB levels and to similar levels than the CTRL condition, especially LAC5V0, LAC5V1 and LAC10V1 (Figure 6D). All reactors, including the AB arm, recovered Bacteroidetes levels during the PI period to levels close to the CTRL condition. *Enterobacteriaceae* showed different levels during the C period for the different test conditions, likely due to biological variation on PathoGut^TM^ SHIME colonization during the first time points of the experiment. In the PC (Figure 6D), *Enterobacteriaceae* levels showed steady increases in both the CTRL and AB arm during the course of the experiment, and despite lactulose reactors (i.e., LAC5V0, LAC10V0, LAC5V1 and LAC10V1) showed high levels of *Enterobacteriaceae* during the different periods, at the end of the experiment levels were lower than in the AB arm. In the DC (Supplementary Figure S17), the effect of VNC on *Enterobacteriaceae* was more evident, with a significant increase compared to the CTRL condition (*p* < 0.0001), and a trend to lower levels when lactulose was co-administered (*p* < 0.0001). Vancomycin-induced reduction in bifidobacteria levels detected by 16S rRNA sequencing were confirmed by qPCR. However, lactulose, especially at the highest doses, increased bifidobacteria copies above CTRL and AB levels (*p* < 0.0001) (Figure 6D). *Lactobacillus* spp. copies were significantly reduced during the course of the experiment in the CTRL arm, whereas maintained above control levels in all the other reactors. Compared with CDI_III, VNC increased *Lactobacillus* spp. levels but decreased later on during the PI period. All lactulose treatments showed higher *Lactobacillus* spp. levels than the AB arm during two weeks of the PI period (*p* < 0.0001), and the high levels were maintained until the end of the PI period in the LAC10V0 reactor. Finally, in the PC, *Akkermansia muciniphila* (Figure 6D) was significantly reduced by VNC compared to the CTRL condition (*p* < 0.0001) and any of the lactulose treatments was able to recover *Akkermansia muciniphila* levels (*p* = 0.0066 for LAC5V0, *p* = 0.0001 for LAC10V0, *p* = 0.0030 for LAC5V1 and *p* = 0.3347 for LAC10V1 compared to AB). In the DC, high levels of *Akkermansia muciniphila* were detected in LAC5V0 reactors during the PI period, reaching higher levels than the CTRL condition (*p* < 0.0001, whereas for other test conditions *p* = 0.1235 vs. CTRL) (Supplementary Figure S17). 

## 3. Discussion

CDI cause significant morbidity and mortality with limited alternative preventive options [1]. Considering the high recurrence and mortality rates associated with the disease, extensive research has been conducted to develop more specific treatments. However, common CDI antibiotic treatments result in gut dysbiosis by killing indigenous microbes that are important for preventing recurrence of CDI. Strategies to maintain or restore the intestinal microbiota, i.e., through the use of prebiotic intake to promote colonization resistance and to boost the immune system, have been proposed for disease prevention and treatment [40]. A strong modulatory capacity of lactulose on the gut microbiota, increasing *Bifidobacterium* and *Blautia* levels in mice has been demonstrated [41]. In a human intervention study, lactulose at low doses (3–5 g/d) showed significant increase in fecal bifidobacteria, acetic and lactic acid levels, and reduction of *Bacteriodaceae*, clostridia, pH, indole and branched fatty acids [32]. Mukherjee et al. have reported that beyond the bifidogenic effect, lactulose is also an immunomodulatory molecule in vitro, and when combined with the TLR-9 agonist CpG-ODN, enhances galectin-9, IFN-γ, and regulatory IL-10 concentrations in a co-culture of enterocytes and peripheral blood mononuclear cells [42]. The link between gut microbiota, lactulose, and immunomodulation has previously been established in animal studies [43,44], and it is one of the core benefits of prebiotic intake [45]. In addition, in vitro studies have shown that lactulose is not used by *C. difficile* as growth substrate [46], thereby showing potential as a candidate to maintain and/or restore intestinal homeostasis in response to CDI without promoting *C. difficile* proliferation. 

In this study, the effect of lactulose on colonic microbial ecology has been evaluated using the PathoGut^TM^ SHIME model representing the PC and DC. When a healthy microbial ecosystem was present in the reactors, vegetative forms and germination of *C. difficile* spores were gradually eliminated, as in vivo non-susceptible host microbiota colonization resistance can protect colonic niches from *C. difficile* engraftment [40]. Antibiotic-induced dysbiosis caused by clindamycin and vancomycin significantly affected microbial ecology, especially on butyrate-producing microorganisms, bifidobacteria, and bile acid metabolism.

Despite lactulose supplementation using different dosing strategies was not able to eliminate CDI, vegetative cell counts were reduced, CDI development was delayed, and butyrate levels were recovered in lactulose-vessels after CLI or VNC following a 5 g/d dosing regimen. Furthermore, GCA was higher with lactulose treatment at 5 g/d than in all the other conditions though not reaching control levels, whereas higher doses of lactulose (10 g/d) had a strong recovery effect on acetate, lactate, propionate, TCA, TCDCA and DCA levels. 

In humans, the main primary bile acids are CA and CDCA, whereas major conjugated forms are TCA and GCA. Once in the gastrointestinal tract, the gut microbiota metabolizes primary bile acids into a diverse array of secondary bile acids, mostly by deconjugation in proximal gut segments and epimerization/dehydroxylation within the large intestine. Bile salt hydrolases have been described in several commensal gut microbiota members belonging to *Clostridium, Bacteroides, Lactobacillus, Bifidobacterium*, and *Enterococcus* genera. At the same time, specific 7α-dehydroxylation reactions leading to CA transformation to DCA are restricted to specific members of *Clostridium* spp. and *Eubacterium* spp. [47]. Specifically, CA was increased in the PathoGut^TM^ SHIME by antibiotic exposure, indicating the susceptibility of 7α-dehydroxylating-bacterial species. Studies in antibiotic-treated mice have shown a higher susceptibility to *C. difficile* colonization, linked to decreased secondary bile acids and an increase in primary bile acids [48]. Furthermore, TCA, GCA and CA were shown to promote *C. difficile* spore germination in vitro and in vivo (Reviewed in [11]). In this study, the fastest recovery of bile acid metabolism was observed for LAC10V1, likely due to the stimulatory activity of *Lachnospiraceae*, *Ruminococcaceae* and *Eubacteriaceae* families, which are also known to be involved in the formation of different isoforms of secondary bile acids [49]. LAC5V0 was the only test condition showing absence of CDI recurrence after three weeks PI, also increasing DCA concentrations during the first PI week (PI_I) in the DC. The potential link between a fast re-establishment of DCA concentrations and the prevention of CDI recurrence suggests potential “sensitive windows” for preventing CDI, a hypothesis requiring further confirmation.

CDI susceptibility has been associated with decreases in gut bacterial diversity and Proteobacteria increases in mouse models’ gut microbiota [11]. Human studies also showed increased lactobacilli and clostridia and reduced bifidobacteria and *Prevotella* in CDI patients [50]. We observed similar trends, with *Clostridiaceae* increases and strong reductions in bifidobacteria populations in both colon regions, confirmed by qPCR quantification. Previous research showed a positive effect of lactulose supplementation on antibiotic-induced dysbiosis, increasing acetate and lactate levels, and promoting *Bifidobacterium* spp. growth [34,38].

Hong et al. showed that microbial species considered as human pathogens, including *Klebsiella* spp., co-colonized with *C. difficile* in asymptomatic patients [51]. It was indeed observed that antibiotics, and especially vancomycin, enriched PathoGut^TM^ SHIME reactors in potential pathogenic microorganisms involved in antibiotic-associated diarrhea such as *Klebsiella pneumoniae*, though the current design did not allow to determine if stimulation of *Klebsiella* spp. was caused by antibiotic exposure or by the presence of *C. difficile*. Interestingly, lactulose supplementation helped to recover beneficial gut commensals such as *Bifidobacterium* spp., *Lactiplantibacillus plantarum* or *Enterocloster bolteae* in antibiotic-treated vessels infected with CDI, whereas at the same time reducing levels of *Klebsiella pneumoniae*. Hence, further studies are proposed to identify the potential interplay between prebiotics, CDI engraftment and recurrence, and other gut members, for example, through quorum sensing molecules.

Bifidobacteria are one of the key members of the human gut microbiota, with beneficial effects on human physiology. *Bifidobacterium* spp. produce lactate and acetate from carbohydrate fermentation, and have significative effects on maintaining intestinal homeostasis and communication with the host [52]. Acetate is an important byproduct in the synthesis of butyrate, which was increased in lactulose-treated arms compared to AB. This suggests a potential cross-feeding mechanism with butyrate-producing bacteria such as *Faecalibacterium prausnitzii* [53], which was also increased following lactulose supplementation at high dose (10 g/d) during CDI. Butyrate acts as a main energy source for colonocytes and is involved in maintaining the epithelial barrier. It acts as metabolic regulator and immunomodulatory molecule, similar to propionate, which has been linked with multiple beneficial effects on the host [54,55]. It could be shown in a mouse model, that butyrate was not able to prevent CDI colonization, but indirectly lowered the pathogenicity of colitis induced by *C. difficile* by reducing the intestinal inflammation and bacterial translocation [56]. 

Lactulose, especially at 10 g/d, also induced a significant increase in lactate levels in the PC, which is one of the sources for the production of downstream metabolites. Equilibrium between lactate production and conversion to propionate and butyrate has been shown to play a critical role in maintaining gut microbial stability, with microbial communities with low numbers of lactate-utilizing bacteria, mainly Bacteroidetes and Firmicutes, being less stable to perturbations [57]. Other bacterial molecules with potential bioactivity against CDI have also been described, such as valerate or indole [58,59]. However, these substances were not measured in the present study. As the full array of metabolites is present in the PathoGut^TM^, SHIME, a metabolomic approach is proposed in further studies to analyze other potential molecules involved in CDI colonization and recurrence resistance.

Links between different gut microbial members have also been described during CDI in humans. Kim et al. recently reported a negative correlation of *Bifidobacterium* and *Bacteroides* genus with the abundance of toxigenic *C. difficile* in the gut microbiome [60]. *Bifidobacterium* spp. and *Bacteroides* spp. were significantly enriched in the colonic reactors of the PathoGut^TM^ SHIME following repeated lactulose supplementation, suggesting that, despite the fact that CDI was not abolished by lactulose, long-term prebiotic treatment can recover and maintain eubiotic gut ecosystems and potentially increasing CDI resistance. Furthermore, long-term lactulose administration stimulated *Akkermansia muciniphila* in the DC, an observation previously described in in vitro [38] and in vivo studies [61]. *Akkermansia muciniphila* is a mucin-degrader gut commensal and promising candidate as next-generation probiotic due to beneficial effects on intestinal barrier, mucus production, reduction of intestinal inflammation, and homeostatic effect on host metabolism [62].

Human data support the beneficial effects of lactulose administration in CDI patients. In hospitalized patients with severe cirrhosis, lactulose use was associated with a lower CDI risk [63]. Recently, it has been reported that repeated doses of lactulose during antibiotic therapy reduced CDI during hospitalization (2.3%) compared with the antibiotic-group (9.7%) [36]. Overall, the effect of antibiotics and CDI in the PathoGut^TM^ SHIME model showed a similar trend as observed in the gut microbiota of CDI patients, whereas the well-known prebiotic potential of lactulose was reflected in metabolic activity and microbial structure in vitro. Despite the limitations of in vitro models, the continuous, long-term dosage of lactulose and the capacity to evaluate the evolution of gut microbial ecosystems in defined environments without significant confounders as diet supposes an advantage of the PathoGut^TM^ SHIME system. Authors acknowledge the limitation of donor sample, and further studies including more donors are proposed to evaluate interindividual differences on gut microbiota response to lactulose. 

## 4. Materials and Methods

### 4.1. Test Products

All chemicals were obtained from Merck (Darmstadt, Germany) unless stated otherwise. Lactulose was obtained from Fresenius Kabi iPSUM S.r.l. (Vicchio, Italy).

### 4.2. Fecal Sample

An adult donor was selected based on the following inclusion criteria: healthy, age between 25–35 years, no antibiotics or any other drug intake at least during the last six months. The fecal sample was collected according to the ethical approval of the University Hospital Ghent (reference number B670201836585). The fecal sample was obtained in a plastic container with an “Oxoid™ AnaeroGen™” bag (Oxoid, Basingstoke, Great Britain) to limit the sample’s exposure to oxygen and immediately used. 

### 4.3. Generation of Clostridioides difficile Spores

*C. difficile* spores were obtained from plate culture of the commercial strain *Clostridioides difficile* (ATCC^®^ BAA1382™, LGC standards, Wesel, Germany). After obtaining glycerol stocks following supplier instructions, *C. difficile* spores were produced as described by Edwards et al. [64] with some modifications. Briefly, an aliquot of *C. difficile* glycerol stock was plated on pre-reduced plates containing BD™ *Clostridium difficile* agar with 7% sheep blood (BD 254406, BD Diagnostic Systems, Heidelberg, Germany) and incubated for 24–48h at 37 °C under anaerobic conditions. Subsequently, a colony obtained from the agar plate was transferred to liquid reinforced clostridial medium (RCM) and incubated at 37 °C for 24 h under shaking and anaerobic conditions. To generate *C. difficile* spores, 100 µL of RCM *C. difficile* culture was transferred to CDC anaerobic agar plates supplemented with 5% sheep blood (L007357, BD Diagnostic Systems, Germany) and incubated anaerobically at 37 °C for 10 days. Thereafter, spores were recovered from plates in anaerobic phosphate buffer saline (PBS) containing 1 mL/L Tween 80 (Sigma-Aldrich, Overijse, Belgium) as a vehicle, followed by centrifugation at 4500× *g* for 15 min and suspension of the obtained pellet in 30 mL PBS + Tween80. This step was repeated three times for a complete wash of spores. All tubes were pooled, and 1 mL aliquots of the obtained spore stock were heated at 65 °C in a warm bath for 10 min to kill vegetative cells. Spore quantification was performed by mixing 100 µL of the spore stock with 100 µL absolute ethanol incubated at room temperature for 1 h, followed by seeding serial dilutions in PBS on BD™ *Clostridium difficile* agar with 7% sheep blood plates and incubation for 48h at 37 °C under anaerobic conditions. Spore stocks at minimum 10^7^ CFU/mL were stored at −20 °C and used for up to 3 months.

### 4.4. Long-Term PathoGut^TM^ SHIME

The reactor setup mimicked different regions of the human gastrointestinal tract and was adapted from the SHIME® (ProDigest and Ghent University, Belgium) as previously described by Molly et al. [65]. A succession of three double-jacketed reactors per test condition arm was established, with a first reactor (St + Si) simulating subsequently the stomach (St) and the small intestine (Si), and two more reactors simulating the PC and DC. PC and DC reactors were inoculated with the fecal sample from one healthy adult human donor (male, 32y) and maintained with a fixed volume of 500 and 800 mL and pH of 5.6–5.9 and 6.6–6.9 for PC and DC, respectively. Fecal inoculation was prepared with a mixture 1:5 (*w/v*) of freshly collected fecal sample in anaerobic phosphate buffer (K_2_HPO_4_ 8.8 g/L; KH_2_PO_4_ 6.8 g/L; sodium thioglycolate 0.1 g/L; sodium dithionite 0.015 g/L). After homogenization (10 min, BagMixer 400, Interscience, Louvain-LaNeuve, Belgium) and removal of big particles by centrifugation (2 min, 500*g*), 5% (*v/v*) of fecal inoculum was added to the colonic reactors. Reactors were constantly stirred and maintained at 37 °C. The feeding regime and retention times in each vessel were as described by Possemiers et al. [66]. 

**Figure 7:**
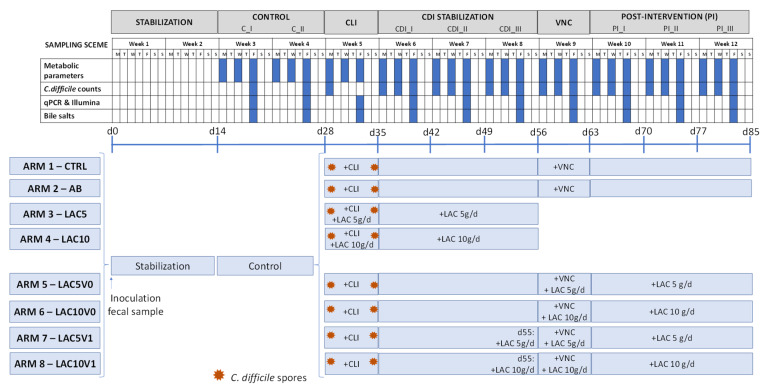
Schematic representation of the experimental set up for each of the eight arms of the PathoGut™ SHIME experiment, including the sampling scheme for each of the experimental periods, i.e., stabilization period (2 weeks), control period (C; 2 weeks), clindamycin treatment period (CLI; 1 week), *C. difficile* infection (CDI) stabilization period (3 weeks), vancomycin treatment period (VNC; 1 week) and post-intervention period (PI; 3 weeks). Antibiotic and test product additions were indicated for each of the experimental arms by “+”. CLI = clindamycin; FC = flow cytometry; LAC = lactulose; VNC = vancomycin.

In the current study, the eight arms of the PathoGut™ SHIME experiment were operated as described in Figure 7. The different experimental periods included stabilization (two weeks), C (C_I, C_II, corresponding to two consecutive weeks), CLI (one week), CDI stabilization (CDI_I, CDI_II, CDI_III, corresponding to three consecutive weeks), VNC (one week), and PI (PI_I, PI_II, PI_III, corresponding to three consecutive weeks). During the stabilization and C period (i.e., the four weeks following fecal inoculation), microbial communities in the different reactors were stabilized and subsequently, treatments were initiated. At the start and at the end of the CLI period, *C. difficile* spores were dosed in the PC at a concentration of 10^7^ CFU per reactor (4.6 log CFU/mL) in a single dose in all the arms.

Arm 1 was maintained during the full length of the experiment without any antibiotic and/or lactulose treatment, therefore representing the CTRL. The AB arm (arm 2) and arms 3 to 8 were treated with clindamycin at a daily dose of 33.9 ppm during the 7-day CLI period. Furthermore, arms 3 (LAC5) and 4 (LAC10) received lactulose supplementation at a low (5 g/day) and high (10 g/day) dosing rate, respectively, on top of the standard feed composition during the CLI (1 week) and following CDI stabilization (3 weeks) period.

After the CDI stabilization period, arms 2, 5, 6, 7 and 8 were dosed with vancomycin (125 ppm/day, 1 week) in the PC during the 7-day VNC period. In order to evaluate the potential effect of lactulose on recurrence of CDI, arms 5 (LAC5V0) and 6 (LAC10V0) were co-treated with the low and high dose of lactulose, respectively, during the VNC period. After vancomycin treatment, both arms were further treated with 5 g/day and 10 g/day of lactulose (LAC5V0 and LAC10V0, respectively) on top of the normal feed composition during the PI period (3 weeks). Similarly, arms 7 (LAC5V1) and 8 (LAC10V1) were supplemented with lactulose following the low and high dosing regime, respectively, during the VNC and PI period, though lactulose administration was initiated one day before starting VNC. 

At different time points, samples from the different colonic reactors were obtained for downstream analysis (Figure 7). In all arms, microbial metabolic activity was assessed by acid/base consumption, lactate, ammonium, SCFA, and BCFA production by collecting samples from each colonic reactor three times per week from the C period onwards. Bile salts quantification and microbial structure (qPCR of specific microbial taxa and 16S rRNA Illumina sequencing) were evaluated once per week from the C period onwards, whereas levels of *C. difficile* were evaluated twice during the C period, once at the start of the CLI period and three times per week from the CDI stabilization period onwards.

### 4.5. Analysis of C. difficile Spore Germination and CDI Establishment

The establishment of CDI and the potential effect of lactulose on *C. difficile* spore germination was monitored by culturing samples from the DC compartment of each of the eight arms of the PathoGut^TM^ SHIME experiment at defined time points (Figure 7) using selective media as described above. The absence of *C. difficile* in all PathoGut^TM^ SHIME arms before adding *C. difficile* spores was confirmed by plating DC samples of all arms at two timepoints during the C period. Furthermore, after the addition of *C. difficile* spores to the PC reactors during the CLI period, PC samples were cultured to confirm the addition of viable *C. difficile* cells. 

### 4.6. DNA Extraction

Samples from the PC and DC reactors of each of the eight arms of the PathoGut^TM^ SHIME experiment were obtained once per week from the C period onwards (Figure 7). DNA was extracted from 1 mL luminal sample using the Qiagen DNeasy UltraClean Microbial Kit, following manufacturer instructions (Qiagen, Antwerp, Belgium). The quality and quantity of the DNA samples were analyzed on 2% (*w*/*v*) agarose gel for 30 min at 100 V and spectrophotometrically by determination of the A260/A280 ratios (Synergy HT Microplate Reader, Agilent, Santa Clara, CA, USA).

### 4.7. Microbial Community Analysis by qPCR

Samples collected at defined time points (Figure 7) from the PC and DC reactors of each of the eight arms of the PathoGut^TM^ SHIME experiment were analyzed for quantification of Bacteroidetes and Firmicutes, *Enterobacteriaceae*, *Bifidobacterium* spp., *Lactobacillus* spp. and *Akkermansia muciniphila* using the primers and conditions described by Guo et al. [67], Nakano et al. [68], Rinttilä et al. [69], Furet et al. [70] and Collado et al. [71], respectively. qPCR was performed using a QuantStudio 5 Real-Time PCR system (Applied Biosystems, Foster City, CA, USA). Each sample was run in technical triplicate and results are reported as log (16S rRNA gene copies/mL).

### 4.8. Microbial Community Analysis by 16S rRNA Gene Sequencing

Microbial community composition was assessed at defined time points (Figure 7) from the PC and DC reactors of each of the eight arms of the PathoGut^TM^ SHIME experiment using 16S-targeted Illumina sequencing. Next-generation 16S rRNA gene amplicon sequencing of the V3–V4 region was performed by LGC Genomics GmbH (Berlin, Germany). Library preparation and sequencing were performed using an Illumina MiSeq platform with v3 chemistry. The 341F (5′-CCTACGGGNGGCWGCAG-3′) and 785R (5′-GACTACHVGGGTATCTAAKCC-3′) primers were used as previously described [72] with the reverse primer being adapted to increase coverage. Quality control PCR was conducted using Taq DNA Polymerase with the Fermentas PCR Kit according to the manufacturers’ instructions (Thermo Fisher Scientific, Waltham, MA, USA). Bioinformatic analysis of amplicon data was carried out as previously described [73].

### 4.9. Total Bacterial Count by Flow Cytometry

Total bacterial quantification was carried out at defined time points (Figure 7) for samples collected from the PC and DC reactors of each of the eight arms of the PathoGut^TM^ SHIME experiment using flow cytometry analysis. Briefly, 10-fold serial dilutions of the collected samples in PBS were stained with 0.01 mM SYTO24 (Life Technologies Europe, Merelbeke, Belgium) for 15 min at 37 °C in the dark. Samples were analyzed on a BD Facsverse (BDBiosciences, Erembodegem, Belgium) using the high-flowrate setting, and bacteria were separated from medium debris and signal noise by applying a threshold level of 200 on the SYTO channel. Electronic gating was defined based on previous experience with samples collected from the SHIME system. Flow cytometry data were analyzed using FlowJo, version 10.5.2 and reported as total bacterial counts in log units.

### 4.10. Metabolic Analysis

The parameters used to evaluate the activity of the gut microbiota in the PC and DC were monitored three times per week from the C period onwards (Figure 7). SCFA (acetate, propionate, and butyrate) and BCFA (isobutyrate, isovalerate, and isocaproate) were determined by gas chromatography (GC 2014-AOC 20i autosampler, Shimadzu Europa GmbH, Brussel, Belgium) as recently published [74]. Lactate production was assessed with a commercial kit (Enzytec^TM^ Liquid D-/L-Lactic acid kit (E8240), R-Biopharm, Darmstadt, Germany), according to manufacturer’s instructions. Ammonium quantification was performed using a KjelMaster K-375 (Büchi, Flawil, Switzerland), as described earlier [75]. Bile salts quantification (CA, chenodeoxycholic acid, DCA, TCA, TCDCA, GCA, glycochenodeoxycholic acid, lithocholic acid, ursodeoxycholic acid, glycodeoxycholic acid, taurodeoxycholic acid, and glycoursodeoxycholic acid) was performed on samples obtained from the DC reactors and was undertaken by high-performance liquid chromatography with a diode-array detector (Hitachi Chromaster HPLC-DAD, VWR, Belgium) as described by Jones et al. [76]. Only TCA, TCDCA, GCA, CA, and DCA were present in more than 80% of the samples and above the limit of quantification; thus, only these metabolites are reported. 

### 4.11. Data Analysis

Mean and standard error of the mean (SEM) for all treatment groups were used to summarize metabolic and qPCR data. Principal Component Analysis (PCA) plots were carried out applying the ClustVis webtool (http://biit.cs.ut.ee/clustvis/, accessed on 28 September 2022) [77]. Two-way ANOVA was used to calculate significant differences between treatment groups, employing GraphPad Prism version 8.2.0 for Windows (GraphPad Software, San Diego, CA, USA). The significance level of all statistical tests was set to 5%.

The 16S rRNA sequencing data were initially handled as described in De Paepe et al. [73]. To map the community shifts induced by the different test conditions, 16S-targeted Illumina sequencing data was adjusted to total bacteria quantification by flow cytometry [78]. Data at phylum, family, and genus levels were processed using Calyspo software version 8.84 (http://cgenome.net/calypso/, accessed on 28 September 2022) [79], removing samples with low abundance (<0.01%) and applying Hellinger transformation. 16S rRNA sequencing results from PC and DC were analyzed together, unless otherwise stated. DAPC and Adonis test based on Bray–Curtis distance were applied to evaluate the differences between groups, whereas LEfSe was used to determine specific features at family and genus level which were differentially enriched by the different test conditions. Significant differences between test conditions were assessed by a Mixed Effect Linear Regression model for repeated measures. 

## 5. Conclusions

Using a long-term exposure in vitro assay, our results showed that lactulose reduced the growth of viable *C. difficile* cells following CLI compared to the AB arm, shifted the antibiotic-induced dysbiotic microbial community and stimulated the production of SCFA, specifically the health-promoting metabolite butyrate. At a community level, the bifidogenic potential of lactulose has been demonstrated at different doses, together with a restoration of key commensal members of the gut ecosystem such as *Lactobacillaceae*, *Veillonellaceae* and *Lachnospiraceae*. These results suggest that long-term lactulose administration during antibiotic exposure and CDI might aid in recovering gut microenvironment, with potential benefits on immune modulation and host resistance to infection still to be determined in future studies. Furthermore, dosing lactulose at a concentration of 5 g/d showed promising potential in prevention of CDI recurrence upon co-administration with vancomycin, though human intervention studies are now warranted to confirm these findings. 

## Data Availability

Not applicable.

## Supplementary Materials

The following supporting information can be downloaded at: https://www.mdpi.com/article/10.3390/antibiotics11111464/s1, Figure S1: Total viable cell counts during the experiment; Figure S2: Microbial metabolic activity for the control arms; Figure S3: *C. difficile* quantification during the clindamycin treatment period; Figure S4: Base, lactate and ammonium levels in the CTRL, AB, LAC5 and LAC10 arms; Figure S5: Effect of lactulose and clindamycin on microbial metabolic activity; Figure S6: Distal SCFA levels for the CTRL, AB, LAC5 and LAC10 arms; Figure S7: Effect of lactulose and vancomycin on microbial metabolic activity; Figure S8: Base, lactate and ammonium levels in the CTRL, AB, LAC5V0, LAC10V0, LAC5V1 and LAC10V1 arms; Figure S9: Antibiotic effect as indicated in DAPC plot; Figure S10: Effect of antibiotics on microbial community composition; Figure S11: Effect of antibiotic treatment on microbial diversity; Figure S12: Abundance of OTU99 in the distal colon; Figure S13: qPCR results in the distal colon for the CTRL, AB, LAC5 and LAC10 arms; Figure S14: Effect of lactulose treatment on *Bifidobacteriaceae* levels during the post-intervention period; Figure S15: Effect on microbial community composition following vancomycin treatment; Figure S16: Effect of lactulose on microbial diversity following antibiotic treatment; Figure S17: qPCR results in the distal colon for the CTRL, AB, LAC5V0, LAC10V0, LAC5V1 and LAC10V1 arms; Table S1: Microbial community composition at phylum level for the control arms; Table S2: Microbial community composition at OTU level for the control arms; Table S3: Distal microbial community composition at family level for the AB and LAC10 arms; Table S4: Proximal microbial community composition at OTU level for the AB, LAC5 and LAC10 arms; Table S5: Distal microbial community composition at OTU level for the AB, LAC5 and LAC10 arms; Table S6: Proximal microbial community composition at family level for the AB and LAC10V0 arms; Table S7: Proximal microbial community composition at family level for the AB and LAC10V1 arms; Table S8: Distal microbial community composition at family level for the AB and LAC10V0 arms; Table S9: Distal microbial community composition at family level for the AB and LAC10V1 arms; Table S10: Proximal microbial community composition at OTU level for the LAC5V0, LAC10V0, LAC5V1 and LAC10V1 arms; Table S11: Distal microbial community composition at OTU level for the LAC5V0, LAC10V0, LAC5V1 and LAC10V1 arms.

## Abbreviations

AB	antibiotic-treated control
BCFA	branched-chain fatty acids
C	control (period)
CA	cholic acid
CDI	Clostridioides difficile infection
CFU	colony forming units
CLI	clindamycin treatment
COVID-19	coronavirus disease 19
CTRL	blank control
DAPC	discriminant analysis of principal components
DC	distal colon
DCA	deoxycholic acid
GCA	glycocholic acid
LAC5	lactulose treatment with 5 g/d during the clindamycin treatment and C. difficile stabilization period
LAC5V0	lactulose treatment with 5 g/d during the vancomycin treatment and post-intervention period
LAC5V1	lactulose treatment with 5 g/d during the vancomycin treatment and post-intervention period with lactulose supplementation initiated one day before starting vancomycin treatment
LAC10	lactulose treatment with 10 g/d during the clindamycin treatment and C. difficile infection stabilization period
LAC10V0	lactulose treatment with 10 g/d during the vancomycin treatment and post-intervention period
LAC10V1	lactulose treatment with 10 g/d during the vancomycin treatment and post-intervention period with lactulose supplementation initiated one day before starting vancomycin treatment
LDA	linear discriminant analysis
LEfSe	linear discriminant analysis effect size
PBS	phosphate buffer saline
PC	proximal colon
PCA	principal component analysis
PI	post-intervention
RCM	reinforced clostridial medium
SARS-CoV-2	severe acute respiratory syndrome coronavirus 2
SCFA	short-chain fatty acids
SEM	standard error of the mean
SHIME	Simulator of the Human Microbial Ecosytem
Si	small intestine
St	stomach
TCA	taurocholic acid
TCDCA	taurochenodeoxycholic acid
VNC	vancomycin treatment
_I	first week of
_II	second week of
_III	third week of
