# Supplementary material for: Long-Term Lactulose Administration Improves Dysbiosis Induced by Antibiotic and C. difficile in the PathoGutTM SHIME Model
Source: Antibiotics (Basel). 2022 Oct 24;11(11):1464. doi: 10.3390/antibiotics11111464 (PMC9686563; doi:10.3390/antibiotics11111464)

Long-term lactulose administration improves dysbiosis induced by antibiotic and *C. difficile* in the PathoGut™ SHIME model

Marta Calatayud, Cindy Duysburgh, Pieter Van den Abbeele, Dennis Franckenstein, Angelika Kuchina-Koch and Massimo Marzorati

Supplementary Materials

Table S1. Microbial community composition at phylum level for the control arms. Abundance of different phyla (log cells/mL) in arm 1 (CTRL) and arm 2 (AB) of the PathoGut™ SHIME experiment in the proximal (PC) and distal (DC) colon during the course of the control period (C_I-II), the clindamycin treatment period (CLI), the CDI stabilization period (CDI_I-III), the vancomycin treatment period (VNC) and the post-intervention period (PI_I-III) (n=1). The intensity of the shading indicates the absolute abundance, normalized for each of the different phyla (i.e., within each row).

**Phylum**	**PC**																			
	**CTRL**										**AB**									
	**C_I**	**C_II**	**CLI**	**CDI_I**	**CDI_II**	**CDI_III**	**VNC**	**PI_I**	**PI_II**	**PI_III**	**C_I**	**C_II**	**CLI**	**CDI_I**	**CDI_II**	**CDI_III**	**VNC**	**PI_I**	**PI_II**	**PI_III**
**Actinobacteria**	8.54	8.09	7.50	7.60	7.26	6.99	7.90	8.04	6.97	7.33	8.13	7.86	6.04	6.15	7.50	7.76	6.31	<LOQ	7.70	6.87
**Bacteroidetes**	8.98	9.03	8.94	8.79	8.80	8.83	7.99	8.08	8.64	9.01	8.98	8.88	8.63	8.75	8.57	9.03	<LOQ	8.24	8.54	8.92
**Desulfobacte-ria**	7.26	6.99	7.18	7.54	6.99	6.89	7.38	7.19	6.53	7.11	6.72	6.68	<LOQ	7.31	7.24	7.35	6.79	7.42	6.58	7.31
**Firmicutes**	8.71	8.71	8.81	8.80	8.81	8.75	8.91	8.20	8.33	8.69	8.64	8.86	6.17	8.46	7.81	8.51	6.56	6.05	5.85	6.61
**Proteobacteria**	7.77	7.37	7.54	8.13	7.79	8.60	8.84	9.00	8.85	8.57	7.68	7.46	8.87	8.41	8.43	9.25	8.52	9.13	8.86	8.71

**Phylum**	**DC**																			
	**CTRL**										**AB**									
	**C_I**	**C_II**	**CLI**	**CDI_I**	**CDI_II**	**CDI_III**	**VNC**	**PI_I**	**PI_II**	**PI_III**	**C_I**	**C_II**	**CLI**	**CDI_I**	**CDI_II**	**CDI_III**	**VNC**	**PI_I**	**PI_II**	**PI_III**
**Actinobacteria**	8.43	8.51	7.79	7.80	7.28	7.59	8.24	8.79	7.69	7.75	8.36	8.41	6.05	6.47	7.20	7.33	6.20	<LOQ	8.03	7.30
**Bacteroidetes**	9.01	8.98	8.93	8.72	8.59	8.76	8.20	8.69	9.00	8.94	8.77	8.87	8.50	8.96	8.85	8.82	<LOQ	7.64	8.88	9.08
**Desulfobacte-ria**	6.92	7.10	7.56	7.50	7.24	7.26	8.22	8.03	7.11	7.59	6.96	7.15	<LOQ	7.70	7.67	7.43	7.79	7.54	7.14	7.70
**Firmicutes**	8.76	8.68	8.70	8.58	8.48	8.64	8.93	8.31	8.61	8.55	8.81	8.85	7.33	8.66	8.67	8.78	6.60	5.92	7.72	8.14
**Proteobacteria**	7.55	7.56	7.84	8.25	7.88	7.75	8.27	7.65	7.71	8.01	7.59	7.63	8.81	8.43	8.20	8.73	8.52	9.09	8.65	8.64
**Verrucomicro-bia**	6.85	7.18	8.47	8.54	8.69	8.64	8.16	8.34	8.40	8.50	6.77	7.19	<LOQ	<LOQ	<LOQ	8.01	<LOQ	<LOQ	6.43	7.06

Table S2. Microbial community composition at OTU level for the control arms. Abundance of top 25 most abundant OTUs (log cells/mL) in arm 1 (CTRL) and arm 2 (AB) of the PathoGut™ SHIME experiment in the proximal (PC) and distal (DC) colon during the course of the control period (C_I-II), the clindamycin treatment period (CLI), the CDI stabilization period (CDI_I-III), the vancomycin treatment period (VNC) and the post-intervention period (PI_I-III) (n=1). The intensity of the shading indicates the absolute abundance, normalized for each of the different phyla (i.e., within each row).

OTU	Closely related species	PC																			
		CTRL										AB									
		C_I	C_II	CLI	CDI_I	CDI_II	CDI_III	VNC	PI_I	PI_II	PI_III	C_I	C_II	CLI	CDI_I	CDI_II	CDI_III	VNC	PI_I	PI_II	PI_III
**Otu00005**	***Bifidobacterium adolescentis***	8.53	8.08	7.42	7.52	7.07	6.89	7.85	7.92	6.91	7.28	8.11	7.84	<LOQ	<LOQ	<LOQ	<LOQ	<LOQ	<LOQ	<LOQ	<LOQ
**Otu00014**	***Bifidobacterium longum***	6.32	5.76	6.32	6.51	6.28	<LOQ	5.67	<LOQ	<LOQ	<LOQ	6.26	5.95	<LOQ	<LOQ	7.33	7.39	5.92	<LOQ	7.63	6.80
**Otu00013**	***Bacteroides fragilis***	7.56	7.46	7.87	7.27	7.51	7.32	6.54	6.90	7.16	7.71	7.12	6.87	<LOQ	<LOQ	<LOQ	6.88	<LOQ	8.18	8.50	8.82
**Otu00003**	***Bacteroides thetaiotaomicron***	8.45	7.04	6.94	6.06	7.38	7.54	6.54	6.23	6.62	7.17	8.67	8.53	7.37	8.36	7.73	8.30	<LOQ	6.28	7.05	8.07
**Otu00004**	***Bacteroides ovatus***	8.29	8.78	8.69	8.20	8.20	8.18	7.13	7.18	7.69	8.14	8.00	8.33	8.39	7.36	7.75	7.41	<LOQ	7.21	<LOQ	6.89
**Otu00006**	***Bacteroides dorei***	7.74	8.26	8.14	8.36	8.25	8.41	7.58	7.83	8.46	8.75	7.56	7.48	7.48	7.61	8.09	8.50	<LOQ	<LOQ	6.39	6.85
**Otu00025**	***Bacteroides finegoldii***	<LOQ	<LOQ	<LOQ	<LOQ	<LOQ	<LOQ	<LOQ	<LOQ	<LOQ	<LOQ	<LOQ	<LOQ	<LOQ	<LOQ	<LOQ	<LOQ	<LOQ	<LOQ	<LOQ	<LOQ
**Otu00020**	***Bacteroides salyersiae***	6.81	6.71	6.76	6.48	6.77	6.60	<LOQ	5.66	5.92	6.66	7.14	7.09	<LOQ	<LOQ	<LOQ	<LOQ	<LOQ	<LOQ	<LOQ	<LOQ
**Otu00012**	***Paraprevotella clara***	6.27	6.97	7.75	7.97	7.82	7.86	7.20	7.01	7.40	8.07	6.70	6.57	7.92	7.98	7.84	8.46	<LOQ	<LOQ	<LOQ	<LOQ
**Otu00017**	***Paraprevotella clara***	5.97	6.49	7.29	7.61	7.40	7.49	6.76	6.54	7.02	7.64	6.42	6.02	7.49	7.57	7.44	8.04	<LOQ	<LOQ	<LOQ	<LOQ
**Otu00030**	***Alistipes finegoldii/onderdonkii***	6.06	5.76	6.33	6.45	6.48	6.08	5.67	<LOQ	<LOQ	<LOQ	<LOQ	<LOQ	<LOQ	<LOQ	<LOQ	<LOQ	<LOQ	<LOQ	<LOQ	<LOQ
**Otu00015**	***Parabacteroides distasonis***	7.33	7.08	6.96	7.39	7.49	7.19	6.74	6.06	6.55	7.39	7.11	6.91	<LOQ	8.13	7.58	8.05	<LOQ	<LOQ	6.48	7.53
**Otu00016**	***Bilophila wadsworthia***	7.26	6.99	7.18	7.54	6.99	6.89	7.38	7.18	6.53	7.11	6.72	6.68	<LOQ	7.31	7.24	7.35	6.79	7.41	6.56	7.31
**Otu00001**	***Clostridium clos-tridioforme/bolteae***	8.49	8.58	8.68	8.63	8.61	8.46	8.47	7.22	7.95	8.20	8.44	8.75	<LOQ	8.41	7.55	7.07	<LOQ	<LOQ	<LOQ	<LOQ
**Otu00022**	***Clostridium aldenense***	5.84	5.68	<LOQ	5.68	5.88	5.75	5.83	<LOQ	<LOQ	<LOQ	5.72	5.75	<LOQ	7.45	7.37	7.74	<LOQ	<LOQ	<LOQ	<LOQ
**Otu00027**	***Hungatella hathewayi***	<LOQ	<LOQ	<LOQ	<LOQ	<LOQ	<LOQ	<LOQ	<LOQ	<LOQ	<LOQ	<LOQ	<LOQ	<LOQ	<LOQ	<LOQ	<LOQ	<LOQ	<LOQ	<LOQ	<LOQ
**Otu00031**	***Faecalibacterium prausnitzii***	<LOQ	<LOQ	<LOQ	<LOQ	<LOQ	<LOQ	<LOQ	<LOQ	<LOQ	<LOQ	<LOQ	<LOQ	<LOQ	<LOQ	<LOQ	<LOQ	<LOQ	<LOQ	<LOQ	<LOQ
**Otu00032**	***Selenomonas infelix***	5.84	5.68	5.80	5.90	6.87	7.73	8.47	7.05	6.77	7.96	6.02	5.69	<LOQ	<LOQ	<LOQ	<LOQ	<LOQ	<LOQ	<LOQ	<LOQ
**Otu00007**	***Veillonella dispar***	6.54	6.26	6.00	6.08	6.04	5.63	5.94	<LOQ	<LOQ	<LOQ	<LOQ	<LOQ	<LOQ	<LOQ	<LOQ	<LOQ	<LOQ	<LOQ	<LOQ	<LOQ
**Otu00028**	***Megasphaera sp.***	5.67	6.06	5.80	6.38	5.98	5.89	5.73	<LOQ	5.83	6.38	<LOQ	5.69	<LOQ	<LOQ	<LOQ	<LOQ	<LOQ	<LOQ	<LOQ	<LOQ
**Otu00002**	***Klebsiella pneumoniae***	<LOQ	<LOQ	<LOQ	<LOQ	<LOQ	<LOQ	6.54	6.61	6.73	7.70	<LOQ	<LOQ	<LOQ	8.16	7.98	8.73	7.98	8.89	8.32	8.42

**Otu00011**	*Klebsiella oxytoca*	<LOQ	<LOQ	<LOQ	<LOQ	<LOQ	<LOQ	<LOQ	<LOQ	<LOQ	<LOQ	<LOQ	<LOQ	<LOQ	6.39	6.13	8.30	<LOQ	7.04	6.12	6.29
**Otu00008**	*Citrobacter freundii*	<LOQ	<LOQ	<LOQ	6.06	7.47	8.57	8.81	9.00	8.83	8.40	5.96	6.37	7.35	7.53	8.12	8.76	8.30	8.73	8.70	8.36
**Otu00009**	*Escherichia coli/Shigella sonnei*	<LOQ	<LOQ	<LOQ	6.04	6.01	6.19	6.91	<LOQ	6.37	7.20	5.72	<LOQ	8.84	7.46	6.96	7.00	<LOQ	6.39	6.18	6.58
**Otu00010**	*Akkermansia muciniphila*	<LOQ	<LOQ	<LOQ	<LOQ	<LOQ	<LOQ	<LOQ	<LOQ	<LOQ	<LOQ	<LOQ	<LOQ	<LOQ	<LOQ	<LOQ	<LOQ	<LOQ	<LOQ	<LOQ	<LOQ

**OTU**	**Closely related species**	**DC**																			
		**CTRL**										**AB**									
		**C_I**	**C_II**	**CLI**	**CDI_I**	**CDI_II**	**CDI_III**	**VNC**	**PI_I**	**PI_II**	**PI_III**	**C_I**	**C_II**	**CLI**	**CDI_I**	**CDI_II**	**CDI_III**	**VNC**	**PI_I**	**PI_II**	**PI_III**
**Otu00005**	*Bifidobacterium adolescentis*	8.42	8.50	7.72	7.74	7.06	7.55	8.20	8.76	7.64	7.73	8.34	8.39	<LOQ	<LOQ	<LOQ	<LOQ	<LOQ	<LOQ	<LOQ	<LOQ
**Otu00014**	*Bifidobacterium longum*	6.07	5.88	6.51	6.43	6.19	5.93	6.35	6.08	<LOQ	<LOQ	6.28	6.54	<LOQ	<LOQ	6.86	7.11	5.89	<LOQ	7.97	7.21
**Otu00013**	*Bacteroides fragilis*	6.99	6.88	7.58	7.04	6.90	6.78	6.83	7.34	7.39	7.52	6.70	6.78	<LOQ	<LOQ	7.29	7.76	<LOQ	7.58	8.72	8.70
**Otu00003**	*Bacteroides thetaiotaomicron*	8.57	7.54	7.87	7.99	7.30	7.66	6.47	7.73	8.00	7.72	8.41	8.22	7.79	8.69	8.50	8.00	<LOQ	5.66	8.00	8.66
**Otu00004**	*Bacteroides ovatus*	8.37	8.69	8.44	8.02	7.93	8.10	7.24	8.21	8.51	8.23	7.83	8.18	7.80	7.54	8.01	7.44	<LOQ	6.42	7.71	7.80
**Otu00006**	*Bacteroides dorei*	7.83	7.89	7.97	8.16	7.92	8.12	7.74	7.86	8.28	8.49	7.69	7.69	8.23	7.75	7.89	8.33	<LOQ	5.68	7.52	7.94
**Otu00025**	*Bacteroides finegoldii*	<LOQ	<LOQ	<LOQ	<LOQ	<LOQ	<LOQ	<LOQ	<LOQ	<LOQ	<LOQ	<LOQ	<LOQ	<LOQ	<LOQ	<LOQ	<LOQ	<LOQ	<LOQ	<LOQ	<LOQ
**Otu00020**	*Bacteroides salyersiae*	6.61	5.70	6.21	6.18	5.76	6.30	6.03	6.22	6.59	6.73	6.43	6.20	<LOQ	<LOQ	6.30	7.72	<LOQ	<LOQ	<LOQ	<LOQ
**Otu00012**	*Paraprevotella clara*	5.89	5.70	5.67	<LOQ	<LOQ	<LOQ	<LOQ	<LOQ	<LOQ	6.16	5.98	6.38	6.34	7.09	6.28	6.26	<LOQ	<LOQ	<LOQ	<LOQ
**Otu00017**	*Paraprevotella clara*	<LOQ	5.70	<LOQ	<LOQ	<LOQ	<LOQ	<LOQ	<LOQ	5.85	5.97	<LOQ	6.10	6.00	6.70	6.08	6.22	<LOQ	<LOQ	<LOQ	<LOQ
**Otu00030**	*Alistipes finegoldii/onderdonkii*	5.77	5.88	6.02	6.99	7.56	7.69	7.01	7.24	7.61	7.67	5.88	5.98	<LOQ	<LOQ	<LOQ	<LOQ	<LOQ	<LOQ	<LOQ	<LOQ
**Otu00015**	*Parabacteroides distasonis*	7.26	7.09	7.24	7.86	7.74	7.85	6.80	7.45	7.91	8.01	7.54	7.38	<LOQ	7.94	7.31	7.65	<LOQ	5.91	6.69	7.81
**Otu00016**	*Bilophila wadsworthia*	6.87	6.99	7.51	7.47	7.20	7.21	8.07	8.01	7.00	7.52	6.85	7.07	<LOQ	7.62	7.64	7.40	7.79	7.49	7.03	7.67
**Otu00001**	*Clostridium clos-tridioforme/bolteae*	8.61	8.50	8.54	8.41	8.32	8.45	8.70	8.00	8.28	8.20	8.67	8.68	<LOQ	8.51	8.09	8.03	<LOQ	<LOQ	6.71	7.63
**Otu00022**	*Clostridium aldenense*	6.55	6.31	6.34	6.27	6.39	6.49	7.07	6.46	6.16	6.46	6.79	6.63	<LOQ	7.43	7.70	7.80	<LOQ	<LOQ	<LOQ	6.47
**Otu00027**	*Hungatella hathewayi*	6.14	<LOQ	6.29	6.05	5.86	6.02	5.93	<LOQ	5.85	5.83	6.18	5.98	<LOQ	7.38	8.30	8.23	<LOQ	<LOQ	<LOQ	<LOQ
**Otu00031**	*Faecalibacterium prausnitzii*	<LOQ	5.70	<LOQ	<LOQ	<LOQ	<LOQ	<LOQ	<LOQ	<LOQ	<LOQ	<LOQ	<LOQ	5.81	6.72	6.50	6.66	<LOQ	<LOQ	<LOQ	<LOQ
**Otu00032**	*Selenomonas infelix*	<LOQ	<LOQ	<LOQ	<LOQ	<LOQ	6.54	7.95	6.67	5.85	7.23	<LOQ	<LOQ	<LOQ	<LOQ	<LOQ	<LOQ	<LOQ	<LOQ	<LOQ	<LOQ
**Otu00007**	*Veillonella dispar*	<LOQ	5.88	<LOQ	<LOQ	<LOQ	<LOQ	<LOQ	<LOQ	<LOQ	<LOQ	<LOQ	<LOQ	<LOQ	<LOQ	<LOQ	<LOQ	<LOQ	<LOQ	<LOQ	<LOQ
**Otu00028**	*Megasphaera sp.*	6.69	6.61	6.32	6.76	5.67	5.81	6.19	5.66	6.46	6.70	6.18	6.48	<LOQ	<LOQ	<LOQ	7.68	<LOQ	<LOQ	<LOQ	<LOQ
**Otu00002**	*Klebsiella pneumoniae*	<LOQ	<LOQ	<LOQ	<LOQ	<LOQ	<LOQ	6.27	5.96	6.16	6.56	<LOQ	<LOQ	6.45	7.91	7.63	8.17	7.13	8.80	7.89	8.41
**Otu00011**	*Klebsiella oxytoca*	<LOQ	<LOQ	<LOQ	<LOQ	<LOQ	<LOQ	5.66	<LOQ	<LOQ	5.97	<LOQ	5.68	6.00	5.78	5.70	7.49	8.34	7.93	7.19	6.30

**Otu00008**	*Citrobacter freundii*	<LOQ	<LOQ	<LOQ	5.78	<LOQ	6.51	7.93	7.42	7.06	6.53	<LOQ	<LOQ	7.59	7.31	7.20	8.08	7.87	8.69	8.49	8.06
**Otu00009**	*Escherichia coli/Shigella sonnei*	<LOQ	<LOQ	<LOQ	5.75	5.86	5.74	6.93	5.66	6.03	5.93	<LOQ	<LOQ	8.76	7.45	6.64	6.76	<LOQ	6.72	7.06	6.69
**Otu00010**	*Akkermansia muciniphila*	6.85	7.18	8.47	8.54	8.69	8.64	8.15	8.34	8.39	8.49	6.74	7.17	<LOQ	<LOQ	<LOQ	7.96	<LOQ	<LOQ	<LOQ	<LOQ

Table S3. Distal microbial community composition at family level for the AB and LAC10 arms. Abundance at family level (log cells/mL) in arm 2 (AB) and arm 4 (LAC10) of the PathoGut™ SHIME experiment in the distal colon (DC) during the course of the control period (C_I-II), the clindamycin treatment period (CLI) and the CDI stabilization period (CDI_I-III) (n=1). The intensity of the shading indicates the absolute abundance, normalized for each of the different phyla (i.e., within each row).

Phylum	Family	DC											
		AB						LAC10					
		C_I	C_II	CLI	CDI_I	CDI_II	CDI_III	C_I	C_II	CLI	CDI_I	CDI_II	CDI_III
Actino-bacteria	*Bifidobacteriaceae*	8.36	8.41	<LOQ	<LOQ	6.91	7.19	8.47	8.18	<LOQ	7.58	8.67	8.76
	Coriobacteriaceae	<LOQ	5.80	<LOQ	<LOQ	<LOQ	<LOQ	5.70	5.64	<LOQ	6.21	6.03	6.20
	Microbacteriaceae	<LOQ	5.68	6.05	6.46	6.89	6.75	5.70	6.12	<LOQ	<LOQ	<LOQ	<LOQ
Bac-teroide-tes	*Bacteroidaceae*	8.73	8.84	8.49	8.90	8.82	8.77	8.88	8.93	9.19	8.32	8.31	8.53
	Marinifilaceae	<LOQ	<LOQ	<LOQ	<LOQ	<LOQ	5.64	6.05	6.34	<LOQ	5.64	6.09	5.82
	Muribaculaceae	6.43	6.05	<LOQ	<LOQ	<LOQ	<LOQ	5.87	6.38	<LOQ	<LOQ	<LOQ	<LOQ
	Prevotellaceae	6.06	6.58	6.53	7.24	6.50	6.54	6.98	7.56	8.77	<LOQ	7.43	6.47
	Rikenellaceae	6.32	6.38	<LOQ	7.44	6.85	6.64	6.74	7.01	<LOQ	7.52	8.30	8.21
	Tannerellaceae	7.67	7.48	<LOQ	7.94	7.50	7.84	7.34	7.22	<LOQ	7.79	8.15	8.08
Desul-fobacte-ria	*Desulfovibri-onaceae*	6.96	7.15	<LOQ	7.70	7.67	7.43	7.20	6.96	<LOQ	7.48	7.19	7.25
Firmicu-tes	*Acidaminococca-ceae*	6.89	7.01	<LOQ	<LOQ	<LOQ	<LOQ	6.82	6.91	<LOQ	<LOQ	<LOQ	<LOQ
	Anaerovoracaceae	6.46	5.90	<LOQ	6.70	6.29	6.26	6.30	6.48	<LOQ	<LOQ	<LOQ	<LOQ
	Butyricicoccaceae	<LOQ	5.98	6.30	5.73	5.82	5.98	5.70	<LOQ	<LOQ	<LOQ	<LOQ	<LOQ
	Carnobacteriaceae	<LOQ	5.98	6.08	<LOQ	6.12	6.14	<LOQ	6.18	<LOQ	<LOQ	<LOQ	<LOQ
	Clostridiaceae	<LOQ	<LOQ	<LOQ	6.55	5.73	6.14	<LOQ	<LOQ	<LOQ	<LOQ	<LOQ	<LOQ
	Enterococcaceae	5.88	5.80	6.15	6.43	5.91	7.01	5.87	5.94	<LOQ	<LOQ	<LOQ	<LOQ
	Erysipelotrichaceae	<LOQ	<LOQ	<LOQ	5.64	<LOQ	5.89	<LOQ	<LOQ	<LOQ	<LOQ	6.84	6.85
	Eubacteriaceae	6.23	6.28	<LOQ	<LOQ	6.54	6.02	6.09	6.45	<LOQ	6.54	7.38	7.23
	Lachnospiraceae	8.79	8.83	6.27	8.63	8.65	8.72	8.56	8.69	<LOQ	8.92	8.69	8.68
	Lactobacillaceae	<LOQ	<LOQ	<LOQ	<LOQ	<LOQ	5.89	<LOQ	<LOQ	<LOQ	6.67	6.46	6.27
	Oscillospiraceae	6.98	6.91	7.13	6.98	6.86	6.72	6.90	7.14	7.50	7.20	6.77	6.96
	Oscillospirales_fa	6.28	6.38	<LOQ	6.01	5.79	<LOQ	6.40	6.45	<LOQ	<LOQ	<LOQ	<LOQ
	Ruminococcaceae	6.56	6.35	6.09	7.18	6.75	7.17	6.47	6.91	8.83	6.47	5.90	6.38
	Veillonellaceae	6.60	6.54	<LOQ	<LOQ	<LOQ	7.68	6.66	6.48	<LOQ	7.86	8.23	8.56
Proteo-bacteria	*Alcaligenaceae*	<LOQ	6.24	6.02	6.48	5.73	7.14	5.79	<LOQ	5.66	<LOQ	<LOQ	<LOQ
	Enterobacterales_ unclassified	<LOQ	<LOQ	5.72	<LOQ	<LOQ	6.12	<LOQ	<LOQ	6.57	<LOQ	<LOQ	<LOQ
	Enterobacteriaceae	<LOQ	5.90	8.79	8.12	7.81	8.49	5.79	5.64	9.01	6.38	6.96	7.31
	Pseudomonadaceae	7.06	7.17	7.35	7.88	7.61	7.99	7.17	7.30	7.19	6.59	6.45	6.33
	Sutterellaceae	7.11	7.07	<LOQ	7.62	7.57	7.39	7.18	7.35	<LOQ	7.13	7.46	7.37
	uncultured	7.11	7.13	<LOQ	7.13	7.08	7.87	7.15	7.36	<LOQ	7.48	5.98	5.90
	Xanthomonadaceae	<LOQ	<LOQ	6.18	6.47	6.05	7.19	<LOQ	5.94	<LOQ	<LOQ	<LOQ	<LOQ
Verru-comicro-bia	*Akkermansiaceae*	6.74	7.17	<LOQ	<LOQ	<LOQ	7.97	6.76	7.26	<LOQ	8.06	7.59	7.65
	Victivallaceae	<LOQ	<LOQ	<LOQ	<LOQ	<LOQ	6.96	<LOQ	<LOQ	<LOQ	6.68	6.32	6.13

Table S4. Proximal microbial community composition at OTU level for the AB, LAC5 and LAC10 arms. Abundance of top 25 most abundant OTUs (log cells/mL) in arm 2 (AB), arm 3 (LAC5) and arm 4 (LAC10) of the PathoGut™ SHIME experiment in the proximal colon (PC) during the course of the control period (C_I-II), the clindamycin treatment period (CLI) and the CDI stabilization period (CDI_I-III) (n=1). The intensity of the shading indicates the absolute abundance, normalized for each of the different phyla (i.e., within each row).

OTU	Closely related species	PC																	
		AB						LAC5						LAC10					
		C_I	C_II	CLI	CDI_I	CDI_II	CDI_III	C_I	C_II	CLI	CDI_I	CDI_II	CDI_III	C_I	C_II	CLI	CDI_I	CDI_II	CDI_III
**Otu00005**	*Bifidobacterium adolescentis*	8.11	7.84	<LOQ	<LOQ	<LOQ	<LOQ	8.05	7.76	<LOQ	8.66	8.58	8.82	8.52	7.67	<LOQ	7.48	8.44	8.81
**Otu00014**	*Bifidobacterium longum*	6.26	5.95	<LOQ	<LOQ	7.33	7.39	<LOQ	5.86	<LOQ	6.90	7.28	6.41	6.48	5.74	<LOQ	<LOQ	<LOQ	<LOQ
**Otu00013**	*Bacteroides fragilis*	7.12	6.87	<LOQ	<LOQ	<LOQ	6.88	6.66	7.55	<LOQ	8.35	7.10	7.48	7.83	8.49	<LOQ	<LOQ	<LOQ	6.70
**Otu00003**	*Bacteroides thetaiotaomicron*	8.67	8.53	7.37	8.36	7.73	8.30	8.64	8.65	7.38	6.70	6.63	6.52	8.47	7.70	6.70	<LOQ	<LOQ	<LOQ
**Otu00004**	*Bacteroides ovatus*	8.00	8.33	8.39	7.36	7.75	7.41	8.56	8.75	9.19	8.39	7.04	6.20	8.23	8.70	8.63	<LOQ	<LOQ	<LOQ
**Otu00006**	*Bacteroides dorei*	7.56	7.48	7.48	7.61	8.09	8.50	7.51	7.41	7.37	7.18	7.68	8.36	7.59	7.94	6.34	<LOQ	6.32	<LOQ
**Otu00025**	*Bacteroides finegoldii*	<LOQ	<LOQ	<LOQ	<LOQ	<LOQ	<LOQ	<LOQ	<LOQ	<LOQ	<LOQ	<LOQ	<LOQ	<LOQ	6.10	<LOQ	<LOQ	<LOQ	<LOQ
**Otu00020**	*Bacteroides salyersiae*	7.14	7.09	<LOQ	<LOQ	<LOQ	<LOQ	7.00	6.96	7.33	7.78	8.41	7.91	7.04	6.74	6.60	<LOQ	<LOQ	<LOQ
**Otu00012**	*Paraprevotella clara*	6.70	6.57	7.92	7.98	7.84	8.46	6.87	7.20	8.49	7.92	7.75	7.78	6.33	7.15	8.71	8.54	7.97	8.20
**Otu00017**	*Paraprevotella clara*	6.42	6.02	7.49	7.57	7.44	8.04	6.51	6.72	8.06	7.49	7.32	7.35	<LOQ	6.66	8.28	8.12	7.58	7.78
**Otu00030**	*Alistipes finegoldii/onderdon-kii*	<LOQ	<LOQ	<LOQ	<LOQ	<LOQ	<LOQ	<LOQ	<LOQ	<LOQ	<LOQ	<LOQ	<LOQ	6.26	<LOQ	<LOQ	<LOQ	<LOQ	<LOQ
**Otu00015**	*Parabacteroides distasonis*	7.11	6.91	<LOQ	8.13	7.58	8.05	7.15	7.08	<LOQ	7.00	6.90	6.78	7.58	6.61	<LOQ	7.71	<LOQ	<LOQ
**Otu00016**	*Bilophila wadsworthia*	6.72	6.68	<LOQ	7.31	7.24	7.35	6.86	6.84	<LOQ	<LOQ	6.52	6.26	7.16	7.03	<LOQ	<LOQ	<LOQ	<LOQ
**Otu00001**	*Clostridium clos-tridioforme/bolteae*	8.44	8.75	<LOQ	8.41	7.55	7.07	8.54	8.47	<LOQ	8.40	7.14	6.13	8.57	8.51	<LOQ	8.40	7.97	7.94
**Otu00022**	*Clostridium aldenense*	5.72	5.75	<LOQ	7.45	7.37	7.74	6.06	<LOQ	<LOQ	<LOQ	5.89	6.62	5.78	<LOQ	<LOQ	<LOQ	<LOQ	<LOQ
**Otu00027**	*Hungatella hathewayi*	<LOQ	<LOQ	<LOQ	<LOQ	<LOQ	<LOQ	<LOQ	<LOQ	<LOQ	<LOQ	<LOQ	<LOQ	<LOQ	<LOQ	<LOQ	<LOQ	<LOQ	<LOQ
**Otu00031**	*Faecalibacterium prausnitzii*	<LOQ	<LOQ	<LOQ	<LOQ	<LOQ	<LOQ	<LOQ	<LOQ	<LOQ	<LOQ	<LOQ	<LOQ	<LOQ	<LOQ	<LOQ	<LOQ	<LOQ	<LOQ
**Otu00032**	*Selenomonas infelix*	6.02	5.69	<LOQ	<LOQ	<LOQ	<LOQ	<LOQ	<LOQ	<LOQ	<LOQ	<LOQ	<LOQ	6.18	<LOQ	<LOQ	<LOQ	<LOQ	<LOQ
**Otu00007**	*Veillonella dispar*	<LOQ	<LOQ	<LOQ	<LOQ	<LOQ	<LOQ	<LOQ	<LOQ	<LOQ	8.09	7.63	8.55	5.96	<LOQ	<LOQ	8.09	8.20	8.76
**Otu00028**	*Megasphaera sp.*	<LOQ	5.69	<LOQ	<LOQ	<LOQ	<LOQ	<LOQ	<LOQ	<LOQ	7.97	7.56	7.11	5.78	5.85	<LOQ	<LOQ	<LOQ	<LOQ
**Otu00002**	*Klebsiella pneumoniae*	<LOQ	<LOQ	<LOQ	8.16	7.98	8.73	7.22	6.00	8.41	7.08	6.95	7.18	<LOQ	<LOQ	7.85	6.62	6.84	6.93
**Otu00011**	*Klebsiella oxytoca*	<LOQ	<LOQ	<LOQ	6.39	6.13	8.30	<LOQ	<LOQ	<LOQ	<LOQ	<LOQ	6.21	<LOQ	<LOQ	6.09	5.67	5.72	5.66
**Otu00008**	*Citrobacter freundii*	5.96	6.37	7.35	7.53	8.12	8.76	6.36	<LOQ	8.01	6.57	7.38	7.93	6.74	6.28	7.40	5.74	7.39	8.11
**Otu00009**	*Escherichia coli/Shigella sonnei*	5.72	<LOQ	8.84	7.46	6.96	7.00	<LOQ	<LOQ	8.14	5.83	<LOQ	<LOQ	<LOQ	<LOQ	8.53	<LOQ	<LOQ	<LOQ
**Otu00010**	*Akkermansia muciniphila*	<LOQ	<LOQ	<LOQ	<LOQ	<LOQ	<LOQ	<LOQ	<LOQ	<LOQ	<LOQ	<LOQ	<LOQ	<LOQ	<LOQ	<LOQ	<LOQ	<LOQ	<LOQ

Table S5. Distal microbial community composition at OTU level for the AB, LAC5 and LAC10 arms. Abundance of top 25 most abundant OTUs (log cells/mL) in arm 2 (AB), arm 3 (LAC5) and arm 4 (LAC10) of the PathoGut™ SHIME experiment in the distal colon (DC) during the course of the control period (C_I-II), the clindamycin treatment period (CLI) and the CDI stabilization period (CDI_I-III) (n=1). The intensity of the shading indicates the absolute abundance, normalized for each of the different phyla (i.e., within each row).

OTU	Closely related species	DC																	
		AB						LAC5						LAC10					
		C_I	C_II	CLI	CDI_I	CDI_II	CDI_III	C_I	C_II	CLI	CDI_I	CDI_II	CDI_III	C_I	C_II	CLI	CDI_I	CDI_II	CDI_III
**Otu00005**	***Bifidobacterium adolescentis***	8.34	8.39	<LOQ	<LOQ	<LOQ	<LOQ	8.16	8.15	<LOQ	8.63	8.42	8.71	8.46	8.16	<LOQ	7.58	8.66	8.76
**Otu00014**	***Bifidobacterium longum***	6.28	6.54	<LOQ	<LOQ	6.86	7.11	6.16	5.70	<LOQ	7.04	6.97	6.28	6.24	5.82	<LOQ	<LOQ	<LOQ	<LOQ
**Otu00013**	***Bacteroides fragilis***	6.70	6.78	<LOQ	<LOQ	7.29	7.76	6.24	7.11	5.83	7.96	6.76	7.82	6.82	7.96	6.49	<LOQ	<LOQ	6.00
**Otu00003**	***Bacteroides thetaiotaomicron***	8.41	8.22	7.79	8.69	8.50	8.00	8.24	8.40	7.98	7.35	7.88	7.50	8.35	7.86	7.99	7.83	7.81	6.83
**Otu00004**	***Bacteroides ovatus***	7.83	8.18	7.80	7.54	8.01	7.44	8.09	8.49	8.75	7.68	7.21	7.06	8.02	8.39	9.14	6.95	7.34	6.51
**Otu00006**	***Bacteroides dorei***	7.69	7.69	8.23	7.75	7.89	8.33	7.98	7.65	8.76	7.57	7.80	8.29	8.00	7.95	6.60	7.75	7.43	8.43
**Otu00025**	***Bacteroides finegoldii***	<LOQ	<LOQ	<LOQ	<LOQ	<LOQ	<LOQ	<LOQ	<LOQ	<LOQ	<LOQ	<LOQ	<LOQ	<LOQ	<LOQ	<LOQ	<LOQ	<LOQ	<LOQ
**Otu00020**	***Bacteroides salyersiae***	6.43	6.20	<LOQ	<LOQ	6.30	7.72	6.06	5.83	8.06	6.72	7.91	7.78	5.87	5.64	6.73	<LOQ	7.14	6.87
**Otu00012**	***Paraprevotella clara***	5.98	6.38	6.34	7.09	6.28	6.26	6.16	6.40	7.48	5.85	5.70	6.17	6.81	7.40	8.62	<LOQ	7.27	6.34
**Otu00017**	***Paraprevotella clara***	<LOQ	6.10	6.00	6.70	6.08	6.22	<LOQ	5.70	6.97	<LOQ	<LOQ	5.92	6.47	7.01	8.21	<LOQ	6.91	5.87
**Otu00030**	***Alistipes finegoldii/onderdonkii***	5.88	5.98	<LOQ	<LOQ	<LOQ	<LOQ	6.01	6.40	<LOQ	5.72	7.10	7.06	6.42	6.87	<LOQ	6.25	8.29	8.20
**Otu00015**	***Parabacteroides distasonis***	7.54	7.38	<LOQ	7.94	7.31	7.65	7.05	7.23	8.20	7.49	7.27	7.69	7.23	7.06	<LOQ	7.56	7.54	7.50
**Otu00016**	***Bilophila wadsworthia***	6.85	7.07	<LOQ	7.62	7.64	7.40	6.99	7.14	<LOQ	7.48	6.56	6.62	7.12	6.87	<LOQ	7.41	7.09	7.15
**Otu00001**	***Clostridium clostridioforme/bolteae***	8.67	8.68	<LOQ	8.51	8.09	8.03	8.70	8.55	5.61	8.03	7.67	7.75	8.41	8.52	<LOQ	8.86	8.53	8.47
**Otu00022**	***Clostridium aldenense***	6.79	6.63	<LOQ	7.43	7.70	7.80	6.44	6.67	<LOQ	6.64	7.19	7.56	6.37	6.66	<LOQ	6.20	7.21	7.11
**Otu00027**	***Hungatella hathewayi***	6.18	5.98	<LOQ	7.38	8.30	8.23	6.20	6.00	<LOQ	6.45	6.34	6.62	5.70	6.29	<LOQ	5.95	6.07	5.82
**Otu00031**	***Faecalibacterium prausnitzii***	<LOQ	<LOQ	5.81	6.72	6.50	6.66	<LOQ	<LOQ	8.05	<LOQ	<LOQ	<LOQ	<LOQ	<LOQ	8.83	<LOQ	<LOQ	<LOQ
**Otu00032**	***Selenomonas infelix***	<LOQ	<LOQ	<LOQ	<LOQ	<LOQ	<LOQ	<LOQ	<LOQ	<LOQ	<LOQ	<LOQ	<LOQ	<LOQ	<LOQ	<LOQ	<LOQ	<LOQ	<LOQ
**Otu00007**	***Veillonella dispar***	<LOQ	<LOQ	<LOQ	<LOQ	<LOQ	<LOQ	<LOQ	<LOQ	<LOQ	8.33	7.84	8.47	<LOQ	<LOQ	<LOQ	7.85	8.23	8.56
**Otu00028**	***Megasphaera sp.***	6.18	6.48	<LOQ	<LOQ	<LOQ	7.68	5.94	6.50	<LOQ	7.59	7.15	7.52	5.87	6.12	<LOQ	<LOQ	<LOQ	<LOQ
**Otu00002**	***Klebsiella pneumoniae***	<LOQ	<LOQ	6.45	7.91	7.63	8.17	<LOQ	5.83	8.48	6.69	6.35	6.44	<LOQ	<LOQ	8.11	6.15	6.11	6.22
**Otu00011**	***Klebsiella oxytoca***	<LOQ	5.68	6.00	5.78	5.70	7.49	<LOQ	<LOQ	5.61	<LOQ	<LOQ	<LOQ	<LOQ	<LOQ	6.45	<LOQ	<LOQ	<LOQ
**Otu00008**	***Citrobacter freundii***	<LOQ	<LOQ	7.59	7.31	7.20	8.08	<LOQ	<LOQ	8.12	6.28	6.33	6.84	<LOQ	5.64	7.94	<LOQ	6.54	7.22
**Otu00009**	***Escherichia coli/Shigella sonnei***	<LOQ	<LOQ	8.76	7.45	6.64	6.76	<LOQ	<LOQ	8.90	6.39	6.03	5.62	<LOQ	<LOQ	8.87	5.78	6.60	6.30
**Otu00010**	***Akkermansia muciniphila***	6.74	7.17	<LOQ	<LOQ	<LOQ	7.96	6.58	7.06	<LOQ	9.02	9.05	8.68	6.76	7.26	<LOQ	8.05	7.59	7.65

Table S6. Proximal microbial community composition at family level for the AB and LAC10V0 arms. Abundance of different families (log cells/mL) in arm 2 (AB) and arm 6 (LAC10V0) of the PathoGut™ SHIME experiment in the proximal colon (PC) during the course of the control period (C_I-II), the clindamycin treatment period (CLI), the CDI stabilization period (CDI_I-III), the vancomycin treatment period (VNC) and the post-intervention period (PI_I-III) (n=1). The intensity of the shading indicates the absolute abundance, normalized for each of the different phyla (i.e., within each row).

Phylum	Family	PC																			
		AB										LAC10V0									
		C_I	C_II	CLI	CDI_I	CDI_II	CDI_III	VNC	PI_I	PI_II	PI_III	C_I	C_II	CLI	CDI_I	CDI_II	CDI_III	VNC	PI_I	PI_II	PI_III
**Actinobacteria**	***Bifidobacteriaceae***	8.13	7.86	<LOQ	<LOQ	7.40	7.47	5.95	<LOQ	7.70	6.85	8.15	7.96	<LOQ	<LOQ	8.13	7.59	5.76	<LOQ	9.01	8.68
	Microbacteriaceae	<LOQ	<LOQ	6.03	6.12	6.82	7.45	5.96	<LOQ	<LOQ	<LOQ	<LOQ	<LOQ	<LOQ	<LOQ	6.56	<LOQ	6.15	<LOQ	5.75	<LOQ
	Micrococcales_unclassified	<LOQ	<LOQ	<LOQ	<LOQ	<LOQ	<LOQ	<LOQ	<LOQ	<LOQ	<LOQ	<LOQ	<LOQ	6.03	6.38	5.95	<LOQ	6.81	<LOQ	5.87	5.85
**Bacteroidetes**	***Bacteroidaceae***	8.89	8.86	8.49	8.47	8.38	8.75	<LOQ	8.24	8.53	8.90	8.93	9.02	8.96	8.38	8.96	8.84	<LOQ	8.81	5.87	5.65
	Prevotellaceae	6.88	6.68	8.06	8.13	7.99	8.60	<LOQ	<LOQ	<LOQ	<LOQ	7.02	6.88	7.78	8.18	7.85	7.90	<LOQ	<LOQ	<LOQ	<LOQ
	Rikenellaceae	5.72	5.69	<LOQ	<LOQ	<LOQ	<LOQ	<LOQ	<LOQ	<LOQ	<LOQ	5.90	6.00	<LOQ	<LOQ	<LOQ	<LOQ	<LOQ	<LOQ	<LOQ	<LOQ
	Tannerellaceae	8.23	7.38	<LOQ	8.13	7.58	8.05	<LOQ	5.89	6.80	7.53	7.83	7.71	<LOQ	<LOQ	7.53	7.18	<LOQ	<LOQ	<LOQ	<LOQ
**Desulfobacteria**	***Desulfovibrionaceae***	6.72	6.68	<LOQ	7.31	7.24	7.35	6.79	7.42	6.58	7.31	7.05	6.93	<LOQ	7.78	7.39	7.46	6.30	<LOQ	6.11	<LOQ
**Firmicutes**	***Acidaminococcaceae***	7.32	6.94	<LOQ	<LOQ	<LOQ	<LOQ	<LOQ	<LOQ	<LOQ	<LOQ	7.32	7.37	<LOQ	<LOQ	<LOQ	<LOQ	<LOQ	<LOQ	<LOQ	<LOQ
	Carnobacteriaceae	6.20	5.75	5.70	<LOQ	6.65	6.49	5.99	<LOQ	<LOQ	5.78	5.97	5.78	6.34	5.97	5.98	5.88	6.07	<LOQ	6.75	6.03
	Clostridiaceae	<LOQ	<LOQ	<LOQ	<LOQ	<LOQ	6.70	<LOQ	<LOQ	<LOQ	<LOQ	<LOQ	<LOQ	<LOQ	<LOQ	6.10	<LOQ	<LOQ	<LOQ	<LOQ	<LOQ
	Enterococcaceae	5.96	<LOQ	5.91	6.59	5.81	8.39	<LOQ	<LOQ	<LOQ	6.18	6.38	<LOQ	5.75	6.08	7.06	5.92	5.82	<LOQ	5.87	<LOQ
	Lachnospiraceae	8.58	8.84	<LOQ	8.45	7.77	7.82	<LOQ	<LOQ	<LOQ	<LOQ	8.75	8.26	<LOQ	5.79	8.04	8.27	<LOQ	6.08	7.18	6.95
	Lactobacillaceae	<LOQ	<LOQ	<LOQ	5.62	<LOQ	6.10	6.30	<LOQ	<LOQ	6.27	<LOQ	<LOQ	<LOQ	6.67	<LOQ	<LOQ	6.59	6.33	7.33	7.80
	Ruminococcaceae	<LOQ	<LOQ	<LOQ	<LOQ	<LOQ	<LOQ	<LOQ	5.99	<LOQ	<LOQ	<LOQ	<LOQ	<LOQ	<LOQ	<LOQ	<LOQ	<LOQ	<LOQ	<LOQ	<LOQ
	Selenomonadaceae	6.16	5.69	<LOQ	<LOQ	<LOQ	<LOQ	<LOQ	<LOQ	<LOQ	<LOQ	<LOQ	<LOQ	<LOQ	<LOQ	<LOQ	<LOQ	<LOQ	<LOQ	<LOQ	<LOQ
	Veillonellaceae	7.44	7.24	<LOQ	<LOQ	<LOQ	<LOQ	<LOQ	<LOQ	<LOQ	<LOQ	7.58	7.92	<LOQ	<LOQ	<LOQ	<LOQ	<LOQ	6.40	8.78	8.45
**Proteobacteria**	***Alcaligenaceae***	5.72	<LOQ	5.85	6.31	<LOQ	7.99	<LOQ	<LOQ	<LOQ	<LOQ	6.20	<LOQ	6.05	6.12	7.75	6.16	<LOQ	<LOQ	5.75	<LOQ
	Enterobacterales_ unclassified	<LOQ	<LOQ	6.08	5.73	5.99	6.61	5.69	6.90	6.79	6.31	<LOQ	<LOQ	6.07	5.86	5.95	<LOQ	6.30	7.00	<LOQ	<LOQ
	Enterobacteriaceae	6.26	6.44	8.86	8.32	8.38	9.12	8.48	9.13	8.86	8.70	7.88	7.07	8.67	8.68	8.83	8.06	9.02	9.28	7.41	6.89
	Pseudomonadaceae	6.37	5.99	6.71	7.24	7.12	8.22	6.41	<LOQ	5.90	6.75	6.80	<LOQ	7.33	7.16	7.94	6.74	6.73	6.03	6.05	6.04
	Sutterellaceae	7.23	6.93	<LOQ	7.26	7.11	7.29	7.35	<LOQ	6.36	7.05	7.31	7.29	<LOQ	7.13	6.71	7.09	7.69	6.33	6.97	7.37
	uncultured	7.39	7.18	<LOQ	<LOQ	<LOQ	<LOQ	<LOQ	<LOQ	<LOQ	<LOQ	7.20	6.78	<LOQ	<LOQ	<LOQ	<LOQ	<LOQ	<LOQ	<LOQ	<LOQ
	Xanthomonadaceae	6.02	6.11	6.24	6.92	5.89	8.19	6.51	<LOQ	<LOQ	<LOQ	6.16	<LOQ	5.86	6.20	7.94	6.90	6.06	<LOQ	<LOQ	<LOQ

Table S7. Proximal microbial community composition at family level for the AB and LAC10V1 arms. Abundance of different families (log cells/mL) in arm 2 (AB) and arm 8 (LAC10V1) of the PathoGut™ SHIME experiment in the proximal colon (PC) during the course of the control period (C_I-II), the clindamycin treatment period (CLI), the CDI stabilization period (CDI_I-III), the vancomycin treatment period (VNC) and the post-intervention period (PI_I-III) (n=1). The intensity of the shading indicates the absolute abundance, normalized for each of the different phyla (i.e., within each row).

Phylum	Family	PC																			
		AB										LAC10V1									
		C_I	C_II	CLI	CDI_I	CDI_II	CDI_III	VNC	PI_I	PI_II	PI_III	C_I	C_II	CLI	CDI_I	CDI_II	CDI_III	VNC	PI_I	PI_II	PI_III
Actinobacteria	*Bifidobacteriaceae*	8.13	7.86	<LOQ	<LOQ	7.40	7.47	5.95	<LOQ	7.70	6.85	7.93	7.84	<LOQ	<LOQ	7.18	8.18	5.79	<LOQ	8.95	9.03
	Microbacteriaceae	<LOQ	<LOQ	6.03	6.12	6.82	7.45	5.96	<LOQ	<LOQ	<LOQ	<LOQ	<LOQ	<LOQ	<LOQ	5.84	6.07	6.50	6.17	<LOQ	<LOQ
	Micrococcales_un-classified	<LOQ	<LOQ	<LOQ	<LOQ	<LOQ	<LOQ	<LOQ	<LOQ	<LOQ	<LOQ	<LOQ	<LOQ	5.67	6.00	5.99	6.09	7.28	5.69	<LOQ	<LOQ
Bacteroidetes	*Bacteroidaceae*	8.89	8.86	8.49	8.47	8.38	8.75	<LOQ	8.24	8.53	8.90	8.91	8.98	8.60	8.34	8.19	8.47	<LOQ	7.89	7.18	8.16
	Prevotellaceae	6.88	6.68	8.06	8.13	7.99	8.60	<LOQ	<LOQ	<LOQ	<LOQ	7.36	7.22	8.08	7.88	7.75	7.78	<LOQ	<LOQ	<LOQ	<LOQ
	Rikenellaceae	5.72	5.69	<LOQ	<LOQ	<LOQ	<LOQ	<LOQ	<LOQ	<LOQ	<LOQ	<LOQ	5.78	<LOQ	<LOQ	<LOQ	<LOQ	<LOQ	<LOQ	<LOQ	<LOQ
	Tannerellaceae	8.23	7.38	<LOQ	8.13	7.58	8.05	<LOQ	5.89	6.80	7.53	7.88	8.22	<LOQ	8.03	7.54	7.85	<LOQ	<LOQ	<LOQ	<LOQ
Desulfobacteria	*Desulfovibrionaceae*	6.72	6.68	<LOQ	7.31	7.24	7.35	6.79	7.42	6.58	7.31	6.95	7.23	<LOQ	7.40	7.39	7.37	<LOQ	<LOQ	<LOQ	6.37
Firmicutes	*Acidaminococcaceae*	7.32	6.94	<LOQ	<LOQ	<LOQ	<LOQ	<LOQ	<LOQ	<LOQ	<LOQ	7.05	7.36	<LOQ	<LOQ	<LOQ	<LOQ	<LOQ	<LOQ	<LOQ	<LOQ
	Carnobacteriaceae	6.20	5.75	5.70	<LOQ	6.65	6.49	5.99	<LOQ	<LOQ	5.78	<LOQ	5.78	<LOQ	<LOQ	<LOQ	<LOQ	5.68	<LOQ	<LOQ	<LOQ
	Clostridiaceae	<LOQ	<LOQ	<LOQ	<LOQ	<LOQ	6.70	<LOQ	<LOQ	<LOQ	<LOQ	<LOQ	<LOQ	<LOQ	<LOQ	<LOQ	<LOQ	<LOQ	<LOQ	<LOQ	<LOQ
	Enterococcaceae	5.96	<LOQ	5.91	6.59	5.81	8.39	<LOQ	<LOQ	<LOQ	6.18	<LOQ	<LOQ	<LOQ	<LOQ	<LOQ	6.32	<LOQ	5.69	<LOQ	<LOQ
	Lachnospiraceae	8.58	8.84	<LOQ	8.45	7.77	7.82	<LOQ	<LOQ	<LOQ	<LOQ	8.63	8.63	<LOQ	8.60	8.80	8.65	<LOQ	5.99	8.30	7.83
	Lactobacillaceae	<LOQ	<LOQ	<LOQ	5.62	<LOQ	6.10	6.30	<LOQ	<LOQ	6.27	<LOQ	<LOQ	<LOQ	6.56	<LOQ	<LOQ	5.82	5.69	6.48	6.58
	Ruminococcaceae	<LOQ	<LOQ	<LOQ	<LOQ	<LOQ	<LOQ	<LOQ	5.99	<LOQ	<LOQ	<LOQ	<LOQ	<LOQ	<LOQ	<LOQ	<LOQ	5.79	<LOQ	<LOQ	<LOQ
	Selenomonadaceae	6.16	5.69	<LOQ	<LOQ	<LOQ	<LOQ	<LOQ	<LOQ	<LOQ	<LOQ	<LOQ	<LOQ	<LOQ	<LOQ	<LOQ	<LOQ	<LOQ	<LOQ	<LOQ	<LOQ
	Veillonellaceae	7.44	7.24	<LOQ	<LOQ	<LOQ	<LOQ	<LOQ	<LOQ	<LOQ	<LOQ	7.38	7.92	<LOQ	<LOQ	<LOQ	8.36	7.53	8.10	8.46	8.78
Proteobacteria	*Alcaligenaceae*	5.72	<LOQ	5.85	6.31	<LOQ	7.99	<LOQ	<LOQ	<LOQ	<LOQ	5.83	5.70	6.13	5.62	5.90	6.92	6.65	<LOQ	5.66	<LOQ
	Enterobacterales _unclassified	<LOQ	<LOQ	6.08	5.73	5.99	6.61	5.69	6.90	6.79	6.31	<LOQ	<LOQ	5.94	<LOQ	<LOQ	<LOQ	6.06	6.67	<LOQ	<LOQ
	Enterobacteriaceae	6.26	6.44	8.86	8.32	8.38	9.12	8.48	9.13	8.86	8.70	7.92	7.93	8.86	8.10	7.98	8.55	8.85	9.21	7.29	7.46
	Pseudomonadaceae	6.37	5.99	6.71	7.24	7.12	8.22	6.41	<LOQ	5.90	6.75	6.27	6.58	6.98	6.22	5.90	6.46	6.66	6.29	<LOQ	5.80
	Sutterellaceae	7.23	6.93	<LOQ	7.26	7.11	7.29	7.35	<LOQ	6.36	7.05	6.98	7.32	<LOQ	<LOQ	6.04	6.38	5.94	5.87	<LOQ	5.71
	uncultured	7.39	7.18	<LOQ	<LOQ	<LOQ	<LOQ	<LOQ	<LOQ	<LOQ	<LOQ	7.00	7.09	<LOQ	<LOQ	<LOQ	<LOQ	<LOQ	<LOQ	<LOQ	<LOQ
	Xanthomonadaceae	6.02	6.11	6.24	6.92	5.89	8.19	6.51	<LOQ	<LOQ	<LOQ	6.21	6.04	6.52	6.33	6.43	7.16	6.25	5.87	5.61	<LOQ

Table S8. Distal microbial community composition at family level for the AB and LAC10V0 arms. Abundance of different families (log cells/mL) in arm 2 (AB) and arm 6 (LAC10V0) of the PathoGut™ SHIME experiment in the distal colon (DC) during the course of the control period (C_I-II), the clindamycin treatment period (CLI), the CDI stabilization period (CDI_I-III), the vancomycin treatment period (VNC) and the post-intervention period (PI_I-III) (n=1). The intensity of the shading indicates the absolute abundance, normalized for each of the different phyla (i.e., within each row).

Phylum	Family	DC																			
		AB										LAC10V0									
		C_I	C_II	CLI	CDI_I	CDI_II	CDI_III	VNC	PI_I	PI_II	PI_III	C_I	C_II	CLI	CDI_I	CDI_II	CDI_III	VNC	PI_I	PI_II	PI_III
Actino-bacteria	*Bifidobacteriaceae*	8.36	8.41	<LOQ	<LOQ	6.91	7.19	5.93	<LOQ	8.03	7.28	8.39	8.18	<LOQ	<LOQ	8.35	7.94	<LOQ	<LOQ	8.90	8.29
	Coriobacteriaceae	<LOQ	5.80	<LOQ	<LOQ	<LOQ	<LOQ	<LOQ	<LOQ	<LOQ	<LOQ	5.78	5.87	<LOQ	<LOQ	<LOQ	<LOQ	<LOQ	<LOQ	<LOQ	<LOQ
	Microbacteriaceae	<LOQ	5.68	6.05	6.46	6.89	6.75	5.74	<LOQ	<LOQ	<LOQ	<LOQ	<LOQ	<LOQ	<LOQ	6.32	5.78	5.81	5.69	<LOQ	<LOQ
	Micrococcales_unclas-sified	<LOQ	<LOQ	<LOQ	<LOQ	<LOQ	<LOQ	<LOQ	<LOQ	<LOQ	5.95	<LOQ	<LOQ	6.06	6.57	6.70	6.28	5.86	<LOQ	6.28	5.81
Bac-teroide-tes	*Bacteroidaceae*	8.73	8.84	8.49	8.90	8.82	8.77	<LOQ	7.63	8.88	9.06	8.69	8.90	8.85	8.65	8.91	9.02	<LOQ	8.44	9.05	9.19
	Marinifilaceae	<LOQ	<LOQ	<LOQ	<LOQ	<LOQ	5.64	<LOQ	<LOQ	<LOQ	<LOQ	<LOQ	<LOQ	<LOQ	<LOQ	5.74	<LOQ	<LOQ	<LOQ	6.11	6.51
	Muribaculaceae	6.43	6.05	<LOQ	<LOQ	<LOQ	<LOQ	<LOQ	<LOQ	<LOQ	<LOQ	<LOQ	<LOQ	<LOQ	<LOQ	<LOQ	<LOQ	<LOQ	<LOQ	<LOQ	<LOQ
	Prevotellaceae	6.06	6.58	6.53	7.24	6.50	6.54	<LOQ	<LOQ	<LOQ	<LOQ	6.39	<LOQ	6.45	6.39	6.91	7.10	<LOQ	<LOQ	<LOQ	<LOQ
	Rikenellaceae	6.32	6.38	<LOQ	7.44	6.85	6.64	<LOQ	<LOQ	<LOQ	<LOQ	6.04	6.25	<LOQ	6.25	7.09	6.71	<LOQ	<LOQ	6.35	6.58
	Tannerellaceae	7.67	7.48	<LOQ	7.94	7.50	7.84	<LOQ	5.91	6.93	7.81	7.38	7.41	<LOQ	7.44	7.79	7.90	<LOQ	7.25	7.37	7.57
Desul-fobacte-ria	*Desulfovibrionaceae*	6.96	7.15	<LOQ	7.70	7.67	7.43	7.79	7.54	7.14	7.70	7.13	7.12	<LOQ	8.10	7.72	7.76	7.74	6.90	6.94	7.59
Firmicu-tes	*Acidaminococcaceae*	6.89	7.01	<LOQ	<LOQ	<LOQ	<LOQ	<LOQ	<LOQ	<LOQ	<LOQ	7.03	6.42	<LOQ	<LOQ	<LOQ	<LOQ	<LOQ	<LOQ	<LOQ	<LOQ
	Anaerovoracaceae	6.46	5.90	<LOQ	6.70	6.29	6.26	<LOQ	<LOQ	6.36	6.16	6.20	6.01	<LOQ	6.87	6.67	6.49	<LOQ	5.87	5.98	<LOQ
	Butyricicoccaceae	<LOQ	5.98	6.30	5.73	5.82	5.98	<LOQ	<LOQ	6.17	5.64	5.99	5.87	<LOQ	6.37	6.14	<LOQ	5.88	6.88	6.20	<LOQ
	Carnobacteriaceae	<LOQ	5.98	6.08	<LOQ	6.12	6.14	6.11	<LOQ	<LOQ	5.89	<LOQ	5.77	6.18	<LOQ	<LOQ	<LOQ	<LOQ	<LOQ	<LOQ	<LOQ
	Clostridiaceae	<LOQ	<LOQ	<LOQ	6.55	5.73	6.14	<LOQ	<LOQ	7.14	6.97	<LOQ	<LOQ	<LOQ	6.40	7.11	5.68	<LOQ	<LOQ	6.20	<LOQ
	Enterococcaceae	5.88	5.80	6.15	6.43	5.91	7.01	<LOQ	<LOQ	5.61	6.00	5.86	<LOQ	6.19	5.91	6.92	6.12	5.64	<LOQ	<LOQ	<LOQ
	Erysipelotrichaceae	<LOQ	<LOQ	<LOQ	5.64	<LOQ	5.89	<LOQ	<LOQ	5.61	6.34	<LOQ	5.71	<LOQ	<LOQ	5.84	<LOQ	<LOQ	<LOQ	6.20	<LOQ
	Eubacteriaceae	6.23	6.28	<LOQ	<LOQ	6.54	6.02	<LOQ	<LOQ	6.13	6.91	5.78	5.82	<LOQ	6.80	6.67	6.40	<LOQ	<LOQ	8.02	8.03
	Lachnospiraceae	8.79	8.83	6.27	8.63	8.65	8.72	<LOQ	5.62	7.40	7.91	8.79	8.35	6.36	8.59	8.76	8.76	<LOQ	<LOQ	8.93	8.42
	Lactobacillaceae	<LOQ	<LOQ	<LOQ	<LOQ	<LOQ	5.89	6.33	<LOQ	<LOQ	6.08	<LOQ	<LOQ	5.68	6.76	5.92	6.08	6.05	6.57	7.01	7.55
	Oscillospiraceae	6.98	6.91	7.13	6.98	6.86	6.72	<LOQ	<LOQ	6.56	6.87	6.94	6.68	7.28	7.45	7.35	7.09	<LOQ	<LOQ	7.10	6.17
	Oscillospirales_fa	6.28	6.38	<LOQ	6.01	5.79	<LOQ	<LOQ	<LOQ	<LOQ	<LOQ	<LOQ	<LOQ	<LOQ	<LOQ	<LOQ	<LOQ	<LOQ	<LOQ	<LOQ	<LOQ
	Ruminococcaceae	6.56	6.35	6.09	7.18	6.75	7.17	<LOQ	<LOQ	6.62	7.37	6.39	6.04	<LOQ	7.51	7.64	7.47	<LOQ	<LOQ	6.55	<LOQ

	Veillonellaceae	6.60	6.54	<LOQ	<LOQ	<LOQ	7.68	<LOQ	<LOQ	<LOQ	<LOQ	7.10	7.12	<LOQ	<LOQ	<LOQ	<LOQ	<LOQ	8.10	8.64	8.56
**Proteo-bacteria**	***Alcaligenaceae***	<LOQ	6.24	6.02	6.48	5.73	7.14	<LOQ	<LOQ	5.73	6.22	5.69	<LOQ	6.03	6.34	7.32	7.13	<LOQ	<LOQ	<LOQ	<LOQ
	Enterobacterales_ un-classified	<LOQ	<LOQ	5.72	<LOQ	<LOQ	6.12	<LOQ	6.72	6.49	6.34	<LOQ	<LOQ	6.21	<LOQ	<LOQ	<LOQ	6.51	7.26	<LOQ	<LOQ
	Enterobacteriaceae	<LOQ	5.90	8.79	8.12	7.81	8.49	8.49	9.09	8.63	8.58	7.04	5.82	8.78	8.37	8.15	7.97	9.36	9.43	7.23	7.29
	Pseudomonadaceae	7.06	7.17	7.35	7.88	7.61	7.99	6.70	<LOQ	6.49	7.27	7.03	7.32	7.70	7.85	7.95	7.46	5.79	6.39	6.58	6.83
	Sutterellaceae	7.11	7.07	<LOQ	7.62	7.57	7.39	7.27	5.96	6.76	7.46	7.22	7.28	<LOQ	7.37	7.48	7.72	7.67	6.67	6.35	7.35
	uncultured	7.11	7.13	<LOQ	7.13	7.08	7.87	<LOQ	<LOQ	<LOQ	<LOQ	6.79	6.15	<LOQ	7.67	6.64	6.75	<LOQ	<LOQ	<LOQ	<LOQ
	Xanthomonadaceae	<LOQ	<LOQ	6.18	6.47	6.05	7.19	5.74	<LOQ	5.61	5.95	<LOQ	5.65	<LOQ	6.37	7.29	7.01	<LOQ	<LOQ	<LOQ	<LOQ
**Verru-comicro-bia**	***Akkermansiaceae***	6.74	7.17	<LOQ	<LOQ	<LOQ	7.97	<LOQ	<LOQ	<LOQ	<LOQ	8.32	8.28	<LOQ	<LOQ	<LOQ	<LOQ	<LOQ	<LOQ	<LOQ	<LOQ
	Victivallaceae	<LOQ	<LOQ	<LOQ	<LOQ	<LOQ	6.96	<LOQ	<LOQ	6.43	7.06	<LOQ	<LOQ	<LOQ	6.86	5.92	5.98	<LOQ	<LOQ	6.20	5.96

Table S9. Distal microbial community composition at family level for the AB and LAC10V1 arms. Abundance of different families (log cells/mL) in arm 2 (AB) and arm 8 (LAC10V1) of the PathoGut™ SHIME experiment in the distal colon (DC) during the course of the control period (C_I-II), the clindamycin treatment period (CLI), the CDI stabilization period (CDI_I-III), the vancomycin treatment period (VNC) and the post-intervention period (PI_I-III) (n=1). The intensity of the shading indicates the absolute abundance, normalized for each of the different phyla (i.e., within each row).

Phylum	Family	DC																			
		AB										LAC10V1									
		C_I	C_II	CLI	CDI_I	CDI_II	CDI_III	VNC	PI_I	PI_II	PI_III	C_I	C_II	CLI	CDI_I	CDI_II	CDI_III	VNC	PI_I	PI_II	PI_III
Actinobacteria	*Bifidobacteriaceae*	8.36	8.41	<LOQ	<LOQ	6.91	7.19	5.93	<LOQ	8.03	7.28	8.53	8.34	<LOQ	<LOQ	7.54	8.58	<LOQ	<LOQ	8.86	8.88
	Coriobacteriaceae	<LOQ	5.80	<LOQ	<LOQ	<LOQ	<LOQ	<LOQ	<LOQ	<LOQ	<LOQ	5.69	5.86	<LOQ	<LOQ	<LOQ	<LOQ	<LOQ	<LOQ	<LOQ	<LOQ
	Microbacteriaceae	<LOQ	5.68	6.05	6.46	6.89	6.75	5.74	<LOQ	<LOQ	<LOQ	<LOQ	<LOQ	<LOQ	<LOQ	5.76	5.98	<LOQ	<LOQ	<LOQ	<LOQ
	Micrococcales_unclassified	<LOQ	<LOQ	<LOQ	<LOQ	<LOQ	<LOQ	<LOQ	<LOQ	<LOQ	5.95	<LOQ	<LOQ	5.63	6.25	6.39	6.39	5.65	<LOQ	6.01	5.97
Bacteroidetes	*Bacteroidaceae*	8.73	8.84	8.49	8.90	8.82	8.77	<LOQ	7.63	8.88	9.06	8.92	8.87	8.70	8.53	8.61	8.51	<LOQ	7.78	8.69	8.77
	Marinifilaceae	<LOQ	<LOQ	<LOQ	<LOQ	<LOQ	5.64	<LOQ	<LOQ	<LOQ	<LOQ	<LOQ	<LOQ	<LOQ	<LOQ	5.80	<LOQ	<LOQ	<LOQ	6.51	6.47
	Muribaculaceae	6.43	6.05	<LOQ	<LOQ	<LOQ	<LOQ	<LOQ	<LOQ	<LOQ	<LOQ	5.87	5.86	<LOQ	<LOQ	<LOQ	<LOQ	<LOQ	<LOQ	<LOQ	<LOQ
	Prevotellaceae	6.06	6.58	6.53	7.24	6.50	6.54	<LOQ	<LOQ	<LOQ	<LOQ	<LOQ	<LOQ	6.66	6.63	7.15	6.83	<LOQ	<LOQ	<LOQ	<LOQ
	Rikenellaceae	6.32	6.38	<LOQ	7.44	6.85	6.64	<LOQ	<LOQ	<LOQ	<LOQ	6.32	6.52	<LOQ	7.06	6.49	6.43	<LOQ	<LOQ	7.80	7.32
	Tannerellaceae	7.67	7.48	<LOQ	7.94	7.50	7.84	<LOQ	5.91	6.93	7.81	7.54	7.80	6.65	7.26	7.69	7.59	<LOQ	7.90	7.98	8.18
Desulfobacteria	*Desulfovibrionaceae*	6.96	7.15	<LOQ	7.70	7.67	7.43	7.79	7.54	7.14	7.70	7.19	7.44	<LOQ	7.75	7.54	7.38	6.73	6.61	7.26	7.32
Firmicutes	*Acidaminococcaceae*	6.89	7.01	<LOQ	<LOQ	<LOQ	<LOQ	<LOQ	<LOQ	<LOQ	<LOQ	6.78	6.93	<LOQ	<LOQ	<LOQ	<LOQ	<LOQ	<LOQ	<LOQ	<LOQ
	Anaerovoracaceae	6.46	5.90	<LOQ	6.70	6.29	6.26	<LOQ	<LOQ	6.36	6.16	6.39	6.16	<LOQ	6.60	6.31	6.21	<LOQ	<LOQ	<LOQ	<LOQ
	Butyricicoccaceae	<LOQ	5.98	6.30	5.73	5.82	5.98	<LOQ	<LOQ	6.17	5.64	<LOQ	6.12	<LOQ	5.61	5.67	5.74	<LOQ	<LOQ	5.98	5.85
	Carnobacteriaceae	<LOQ	5.98	6.08	<LOQ	6.12	6.14	6.11	<LOQ	<LOQ	5.89	6.13	6.06	<LOQ	<LOQ	<LOQ	5.61	<LOQ	<LOQ	<LOQ	<LOQ
	Clostridiaceae	<LOQ	<LOQ	<LOQ	6.55	5.73	6.14	<LOQ	<LOQ	7.14	6.97	<LOQ	<LOQ	<LOQ	<LOQ	<LOQ	5.61	<LOQ	<LOQ	<LOQ	<LOQ
	Enterococcaceae	5.88	5.80	6.15	6.43	5.91	7.01	<LOQ	<LOQ	5.61	6.00	<LOQ	<LOQ	<LOQ	5.61	5.76	6.31	<LOQ	<LOQ	6.01	5.85
	Erysipelotrichaceae	<LOQ	<LOQ	<LOQ	5.64	<LOQ	5.89	<LOQ	<LOQ	5.61	6.34	<LOQ	5.64	<LOQ	<LOQ	<LOQ	5.83	<LOQ	<LOQ	<LOQ	5.93
	Eubacteriaceae	6.23	6.28	<LOQ	<LOQ	6.54	6.02	<LOQ	<LOQ	6.13	6.91	6.17	6.20	<LOQ	5.65	6.18	6.31	<LOQ	<LOQ	7.07	7.52
	Lachnospiraceae	8.79	8.83	6.27	8.63	8.65	8.72	<LOQ	5.62	7.40	7.91	8.62	8.77	6.22	8.59	8.94	8.95	<LOQ	<LOQ	8.42	8.14
	Lactobacillaceae	<LOQ	<LOQ	<LOQ	<LOQ	<LOQ	5.89	6.33	<LOQ	<LOQ	6.08	<LOQ	<LOQ	5.94	6.69	<LOQ	<LOQ	<LOQ	<LOQ	6.51	6.53
	Oscillospiraceae	6.98	6.91	7.13	6.98	6.86	6.72	<LOQ	<LOQ	6.56	6.87	6.95	7.19	7.43	6.97	6.95	6.81	<LOQ	<LOQ	6.54	6.87

	Oscillospirales_fa	6.28	6.38	<LOQ	6.01	5.79	<LOQ	<LOQ	<LOQ	<LOQ	<LOQ	6.59	5.94	<LOQ	<LOQ	<LOQ	<LOQ	<LOQ	<LOQ	<LOQ	<LOQ
	Ruminococcaceae	6.56	6.35	6.09	7.18	6.75	7.17	<LOQ	<LOQ	6.62	7.37	6.66	6.68	5.66	7.61	7.31	6.93	<LOQ	<LOQ	<LOQ	6.03
	Veillonellaceae	6.60	6.54	<LOQ	<LOQ	<LOQ	7.68	<LOQ	<LOQ	<LOQ	<LOQ	6.32	7.40	<LOQ	<LOQ	<LOQ	7.71	<LOQ	7.28	8.70	8.77
**Proteobacteria**	*Alcaligenaceae*	<LOQ	6.24	6.02	6.48	5.73	7.14	<LOQ	<LOQ	5.73	6.22	<LOQ	5.94	5.72	5.80	6.46	6.64	<LOQ	<LOQ	6.04	6.00
	Enterobacterales_ unclassified	<LOQ	<LOQ	5.72	<LOQ	<LOQ	6.12	<LOQ	6.72	6.49	6.34	<LOQ	<LOQ	5.93	<LOQ	<LOQ	<LOQ	6.94	7.34	<LOQ	<LOQ
	Enterobacteriaceae	<LOQ	5.90	8.79	8.12	7.81	8.49	8.49	9.09	8.63	8.58	6.62	7.01	8.89	7.79	7.70	8.02	9.28	9.49	7.36	7.21
	Pseudomonadaceae	7.06	7.17	7.35	7.88	7.61	7.99	6.70	<LOQ	6.49	7.27	7.03	7.50	7.57	7.70	7.69	7.46	<LOQ	<LOQ	5.79	6.73
	Sutterellaceae	7.11	7.07	<LOQ	7.62	7.57	7.39	7.27	5.96	6.76	7.46	6.93	7.38	<LOQ	7.15	6.69	6.61	6.19	<LOQ	7.40	7.42
	uncultured	7.11	7.13	<LOQ	7.13	7.08	7.87	<LOQ	<LOQ	<LOQ	<LOQ	6.74	6.82	<LOQ	<LOQ	6.47	<LOQ	<LOQ	<LOQ	<LOQ	<LOQ
	Xanthomonadaceae	<LOQ	<LOQ	6.18	6.47	6.05	7.19	5.74	<LOQ	5.61	5.95	<LOQ	6.01	5.77	5.73	6.35	6.53	<LOQ	<LOQ	<LOQ	<LOQ
**Verrucomicrobia**	*Akkermansiaceae*	6.74	7.17	<LOQ	<LOQ	<LOQ	7.97	<LOQ	<LOQ	<LOQ	<LOQ	6.98	7.90	<LOQ	<LOQ	<LOQ	<LOQ	<LOQ	<LOQ	<LOQ	<LOQ
	Victivallaceae	<LOQ	<LOQ	<LOQ	<LOQ	<LOQ	6.96	<LOQ	<LOQ	6.43	7.06	<LOQ	<LOQ	<LOQ	6.03	6.06	<LOQ	<LOQ	<LOQ	<LOQ	6.47

Table S10. Proximal microbial community composition at OTU level for the LAC5V0, LAC10V0, LAC5V1 and LAC10V1 arms. Abundance of top 25 most abundant OTUs (log cells/mL) in arm 5 (LAC5V0), arm 6 (LAC10V0), arm 7 (LAC5V1) and arm 8 (LAC10V1) of the PathoGut™ SHIME experiment in the proximal colon (PC) during the course of the control period (C_I-II), the clindamycin treatment period (CLI), the CDI stabilization period (CDI_I-III), the vancomycin treatment period (VNC) and the post-intervention period (PI_I-III) (n=1). The intensity of the shading indicates the absolute abundance, normalized for each of the different phyla (i.e., within each row).

OTU	Closely related species	PC																			
		LAC5V0										LAC10V0									
		C_I	C_II	CLI	CDI_I	CDI_II	CDI_III	VNC	PI_I	PI_II	PI_III	C_I	C_II	CLI	CDI_I	CDI_II	CDI_III	VNC	PI_I	PI_II	PI_III
**Otu00005**	*Bifidobacterium adolescentis*	8.01	7.73	<LOQ	<LOQ	<LOQ	<LOQ	<LOQ	<LOQ	<LOQ	<LOQ	8.12	7.91	<LOQ	<LOQ	<LOQ	<LOQ	<LOQ	<LOQ	5.87	<LOQ
**Otu00014**	*Bifidobacterium longum*	6.29	6.93	<LOQ	6.73	7.43	6.91	<LOQ	<LOQ	8.14	8.34	6.46	6.57	<LOQ	<LOQ	8.07	7.51	5.71	<LOQ	8.95	8.62
**Otu00013**	*Bacteroides fragilis*	7.45	7.83	<LOQ	<LOQ	<LOQ	<LOQ	<LOQ	<LOQ	<LOQ	<LOQ	7.69	8.56	<LOQ	<LOQ	<LOQ	<LOQ	<LOQ	<LOQ	<LOQ	<LOQ
**Otu00003**	*Bacteroides thetaiotaomi-cron*	8.52	8.57	8.96	7.79	7.96	7.27	<LOQ	7.91	6.71	6.92	8.55	8.34	8.87	7.53	8.10	7.83	<LOQ	8.71	<LOQ	<LOQ
**Otu00004**	*Bacteroides ovatus*	8.35	8.49	7.92	8.20	7.87	7.96	<LOQ	7.65	8.78	7.99	8.40	8.50	8.01	7.88	8.86	8.76	<LOQ	8.05	<LOQ	<LOQ
**Otu00006**	*Bacteroides dorei*	7.47	7.58	7.12	7.89	8.45	8.61	<LOQ	8.11	<LOQ	<LOQ	7.82	7.59	7.57	8.10	7.63	7.49	<LOQ	6.46	<LOQ	<LOQ
**Otu00025**	*Bacteroides finegoldii*	<LOQ	<LOQ	<LOQ	<LOQ	<LOQ	<LOQ	<LOQ	<LOQ	<LOQ	<LOQ	<LOQ	<LOQ	<LOQ	<LOQ	<LOQ	<LOQ	<LOQ	<LOQ	<LOQ	<LOQ
**Otu00020**	*Bacteroides salyersiae*	7.11	7.19	7.79	6.29	6.90	5.80	<LOQ	<LOQ	6.89	6.24	6.69	6.52	<LOQ	<LOQ	<LOQ	<LOQ	<LOQ	<LOQ	<LOQ	<LOQ
**Otu00012**	*Paraprevotella clara*	7.01	6.70	8.23	7.55	7.82	7.78	<LOQ	<LOQ	<LOQ	<LOQ	6.88	6.76	7.62	8.05	7.71	7.75	<LOQ	<LOQ	<LOQ	<LOQ
**Otu00017**	*Paraprevotella clara*	6.65	6.41	7.79	7.09	7.38	7.39	<LOQ	<LOQ	<LOQ	<LOQ	6.42	6.28	7.23	7.59	7.29	7.36	<LOQ	<LOQ	<LOQ	<LOQ
**Otu00030**	*Alistipes finegoldii/onder-donkii*	6.13	6.53	<LOQ	<LOQ	<LOQ	<LOQ	<LOQ	<LOQ	<LOQ	<LOQ	<LOQ	<LOQ	<LOQ	<LOQ	<LOQ	<LOQ	<LOQ	<LOQ	<LOQ	<LOQ
**Otu00015**	*Parabacteroides distasonis*	7.22	7.11	5.66	6.91	6.81	7.50	<LOQ	7.40	6.48	7.27	7.58	7.03	<LOQ	<LOQ	7.53	7.18	<LOQ	<LOQ	<LOQ	<LOQ
**Otu00016**	*Bilophila wadsworthia*	7.15	7.21	<LOQ	7.37	7.40	7.31	<LOQ	7.12	6.26	6.43	7.05	6.93	<LOQ	7.78	7.39	7.46	6.29	<LOQ	5.97	<LOQ
**Otu00001**	*Clostridium clos-tridioforme/bolteae*	8.37	8.23	5.76	8.76	7.98	8.73	6.11	<LOQ	8.88	8.06	8.64	8.08	<LOQ	5.71	8.02	8.25	<LOQ	<LOQ	7.16	6.94
**Otu00022**	*Clostridium aldenense*	<LOQ	<LOQ	<LOQ	<LOQ	<LOQ	6.47	<LOQ	<LOQ	<LOQ	<LOQ	5.82	<LOQ	<LOQ	<LOQ	6.55	6.82	<LOQ	<LOQ	<LOQ	<LOQ
**Otu00027**	*Hungatella hathewayi*	<LOQ	<LOQ	5.84	6.16	5.81	5.92	6.09	<LOQ	6.05	5.64	<LOQ	<LOQ	<LOQ	<LOQ	<LOQ	<LOQ	<LOQ	<LOQ	<LOQ	<LOQ
**Otu00031**	*Faecalibacterium prausnitzii*	<LOQ	<LOQ	<LOQ	<LOQ	<LOQ	<LOQ	<LOQ	<LOQ	<LOQ	<LOQ	<LOQ	<LOQ	<LOQ	<LOQ	<LOQ	<LOQ	<LOQ	<LOQ	<LOQ	<LOQ
**Otu00032**	*Selenomonas infelix*	<LOQ	<LOQ	<LOQ	<LOQ	<LOQ	<LOQ	<LOQ	<LOQ	<LOQ	<LOQ	<LOQ	<LOQ	<LOQ	<LOQ	<LOQ	<LOQ	<LOQ	<LOQ	<LOQ	<LOQ
**Otu00007**	*Veillonella dispar*	5.79	5.72	<LOQ	7.51	7.69	7.53	9.04	8.57	8.08	7.78	<LOQ	<LOQ	<LOQ	<LOQ	<LOQ	<LOQ	<LOQ	6.35	8.77	8.44
**Otu00028**	*Megasphaera sp.*	<LOQ	6.86	<LOQ	7.23	5.89	5.80	<LOQ	8.02	7.35	7.66	6.12	6.88	<LOQ	<LOQ	<LOQ	<LOQ	<LOQ	<LOQ	<LOQ	<LOQ
**Otu00002**	*Klebsiella pneumoniae*	7.02	5.96	7.91	6.44	7.20	6.49	7.20	8.60	6.47	6.37	7.49	5.84	7.77	8.32	7.82	7.16	9.00	9.24	6.76	6.29

**Otu00011**	***Klebsiella oxytoca***	7.29	6.53	7.96	6.79	6.65	6.77	8.91	8.25	6.79	7.16	7.43	5.78	7.50	7.42	8.42	7.60	7.59	8.04	7.22	6.58
**Otu00008**	***Citrobacter freundii***	5.71	<LOQ	7.31	7.10	6.65	6.28	7.08	6.72	<LOQ	5.68	5.72	<LOQ	<LOQ	5.96	6.96	6.12	6.27	6.76	6.05	<LOQ
**Otu00009**	***Escherichia coli/Shigella sonnei***	6.56	6.88	8.58	7.60	8.00	8.20	7.81	8.65	7.41	7.30	7.21	7.00	8.56	8.37	8.50	7.75	6.64	6.98	6.31	6.18
**Otu00010**	***Akkermansia muciniphila***	<LOQ	<LOQ	<LOQ	<LOQ	<LOQ	<LOQ	<LOQ	6.24	<LOQ	<LOQ	<LOQ	<LOQ	<LOQ	<LOQ	<LOQ	<LOQ	<LOQ	<LOQ	<LOQ	<LOQ

**OTU**	**Closely related species**	**PC**																			
		**LAC5V1**										**LAC10V1**									
		**C_I**	**C_II**	**CLI**	**CDI_I**	**CDI_II**	**CDI_III**	**VNC**	**PI_I**	**PI_II**	**PI_III**	**C_I**	**C_II**	**CLI**	**CDI_I**	**CDI_II**	**CDI_III**	**VNC**	**PI_I**	**PI_II**	**PI_III**
**Otu00005**	***Bifidobacterium adolescentis***	8.18	8.01	<LOQ	<LOQ	<LOQ	<LOQ	<LOQ	<LOQ	<LOQ	<LOQ	7.92	7.80	<LOQ	<LOQ	7.18	8.18	5.79	<LOQ	8.94	9.03
**Otu00014**	***Bifidobacterium longum***	6.04	6.48	<LOQ	<LOQ	7.83	7.77	<LOQ	<LOQ	7.68	7.91	5.83	6.40	<LOQ	<LOQ	<LOQ	<LOQ	<LOQ	<LOQ	<LOQ	<LOQ
**Otu00013**	***Bacteroides fragilis***	7.45	8.01	<LOQ	<LOQ	<LOQ	<LOQ	<LOQ	<LOQ	<LOQ	<LOQ	7.57	7.38	<LOQ	<LOQ	<LOQ	<LOQ	<LOQ	<LOQ	<LOQ	<LOQ
**Otu00003**	***Bacteroides thetaiotaomi-cron***	8.70	8.36	8.87	8.78	7.99	7.62	<LOQ	9.07	6.62	7.76	8.32	8.49	8.57	6.86	7.79	7.56	<LOQ	7.87	7.18	8.16
**Otu00004**	***Bacteroides ovatus***	8.57	8.67	<LOQ	<LOQ	7.91	7.96	<LOQ	<LOQ	<LOQ	<LOQ	8.60	8.66	<LOQ	<LOQ	7.77	8.17	<LOQ	5.87	<LOQ	<LOQ
**Otu00006**	***Bacteroides dorei***	7.84	7.94	7.72	7.07	7.59	8.13	<LOQ	<LOQ	<LOQ	7.91	7.66	7.81	6.21	6.95	7.39	7.95	<LOQ	<LOQ	<LOQ	<LOQ
**Otu00025**	***Bacteroides finegoldii***	<LOQ	<LOQ	5.99	7.51	8.12	7.94	<LOQ	<LOQ	<LOQ	8.19	<LOQ	<LOQ	7.05	8.18	6.61	6.89	<LOQ	<LOQ	<LOQ	<LOQ
**Otu00020**	***Bacteroides salyersiae***	6.98	6.79	7.79	6.99	7.21	7.12	<LOQ	8.22	7.15	7.93	6.95	7.03	<LOQ	<LOQ	<LOQ	<LOQ	<LOQ	<LOQ	<LOQ	<LOQ
**Otu00012**	***Paraprevotella clara***	6.54	7.20	8.02	8.06	7.81	7.86	<LOQ	<LOQ	<LOQ	<LOQ	7.19	7.10	7.93	7.73	7.61	7.63	<LOQ	<LOQ	<LOQ	<LOQ
**Otu00017**	***Paraprevotella clara***	6.27	6.72	7.60	7.65	7.37	7.43	<LOQ	<LOQ	<LOQ	<LOQ	6.84	6.58	7.53	7.33	7.20	7.24	<LOQ	<LOQ	<LOQ	<LOQ
**Otu00030**	***Alistipes finegoldii/onder-donkii***	5.70	<LOQ	<LOQ	<LOQ	<LOQ	<LOQ	<LOQ	<LOQ	<LOQ	<LOQ	<LOQ	<LOQ	<LOQ	<LOQ	<LOQ	<LOQ	<LOQ	<LOQ	<LOQ	<LOQ
**Otu00015**	***Parabacteroides distasonis***	7.15	7.04	<LOQ	7.87	7.31	7.56	<LOQ	5.68	7.65	8.01	7.18	6.87	<LOQ	8.03	7.27	7.20	<LOQ	<LOQ	<LOQ	<LOQ
**Otu00016**	***Bilophila wadsworthia***	6.85	7.07	<LOQ	7.59	7.37	7.38	6.06	6.53	6.91	7.40	6.95	7.23	<LOQ	7.40	7.39	7.37	<LOQ	<LOQ	<LOQ	6.37
**Otu00001**	***Clostridium clos-tridioforme/bolteae***	8.37	8.32	<LOQ	<LOQ	8.28	8.36	<LOQ	<LOQ	8.96	8.79	8.53	8.54	<LOQ	8.60	8.80	8.65	<LOQ	5.99	8.30	7.83
**Otu00022**	***Clostridium aldenense***	5.70	<LOQ	<LOQ	8.57	6.62	5.72	<LOQ	<LOQ	<LOQ	<LOQ	5.90	5.70	<LOQ	5.89	6.78	6.62	<LOQ	<LOQ	<LOQ	<LOQ
**Otu00027**	***Hungatella hathewayi***	<LOQ	<LOQ	<LOQ	<LOQ	<LOQ	<LOQ	<LOQ	<LOQ	<LOQ	<LOQ	<LOQ	<LOQ	<LOQ	<LOQ	<LOQ	<LOQ	<LOQ	<LOQ	<LOQ	<LOQ
**Otu00031**	***Faecalibacterium prausnitzii***	<LOQ	<LOQ	<LOQ	<LOQ	<LOQ	<LOQ	<LOQ	<LOQ	<LOQ	<LOQ	<LOQ	<LOQ	<LOQ	<LOQ	<LOQ	<LOQ	<LOQ	<LOQ	<LOQ	<LOQ
**Otu00032**	***Selenomonas infelix***	<LOQ	<LOQ	<LOQ	<LOQ	<LOQ	<LOQ	<LOQ	<LOQ	<LOQ	<LOQ	<LOQ	<LOQ	<LOQ	<LOQ	<LOQ	<LOQ	<LOQ	<LOQ	<LOQ	<LOQ
**Otu00007**	***Veillonella dispar***	6.00	<LOQ	<LOQ	7.81	8.10	7.96	8.05	8.07	8.42	8.40	5.90	<LOQ	<LOQ	<LOQ	<LOQ	8.36	7.52	8.09	8.46	8.77
**Otu00028**	***Megasphaera sp.***	<LOQ	6.58	<LOQ	<LOQ	<LOQ	<LOQ	<LOQ	<LOQ	<LOQ	<LOQ	<LOQ	6.62	<LOQ	<LOQ	<LOQ	<LOQ	<LOQ	<LOQ	<LOQ	<LOQ
**Otu00002**	***Klebsiella pneumoniae***	8.27	7.49	8.18	7.90	7.67	7.48	8.84	8.68	6.88	6.71	6.87	6.77	7.74	7.57	7.37	7.54	8.63	9.07	6.31	6.99
**Otu00011**	***Klebsiella oxytoca***	7.58	7.03	7.95	7.10	6.79	6.99	8.33	7.90	7.04	8.00	7.74	7.55	8.04	7.32	7.48	8.27	8.40	8.10	6.83	6.92

**Otu00008**	***Citrobacter freundii***	<LOQ	<LOQ	<LOQ	<LOQ	<LOQ	<LOQ	5.79	5.86	<LOQ	<LOQ	<LOQ	<LOQ	<LOQ	<LOQ	<LOQ	<LOQ	5.75	6.24	<LOQ	<LOQ
**Otu00009**	***Escherichia coli/Shigella sonnei***	7.77	7.33	8.61	8.22	7.83	7.86	7.22	7.75	7.51	7.28	7.30	7.63	8.73	7.83	7.60	8.09	7.40	8.12	7.02	6.98
**Otu00010**	***Akkermansia muciniphila***	<LOQ	<LOQ	<LOQ	<LOQ	<LOQ	<LOQ	<LOQ	<LOQ	<LOQ	<LOQ	<LOQ	<LOQ	<LOQ	<LOQ	<LOQ	<LOQ	5.64	<LOQ	<LOQ	<LOQ

Table S11. Distal microbial community composition at OTU level for the LAC5V0, LAC10V0, LAC5V1 and LAC10V1 arms. Abundance of top 25 most abundant OTUs (log cells/mL) in arm 5 (LAC5V0), arm 6 (LAC10V0), arm 7 (LAC5V1) and arm 8 (LAC10V1) of the PathoGut™ SHIME experiment in the distal colon (DC) during the course of the control period (C_I-II), the clindamycin treatment period (CLI), the CDI stabilization period (CDI_I-III), the vancomycin treatment period (VNC) and the post-intervention period (PI_I-III) (n=1). The intensity of the shading indicates the absolute abundance, normalized for each of the different phyla (i.e., within each row).

OTU	Closely related species	DC																			
		LAC5V0										LAC10V0									
		C_I	C_II	CLI	CDI_I	CDI_II	CDI_III	VNC	PI_I	PI_II	PI_III	C_I	C_II	CLI	CDI_I	CDI_II	CDI_III	VNC	PI_I	PI_II	PI_III
**Otu00005**	***Bifidobacterium adolescentis***	8.26	7.96	<LOQ	<LOQ	<LOQ	<LOQ	<LOQ	<LOQ	<LOQ	<LOQ	8.36	8.14	<LOQ	<LOQ	<LOQ	<LOQ	<LOQ	<LOQ	6.28	<LOQ
**Otu00014**	***Bifidobacterium longum***	6.54	6.99	<LOQ	6.74	6.78	7.08	<LOQ	<LOQ	7.96	8.22	6.73	6.70	<LOQ	<LOQ	8.28	7.88	<LOQ	<LOQ	8.85	8.22
**Otu00013**	***Bacteroides fragilis***	6.88	7.59	<LOQ	<LOQ	<LOQ	6.77	<LOQ	<LOQ	5.60	<LOQ	7.40	8.68	6.06	6.32	8.01	8.12	<LOQ	<LOQ	7.94	7.19
**Otu00003**	***Bacteroides thetaiotaomicron***	8.31	8.35	8.69	8.36	7.55	7.40	<LOQ	7.37	7.75	7.89	8.07	7.96	8.49	8.28	8.14	7.91	<LOQ	8.25	8.91	7.70
**Otu00004**	***Bacteroides ovatus***	7.80	8.16	7.54	8.00	7.09	7.82	<LOQ	6.36	7.18	6.85	7.99	7.88	7.70	8.11	8.64	8.83	<LOQ	7.70	7.63	7.63
**Otu00006**	***Bacteroides dorei***	7.15	7.57	8.06	7.78	7.73	8.24	<LOQ	7.10	7.97	8.64	7.53	7.33	8.42	7.62	7.58	7.75	<LOQ	7.21	7.80	9.14
**Otu00025**	***Bacteroides finegoldii***	<LOQ	<LOQ	<LOQ	<LOQ	<LOQ	<LOQ	<LOQ	<LOQ	<LOQ	<LOQ	<LOQ	<LOQ	<LOQ	<LOQ	<LOQ	<LOQ	<LOQ	<LOQ	<LOQ	<LOQ
**Otu00020**	***Bacteroides salyersiae***	6.37	6.85	7.36	6.16	6.34	6.60	<LOQ	6.32	8.09	7.04	6.48	6.55	6.88	6.74	6.91	6.94	<LOQ	<LOQ	<LOQ	<LOQ
**Otu00012**	***Paraprevotella clara***	<LOQ	5.70	6.83	6.48	5.91	6.38	<LOQ	<LOQ	<LOQ	<LOQ	6.20	<LOQ	6.30	6.29	6.75	6.93	<LOQ	<LOQ	<LOQ	<LOQ
**Otu00017**	***Paraprevotella clara***	<LOQ	<LOQ	6.45	5.93	<LOQ	6.17	<LOQ	<LOQ	<LOQ	<LOQ	5.86	<LOQ	5.89	5.69	6.39	6.62	<LOQ	<LOQ	<LOQ	<LOQ
**Otu00030**	***Alistipes finegoldii/onderdonkii***	5.77	6.47	<LOQ	<LOQ	<LOQ	<LOQ	<LOQ	<LOQ	<LOQ	5.72	<LOQ	5.87	<LOQ	<LOQ	<LOQ	<LOQ	<LOQ	<LOQ	<LOQ	<LOQ
**Otu00015**	***Parabacteroides distasonis***	6.83	7.26	7.55	7.36	6.71	7.39	<LOQ	7.47	6.51	6.94	7.17	7.19	<LOQ	7.30	7.60	7.56	<LOQ	7.24	7.14	7.57
**Otu00016**	***Bilophila wadsworthia***	6.65	6.99	<LOQ	7.78	6.95	7.02	7.45	6.39	5.90	6.38	7.00	6.98	<LOQ	8.04	7.65	7.73	7.73	6.77	6.68	7.55
**Otu00001**	***Clostridium clostridioforme/bolteae***	8.40	8.31	<LOQ	8.93	8.07	8.57	5.63	<LOQ	7.67	7.86	8.68	8.20	<LOQ	8.33	8.55	8.49	<LOQ	<LOQ	7.93	8.31
**Otu00022**	***Clostridium aldenense***	6.53	6.66	<LOQ	7.51	6.96	6.61	<LOQ	<LOQ	<LOQ	6.42	6.65	6.36	<LOQ	7.54	7.66	7.51	<LOQ	<LOQ	8.63	6.84
**Otu00027**	***Hungatella hathewayi***	<LOQ	6.06	<LOQ	7.83	5.86	6.13	<LOQ	<LOQ	6.08	6.36	6.08	<LOQ	<LOQ	6.47	6.59	6.28	<LOQ	<LOQ	<LOQ	<LOQ
**Otu00031**	***Faecalibacterium prausnitzii***	<LOQ	<LOQ	<LOQ	6.66	<LOQ	<LOQ	<LOQ	<LOQ	<LOQ	<LOQ	<LOQ	<LOQ	<LOQ	7.16	6.29	<LOQ	<LOQ	<LOQ	<LOQ	<LOQ
**Otu00032**	***Selenomonas infelix***	<LOQ	<LOQ	<LOQ	<LOQ	<LOQ	<LOQ	<LOQ	<LOQ	<LOQ	<LOQ	<LOQ	<LOQ	<LOQ	<LOQ	<LOQ	<LOQ	<LOQ	<LOQ	<LOQ	<LOQ
**Otu00007**	***Veillonella dispar***	<LOQ	<LOQ	<LOQ	7.51	6.66	6.83	8.71	7.33	7.56	7.51	<LOQ	<LOQ	<LOQ	<LOQ	<LOQ	<LOQ	<LOQ	8.09	8.64	8.56
**Otu00028**	***Megasphaera sp.***	6.82	7.16	<LOQ	6.96	6.74	6.80	<LOQ	<LOQ	6.83	6.95	6.55	6.89	<LOQ	<LOQ	<LOQ	<LOQ	<LOQ	<LOQ	<LOQ	<LOQ
**Otu00002**	***Klebsiella pneumoniae***	6.41	<LOQ	7.93	5.68	6.15	5.66	7.65	8.67	5.60	5.88	6.86	<LOQ	8.21	7.56	7.27	6.79	9.01	9.28	5.81	5.93

**Otu00011**	***Klebsiella oxytoca***	6.41	6.17	7.93	6.72	6.03	6.53	8.98	8.52	6.88	6.61	6.34	<LOQ	7.52	7.16	7.76	7.72	9.09	8.69	6.46	6.36
**Otu00008**	***Citrobacter freundii***	<LOQ	<LOQ	7.33	7.02	5.67	<LOQ	8.91	9.24	6.75	<LOQ	<LOQ	<LOQ	6.46	6.06	6.46	6.28	7.26	8.02	5.98	5.93
**Otu00009**	***Escherichia coli/Shigella sonnei***	5.89	5.62	8.65	7.28	7.28	7.69	6.72	7.67	7.33	7.40	6.13	5.77	8.59	8.25	7.79	7.51	6.78	7.84	7.08	7.18
**Otu00010**	***Akkermansia muciniphila***	7.26	8.61	<LOQ	<LOQ	9.14	9.18	<LOQ	<LOQ	9.27	9.13	8.31	8.27	<LOQ	<LOQ	<LOQ	<LOQ	<LOQ	<LOQ	<LOQ	<LOQ

**OTU**	**Closely related species**	**DC**																			
		**LAC5V1**										**LAC10V1**									
		**C_I**	**C_II**	**CLI**	**CDI_I**	**CDI_II**	**CDI_III**	**VNC**	**PI_I**	**PI_II**	**PI_III**	**C_I**	**C_II**	**CLI**	**CDI_I**	**CDI_II**	**CDI_III**	**VNC**	**PI_I**	**PI_II**	**PI_III**
**Otu00005**	***Bifidobacterium adolescentis***	8.44	8.31	<LOQ	<LOQ	<LOQ	<LOQ	<LOQ	<LOQ	<LOQ	<LOQ	8.51	8.32	<LOQ	<LOQ	7.54	8.58	<LOQ	<LOQ	8.86	8.88
**Otu00014**	***Bifidobacterium longum***	6.12	6.69	<LOQ	<LOQ	8.00	7.86	<LOQ	<LOQ	7.95	8.07	6.26	6.65	<LOQ	<LOQ	<LOQ	<LOQ	<LOQ	<LOQ	<LOQ	<LOQ
**Otu00013**	***Bacteroides fragilis***	7.22	7.77	<LOQ	<LOQ	<LOQ	8.11	<LOQ	<LOQ	<LOQ	<LOQ	7.06	7.06	7.39	5.93	6.92	7.77	<LOQ	<LOQ	<LOQ	<LOQ
**Otu00003**	***Bacteroides thetaiotaomicron***	8.55	8.10	8.37	8.68	8.09	7.58	<LOQ	8.35	8.87	8.17	8.04	8.31	8.39	7.31	8.04	7.76	<LOQ	7.66	8.43	7.66
**Otu00004**	***Bacteroides ovatus***	8.44	8.41	7.52	6.91	7.22	7.42	<LOQ	7.53	7.28	7.36	8.41	8.21	7.45	6.68	7.47	7.65	<LOQ	6.79	8.02	7.40
**Otu00006**	***Bacteroides dorei***	7.75	7.93	8.21	8.19	7.91	7.94	<LOQ	6.36	7.82	8.83	7.98	7.86	8.03	7.55	8.07	8.00	<LOQ	6.74	8.02	8.70
**Otu00025**	***Bacteroides finegoldii***	<LOQ	<LOQ	<LOQ	7.35	7.66	7.12	<LOQ	<LOQ	<LOQ	7.46	<LOQ	<LOQ	7.31	7.86	6.75	6.48	<LOQ	<LOQ	<LOQ	<LOQ
**Otu00020**	***Bacteroides salyersiae***	6.77	6.71	6.72	7.00	6.66	6.87	<LOQ	7.56	8.57	7.01	6.63	6.74	6.55	6.34	7.55	6.91	<LOQ	<LOQ	<LOQ	<LOQ
**Otu00012**	***Paraprevotella clara***	<LOQ	<LOQ	6.72	6.93	6.83	6.14	<LOQ	<LOQ	<LOQ	<LOQ	<LOQ	<LOQ	6.53	6.48	6.99	6.65	<LOQ	<LOQ	<LOQ	<LOQ
**Otu00017**	***Paraprevotella clara***	<LOQ	<LOQ	6.19	6.50	6.51	5.72	<LOQ	<LOQ	<LOQ	<LOQ	<LOQ	<LOQ	6.07	6.08	6.62	6.36	<LOQ	<LOQ	<LOQ	<LOQ
**Otu00030**	***Alistipes finegoldii/onderdonkii***	5.99	5.95	<LOQ	<LOQ	<LOQ	<LOQ	<LOQ	<LOQ	<LOQ	<LOQ	5.69	5.94	<LOQ	<LOQ	<LOQ	<LOQ	<LOQ	<LOQ	<LOQ	<LOQ
**Otu00015**	***Parabacteroides distasonis***	7.25	7.59	<LOQ	7.55	7.56	7.63	<LOQ	6.94	7.43	7.22	7.23	7.41	6.65	6.86	7.62	7.39	<LOQ	7.89	7.98	8.18
**Otu00016**	***Bilophila wadsworthia***	6.84	7.45	<LOQ	8.00	7.53	7.61	7.03	6.58	7.05	7.46	7.11	7.38	<LOQ	7.67	7.47	7.30	6.45	6.37	7.16	7.27
**Otu00001**	***Clostridium clostridioforme/bolteae***	8.26	8.40	<LOQ	8.10	8.67	8.58	<LOQ	<LOQ	8.84	8.64	8.42	8.60	<LOQ	8.40	8.85	8.81	<LOQ	<LOQ	8.34	7.99
**Otu00022**	***Clostridium aldenense***	6.55	6.52	<LOQ	8.52	7.53	7.23	<LOQ	<LOQ	6.98	6.89	6.41	6.56	<LOQ	7.17	6.98	7.08	<LOQ	<LOQ	6.96	6.75
**Otu00027**	***Hungatella hathewayi***	<LOQ	5.95	<LOQ	7.83	7.94	7.20	<LOQ	<LOQ	<LOQ	<LOQ	5.93	6.06	<LOQ	7.05	6.49	6.31	<LOQ	<LOQ	<LOQ	<LOQ
**Otu00031**	***Faecalibacterium prausnitzii***	<LOQ	<LOQ	<LOQ	7.43	<LOQ	5.64	<LOQ	<LOQ	<LOQ	<LOQ	<LOQ	<LOQ	<LOQ	6.83	<LOQ	5.61	<LOQ	<LOQ	<LOQ	<LOQ
**Otu00032**	***Selenomonas infelix***	<LOQ	<LOQ	<LOQ	<LOQ	<LOQ	<LOQ	<LOQ	<LOQ	<LOQ	<LOQ	<LOQ	<LOQ	<LOQ	<LOQ	<LOQ	<LOQ	<LOQ	<LOQ	<LOQ	<LOQ
**Otu00007**	***Veillonella dispar***	<LOQ	<LOQ	<LOQ	8.12	8.24	7.80	5.67	7.81	8.27	7.83	<LOQ	<LOQ	<LOQ	<LOQ	<LOQ	7.70	<LOQ	7.28	8.69	8.77
**Otu00028**	***Megasphaera sp.***	6.47	6.89	<LOQ	<LOQ	<LOQ	<LOQ	<LOQ	<LOQ	<LOQ	<LOQ	6.24	7.06	<LOQ	<LOQ	<LOQ	<LOQ	<LOQ	<LOQ	<LOQ	<LOQ
**Otu00002**	***Klebsiella pneumoniae***	6.79	6.87	8.09	7.35	7.19	6.95	9.04	9.36	6.44	<LOQ	5.69	6.01	7.64	7.12	7.00	7.27	9.23	9.45	5.91	6.21
**Otu00011**	***Klebsiella oxytoca***	<LOQ	6.25	7.63	6.98	6.45	6.64	8.09	8.43	6.98	6.58	6.50	6.89	7.99	7.15	7.35	7.81	8.14	8.10	6.92	6.83

**Otu00008**	***Citrobacter freundii***	<LOQ	<LOQ	<LOQ	<LOQ	<LOQ	<LOQ	8.09	8.15	6.51	<LOQ	<LOQ	<LOQ	<LOQ	<LOQ	<LOQ	<LOQ	7.59	7.59	6.69	<LOQ
**Otu00009**	***Escherichia coli/Shigella sonnei***	<LOQ	<LOQ	8.72	7.65	7.21	6.85	5.67	6.97	6.39	5.81	<LOQ	6.01	8.79	7.53	7.25	7.30	5.83	7.53	6.95	6.85
**Otu00010**	***Akkermansia muciniphila***	7.35	8.17	<LOQ	<LOQ	<LOQ	<LOQ	<LOQ	<LOQ	<LOQ	<LOQ	6.97	7.90	<LOQ	<LOQ	<LOQ	<LOQ	<LOQ	<LOQ	<LOQ	<LOQ

Figure S1. Total viable cell counts during the experiment. Total viable counts quantified by flow cytometry during the course of the PathoGut™ SHIME experiment in the proximal (PC; A and C) and distal colon (DC; B and D) reactors for each of the eight experimental arms (arm 1: CTRL; arm 2: AB; arm 3: LAC5; arm 4: LAC10; arm 5: LAC5V0; arm 6: LAC10V0; arm 7: LAC5V1; arm 8: LAC10V1). Panels A and B show the results obtained for arms 1-4, including samples collected during the control period (n=2), the clindamycin treatment period (n=1) and the CDI stabilization period (n=3) in the PC and DC, respectively. Panels C and D show the results obtained for arms 1-2 and 5-8, including samples collected during the control period (n=2), the clindamycin treatment period (n=1), the CDI stabilization period (n=3), the vancomycin treatment period (n=1) and the post-intervention period (n=3) in the PC and DC, respectively. Dots represent single analysis of the collected samples obtained at the different time points.

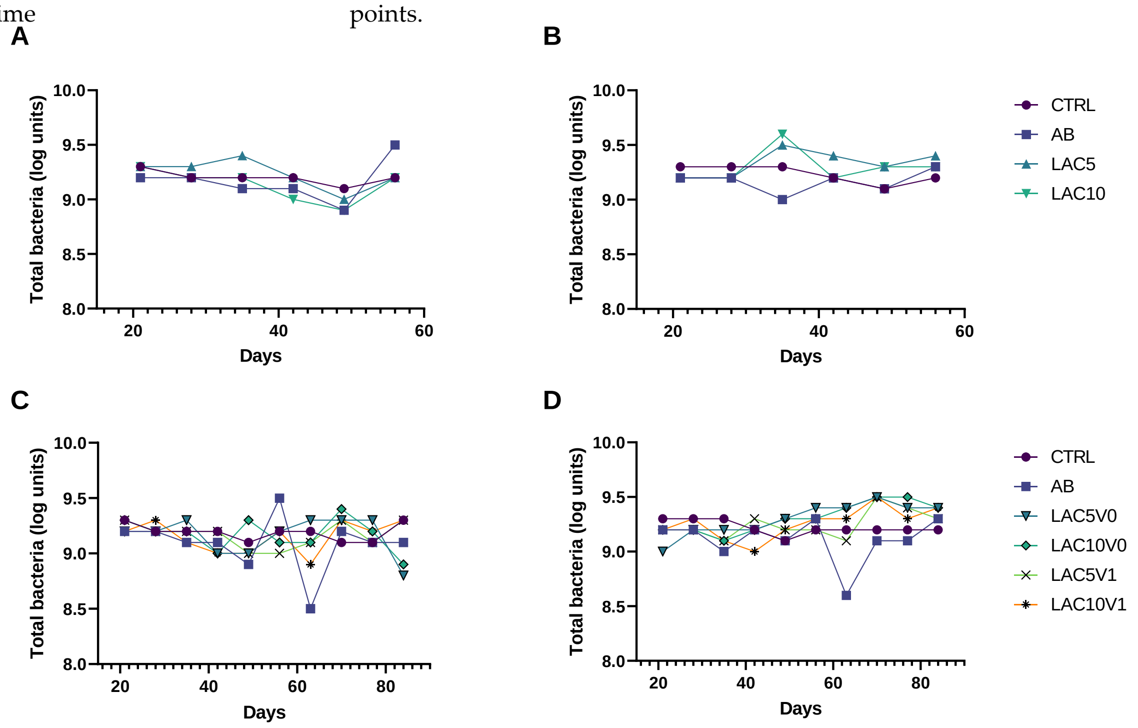

Figure S2. Microbial metabolic activity for the control arms. Bacterial metabolites (acetate, propionate, butyrate, BCFA and total SCFA) levels (mM) during the course of the PathoGut™ SHIME experiment in the proximal (PC; A, C and E) and distal colon (DC; B, D and F) reactors for each of the experimental periods, i.e., control period (C), clindamycin treatment period (CLI), *C. difficile* infection stabilization period (CDI), vancomycin treatment period (VNC) and post-intervention period (PI). Panels A and B represent the levels obtained in blank control arm (arm 1; CTRL) of the PathoGut™ SHIME experiment, while panels C and D represent the levels obtained in the antibiotic-treated control arm (arm 2; AB) of the PathoGut™ SHIME experiment. Dots represent mean ± SEM for each of the experimental periods (n=3 per week). Panels E and F represent the levels obtained in the different arms of the PathoGut™ SHIME experiment treated with lactulose (arms 3-8). Bars represent the mean ± SEM (n=6 for C; n=6 for CLI; and n=4 for VNC). Statistically significant differences between the blank control arm (arm 1; CTRL) and the arms supplemented with lactulose or between the antibiotic-treated control arm (arm 2; AB) and the arms supplemented with lactulose are marked with "*" (with * p<0.05, ** p<0.01, *** p<0.001; **** p<0.0001) or "#" (with # p<0.05, ## p<0.01, ### p<0.001; #### p<0.0001), respectively.

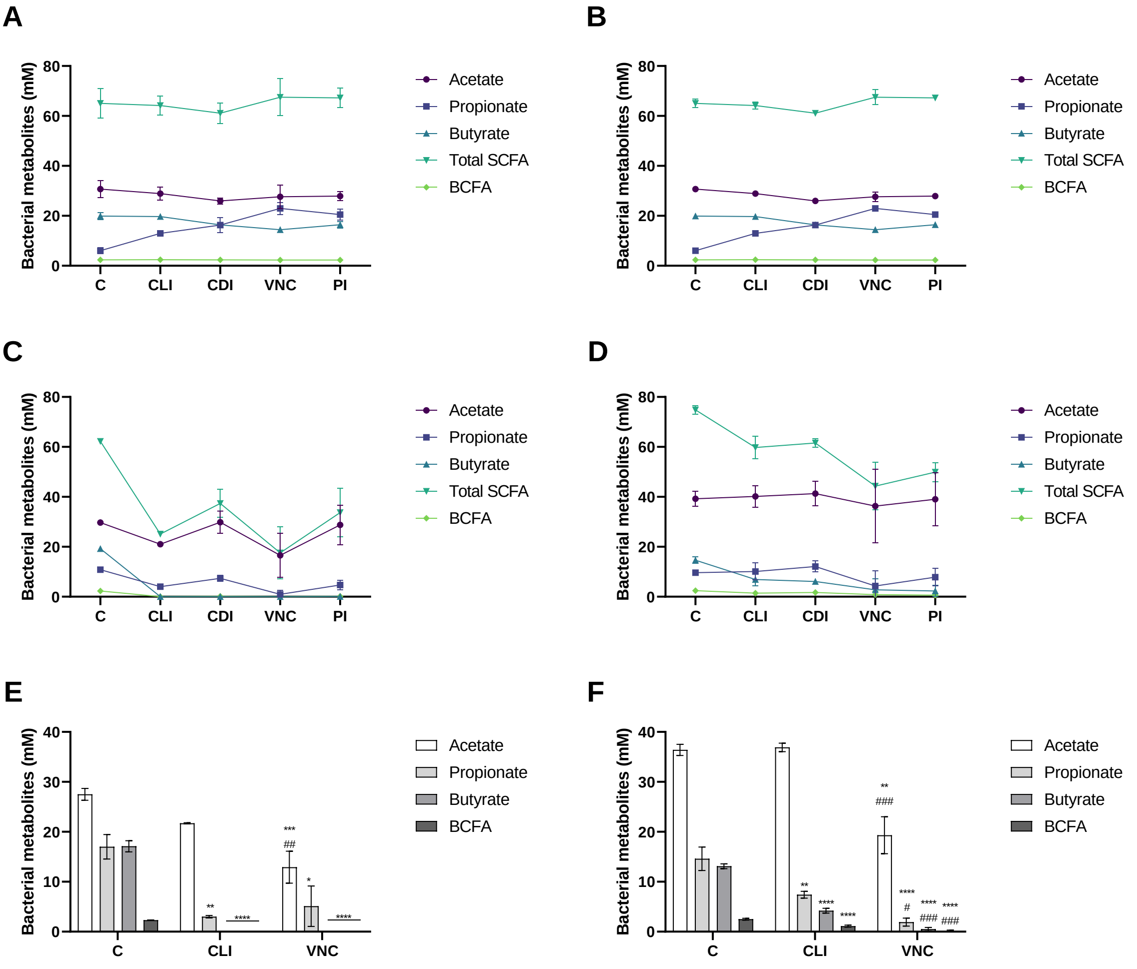

Figure S3. *C. difficile* quantification during the clindamycin treatment period. Quantification of *C. difficile* in the PathoGut™ SHIME during the clindamycin treatment period (CLI). Bars represent mean ± SEM [n=8 biological replicas (reactors), n=3 technical replicas] of vegetative cell (VC) and spore germination (SG) counts of *C. difficile* in the proximal colon compartment after administration of the first (light grey bar) and second (dark grey bars) dose of *C. difficile* spores during the clindamycin treatment period (CLI).

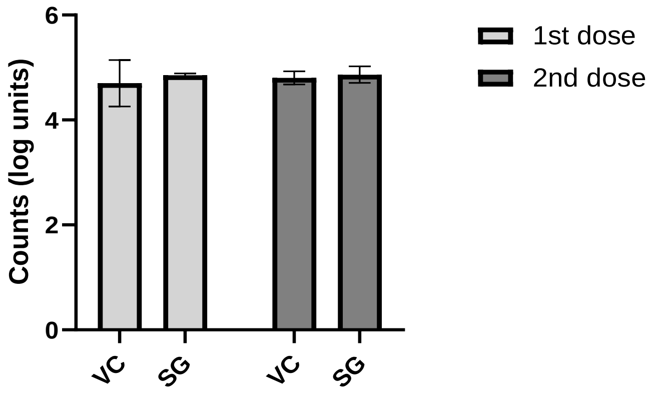

Figure S4. Base, lactate and ammonium levels in the CTRL, AB, LAC5 and LAC10 arms. Base (NaOH) consumption (mL/day), lactate levels (mM) and ammonium (NH4+) levels (mg/L) in the proximal (A) and distal colon (B) reactors during the course of the control period (C_I-II), the clindamycin treatment period (CLI) and the CDI stabilization period (CDI_I-III) of the PathoGut™ SHIME experiment for four of the experimental arms, including arm 1 (CTRL), arm 2 (AB), arm 3 (LAC5) and arm 4 (LAC10). Dots represent mean ± SEM (n = 3).

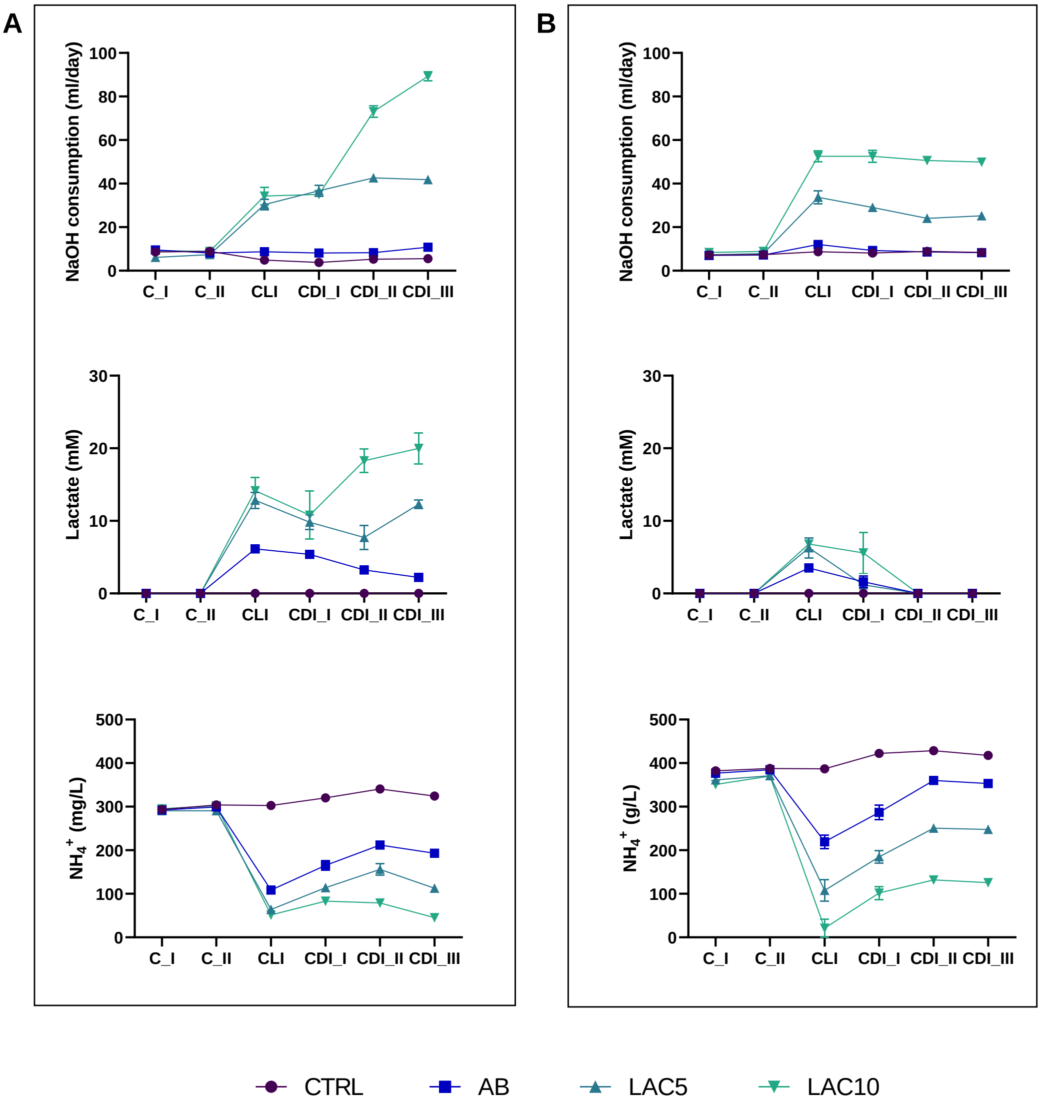

Figure S5. Effect of lactulose and clindamycin on microbial metabolic activity. Effect of clindamycin and lactulose on (A) short chain fatty acids levels (mM) in the proximal colon, including acetate, propionate, butyrate and branched chain fatty acids (BCFA), and (B) bile acids profile in the distal colon, including taurocholic acid (TCA), taurochenodeoxycholic acid (TCDCA), glycocholic acid (GCA), deoxycholic acid (DCA) and cholic acid (CA) levels (mM), of the PathoGut™ SHIME experiment for four of the experimental arms, including arm 1 (CTRL), arm 2 (AB), arm 3 (LAC5) and arm 4 (LAC10). Bars represent mean ± SEM of different time points during the clindamcyin treatment (CLI) and CDI stabilization periods (n = 12; d29, d31, d33, d36, d38, d40, d43, d45, d47, d50, d52, d54). Statistically significant differences between lactulose treatments (LAC5 and LAC10) and CTRL or AB arms are marked with "*" (* $p < 0.05$, ** $p < 0.01$, *** $p < 0.001$; **** $p < 0.0001$) or "#" (# $p < 0.05$, ## $p < 0.01$, ### $p < 0.001$; #### $p < 0.0001$), respectively.

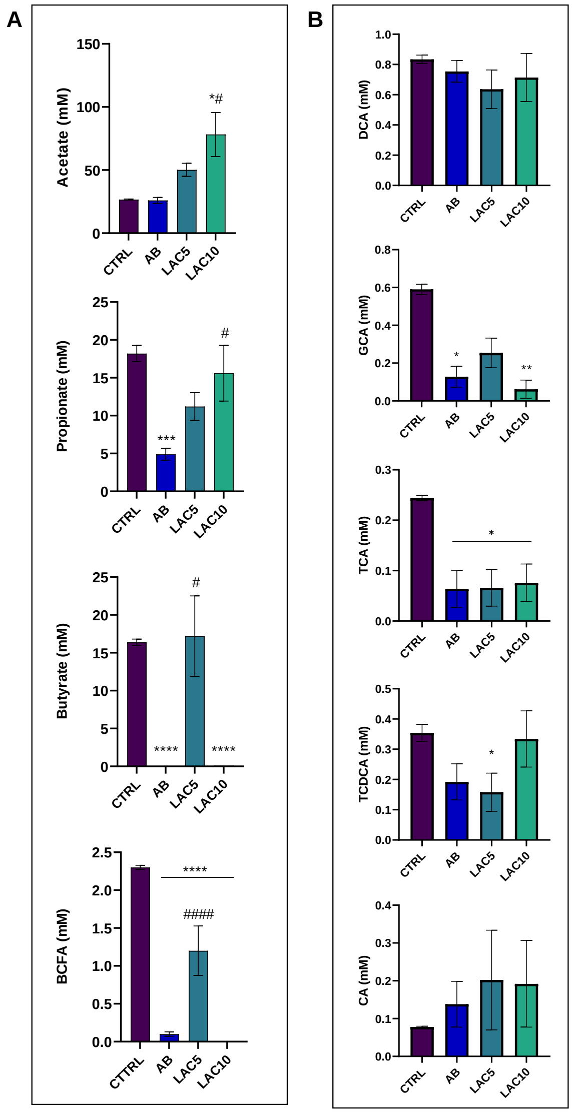

Figure S6. Distal SCFA levels for the CTRL, AB, LAC5 and LAC10 arms. Effect of clindamycin and lactulose on short chain fatty acids profile in the distal colon of the PathoGut™ SHIME experiment for four of the experimental arms, including arm 1 (CTRL), arm 2 (AB), arm 3 (LAC5) and arm 4 (LAC10). (A) Acetate, propionate, butyrate and branched-chain fatty acid (BCFA) levels (mM) during the course of the control period (C_I-II), the clindamycin treatment period (CLI) and the CDI stabilization period (CDI_I-III). Dots represent mean ± SEM (n=3). (B) Bar plots of acetate, propionate, butyrate and BCFA, representing mean ± SEM of different time points during the CLI and CDI stabilization periods (n = 12; d29, d31, d33, d36, d38, d40, d43, d45, d47, d50, d52, d54). Statistically significant differences between lactulose treatments (LAC5 and LAC10) and CTRL or AB arms are marked with "*" (* $p < 0.05$, ** $p < 0.01$, *** $p < 0.001$; **** $p < 0.0001$) or "#" (# $p < 0.05$, ## $p < 0.01$, ### $p < 0.001$; #### $p < 0.0001$), respectively.

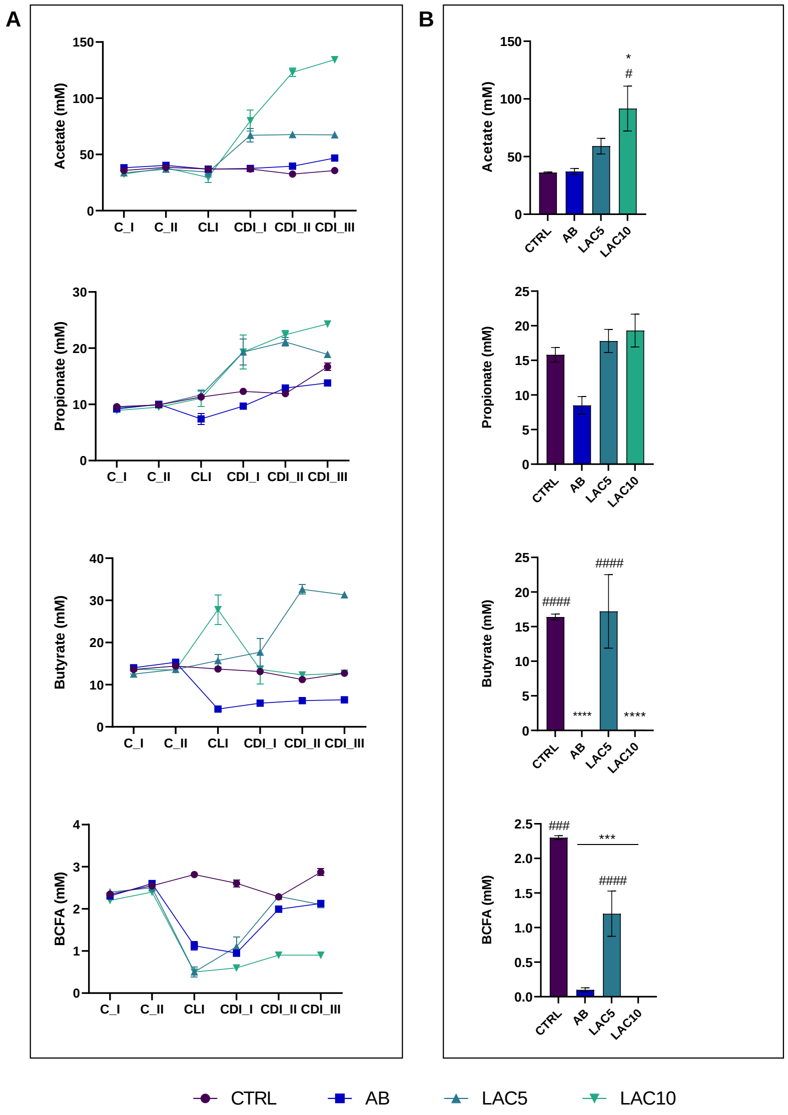

Figure S7. Effect of lactulose and vancomycin on microbial metabolic activity. Effect of lactulose on vancomycin-disrupted microbial metabolic activity for (A) short chain fatty acids levels (mM) in the proximal colon, including acetate, propionate, butyrate and branched chain fatty acids (BCFA), and (B) bile acids profile in the distal colon, including taurocholic acid (TCA), taurochenodeoxycholic acid (TCDCA), glycocholic acid (GCA), deoxycholic acid (DCA) and cholic acid (CA) levels (mM), of the PathoGut™ SHIME experiment for six of the experimental arms, including arm 1 (CTRL), arm 2 (AB), arm 5 (LAC5V0), arm 6 (LAC10V0), arm 7 (LAC5V1) and arm 8 (LAC10V1). Bars represent mean ± SEM of different time points during the vancomycin treatment (VNC) and post-intervention (PI) periods (n = 12; d57, d59, d61, d64, d66, d68, d71, d73, d75, d78, d80, d82). Statistically significant differences between lactulose treatments (LAC5V0, LAC10V0, LAC5V1 and LAC10V1) and CTRL or AB arms are marked with "*" (* $p < 0.05$, ** $p < 0.01$, *** $p < 0.001$; **** $p < 0.0001$) or "#" (# $p < 0.05$, ## $p < 0.01$, ### $p < 0.001$; #### $p < 0.0001$), respectively.

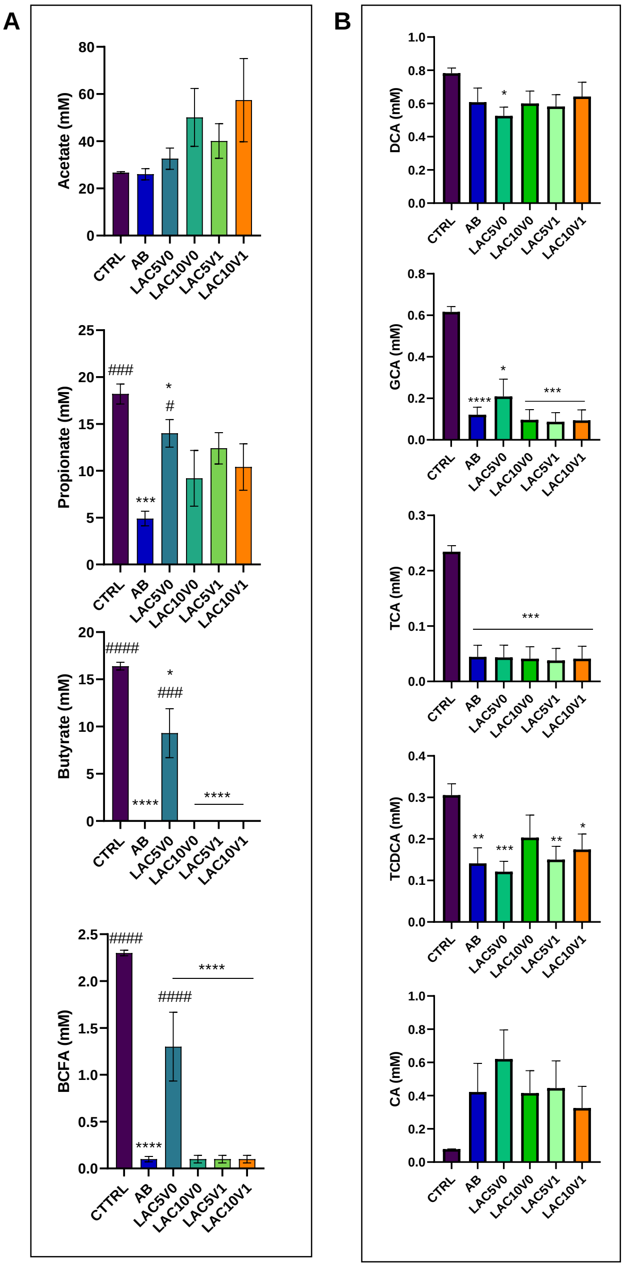

Figure S8. Base, lactate and ammonium levels in the CTRL, AB, LAC5V0, LAC10V0, LAC5V1 and LAC10V1 arms. Base (NaOH) consumption (mL/day), lactate levels (mM) and ammonium (NH4+) levels (mg/L) in the proximal (A) and distal colon (B) reactors during the course of the control period (C_I-II), the clindamycin treatment period (CLI), the CDI stabilization period (CDI_I-III), the vancomycin treatment period (VNC) and the post-intervention period (PI_I-III) of the PathoGut™ SHIME experiment for six of the experimental arms, including arm 1 (CTRL), arm 2 (AB), arm 5 (LAC5V0), arm 6 (LAC10V0), arm 7 (LAC5V1) and arm 8 (LAC10V1). Dots represent mean ± SEM (n = 3).

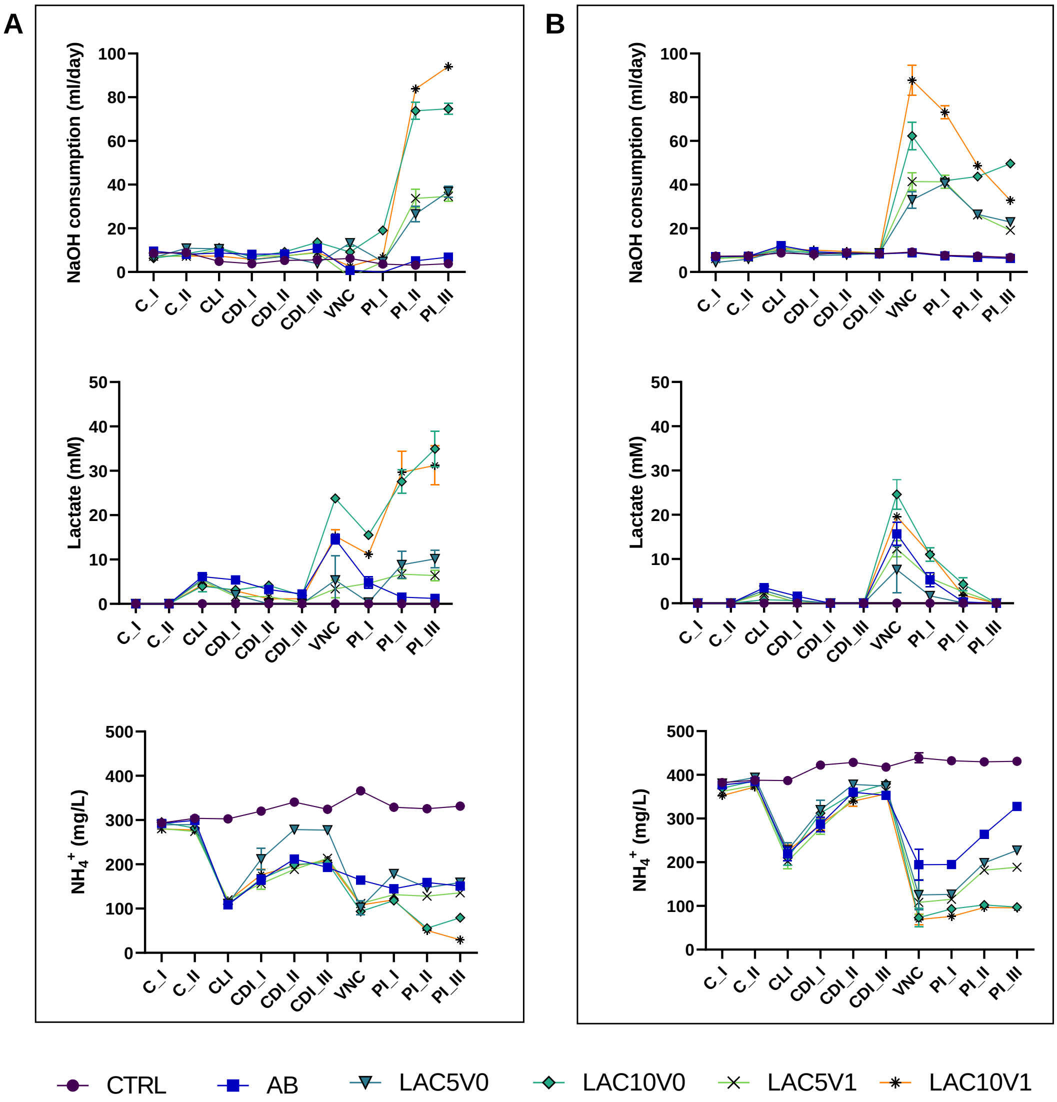

Figure S9. Antibiotic effect as indicated in DAPC plot. Discriminant analysis of principal components (DAPC) plot at genus level of control (CTRL), clindamycin (CLI) and vancomycin (VNC) exposed reactors during the PathoGut™ SHIME experiment. Adonis test based on Bray Curtis Distance indicates a statistically significant differences ($p < 0.001$) between groups.

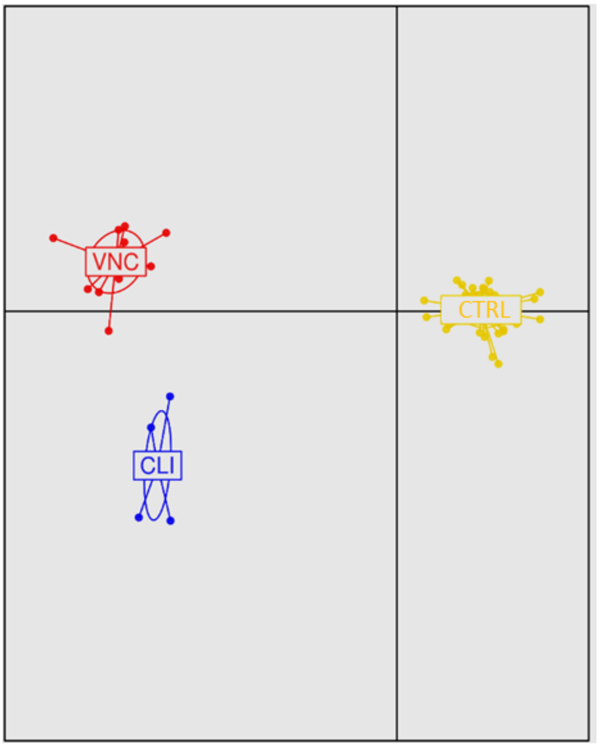

Figure S10. Effect of antibiotics on microbial community composition. (A) Evolution of microbial community at phylum level in the colonic reactors (PC and DC) during the course of the control period (C), the clindamycin treatment period (CLI), the CDI stabilization period (CDI), the vancomycin treatment period (VNC) and the post-intervention period (PI) of the PathoGut™ SHIME experiment for (A) arm 1 (CTRL) and (B) arm 2 (AB). (C) LEfSe analysis at phylum and family level comparing different sampling time points during the course of the control period (C), the clindamycin treatment period (CLI), the CDI stabilization period (CDI), the vancomycin treatment period (VNC) and the post-intervention period (PI) of the PathoGut™ SHIME experiment for arm 2 (AB), including LDA scores (log 10 units) ≥ 4.

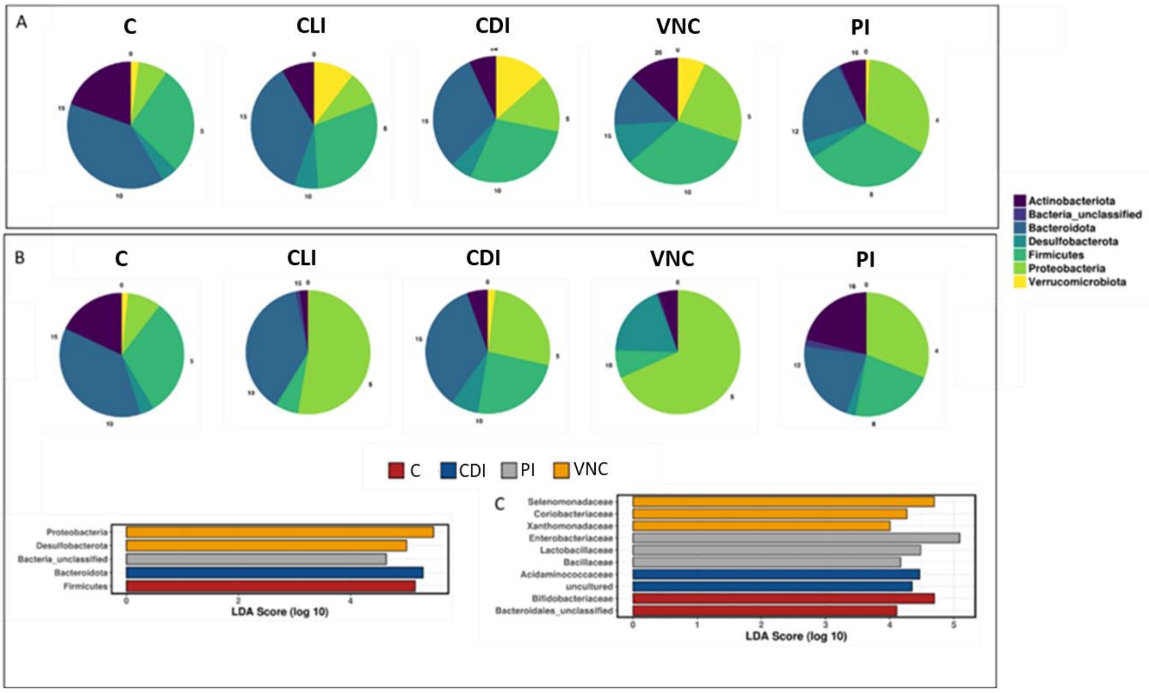

Figure S11. Effect of antibiotic treatment on microbial diversity. Diversity indices Shannon, Chao1, Richness and Simpson's index at OTU level for control (CTRL), clindamycin (CLI) and vancomycin (VNC) exposed reactors during the PathoGut™ SHIME experiment. Statistically significant differences between antibiotic treatment (CLI and VNC)) and CTRL are marked with "*" (* $p < 0.05$, ** $p < 0.01$, *** $p < 0.001$; **** $p < 0.0001$).

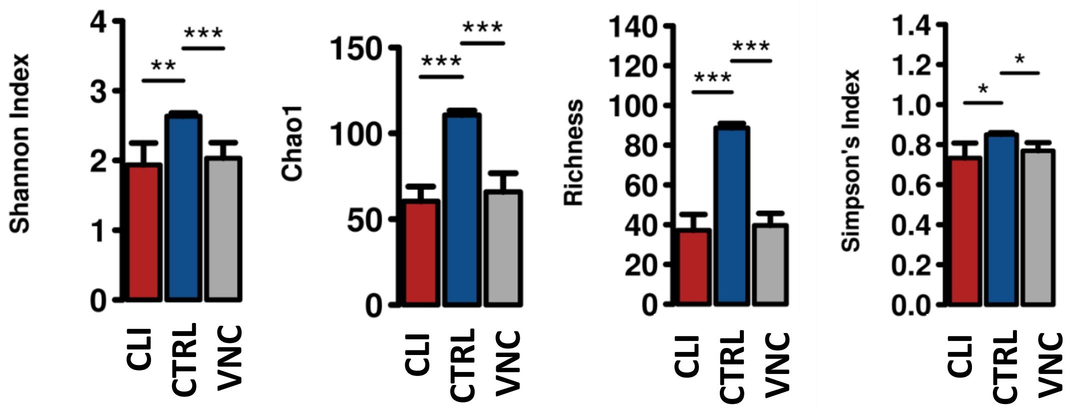

Figure S12. Abundance of OTU99 in the distal colon. Differences in abundance of OTU99 in the distal colon (DC) of the PathoGut™ SHIME experiment for the eight experimental arms, including arm 1 (CTRL), arm 2 (AB), arm 3 (LAC5), arm 4 (LAC10), arm 5 (LAC5V0), arm 6 (LAC10V0), arm 7 (LAC5V1) and arm 8 (LAC10V1) following clindamycin (CLI) and/or vancomycin (VNC) treatment. Data represent all the time points obtained for each of the test conditions.

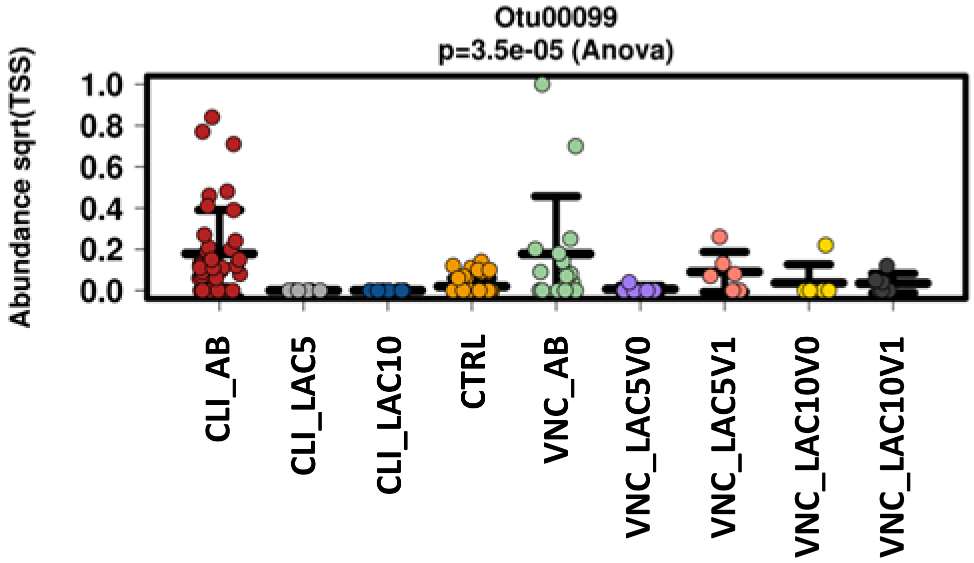

Figure S13. qPCR results in the distal colon for the CTRL, AB, LAC5 and LAC10 arms. qPCR counts (copies/mL log units) of Firmicutes, Bacteroidetes, *Enterobacteriaceae*, lactobacilli, bifidobacteria and *Akkermansia muciniphila* in the distal colon during the course of the control period (C_I-II), the clindamycin treatment period (CLI) and the CDI stabilization period (CDI_I-III) of the PathoGut™ SHIME experiment for four of the experimental arms, including arm 1 (CTRL), arm 2 (AB), arm 3 (LAC5) and arm 4 (LAC10). Dots represent mean ± SEM of technical replicas of qPCR of one time point per period.

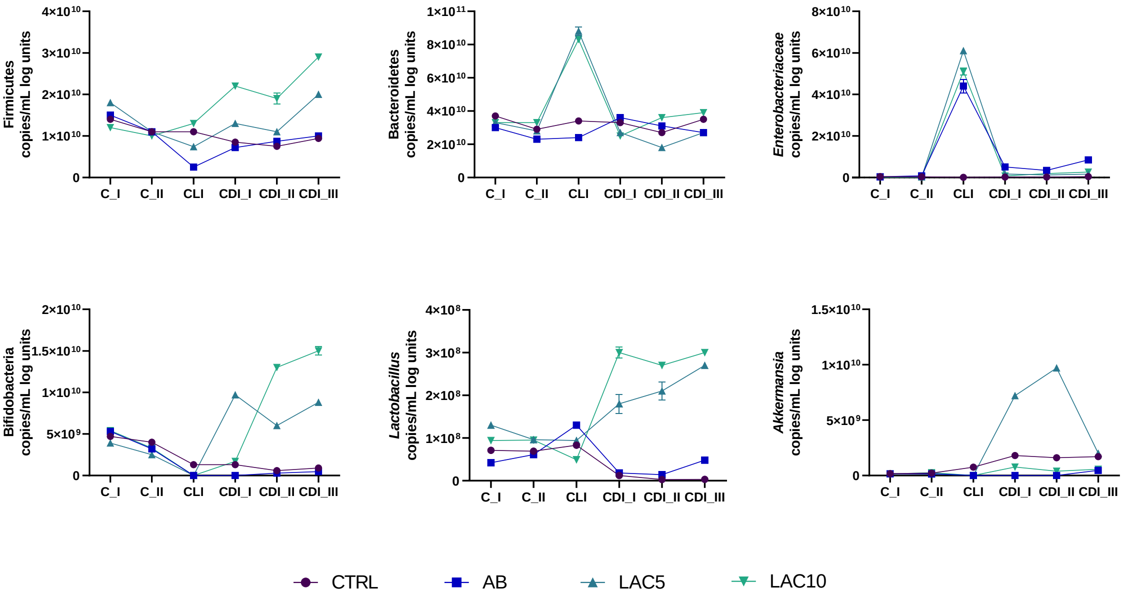

Figure S14. Effect of lactulose treatment on *Bifidobacteriaceae* levels during the post-intervention period. Mixed effect analysis on *Bifidobacteriaceae* levels during the post-intervention period (PI I-III) of the PathoGut™ SHIME experiment for five experimental arms, including arm 2 (AB), arm 5 (LAC5V0), arm 6 (LAC10V0), arm 7 (LAC5V1) and arm 8 (LAC10V1) following vancomycin (VNC) treatment.

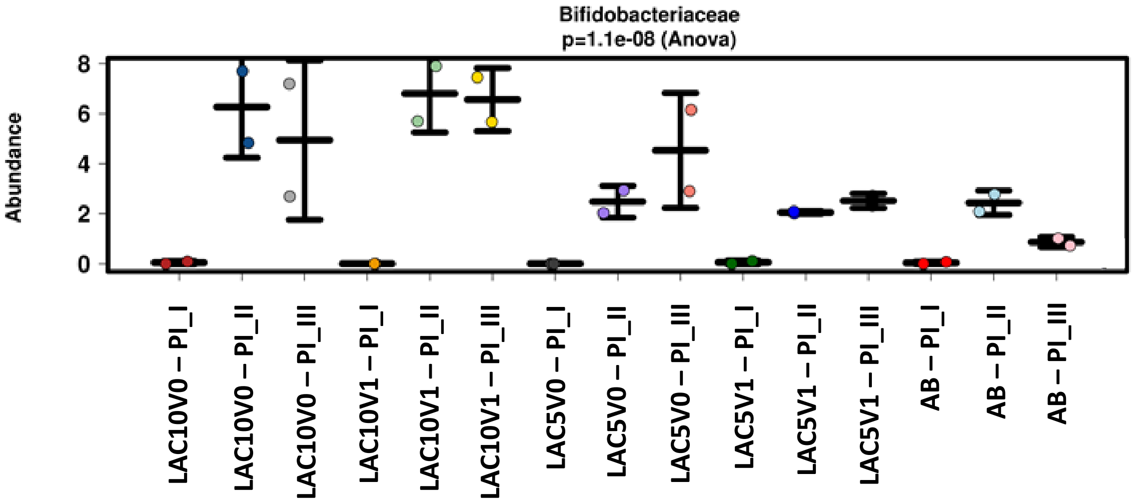

Figure S15. Effect on microbial community composition following vancomycin treatment. Linear discriminant analysis effect size (LEfSe) analysis at OTU level during the PathoGut™ SHIME experiment for six experimental arms, including arm 1 (CTRL), arm 2 (AB), arm 5 (LAC5V0), arm 6 (LAC10V0), arm 7 (LAC5V1) and arm 8 (LAC10V1) following the vancomycin treatment period (VNC).

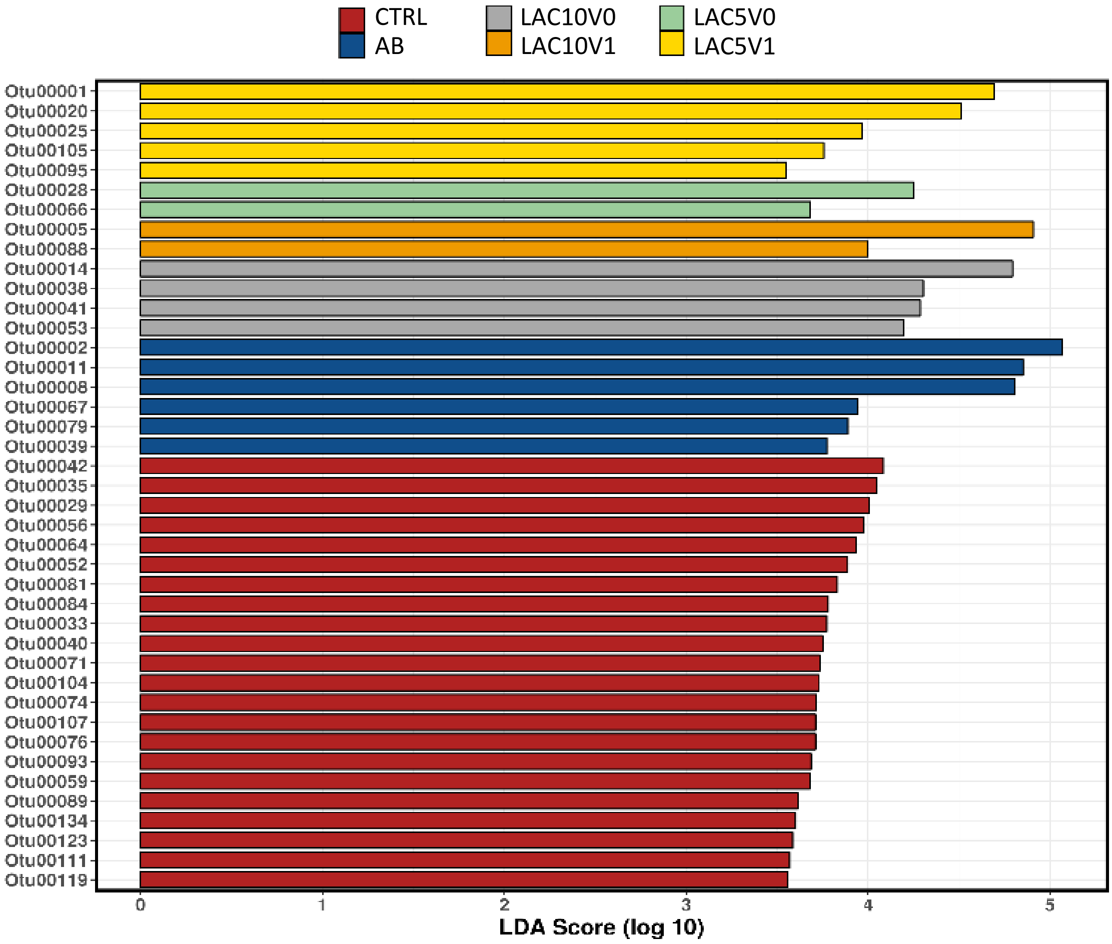

Figure S16. Effect of lactulose on microbial diversity following antibiotic treatment. Diversity indices Shannon, Chao1, Simpson's and Richness index at OTU level during the PathoGut™ SHIME experiment for control (CTRL), clindamycin (CLI) and vancomycin (VNC) exposed reactors with or without lactulose (LAC) supplementation.

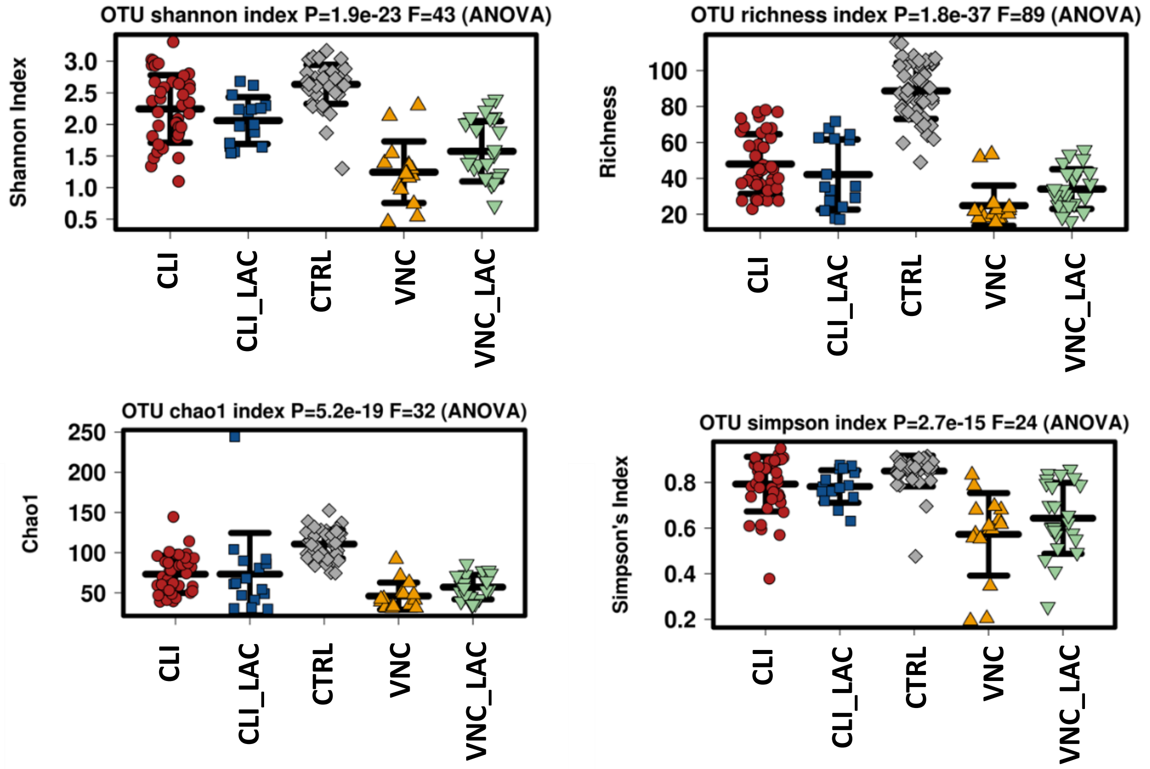

Figure S17. qPCR results in the distal colon for the CTRL, AB, LAC5V0, LAC10V0, LAC5V1 and LAC10V1 arms. qPCR counts (copies/mL log units) of Firmicutes, Bacteroidetes, *Enterobacteriaceae*, lactobacilli, bifidobacteria and *Akkermansia muciniphila* in the distal colon during the course of the control period (C_I-II), the clindamycin treatment period (CLI), the CDI stabilization period (CDI_I-III), the vancomycin treatment period (VNC) and the post-intervention period (PI_I-III) of the PathoGut™ SHIME experiment for six of the experimental arms, including arm 1 (CTRL), arm 2 (AB), arm 5 (LAC5V0), arm 6 (LAC10V0), arm 7 (LAC5V1) and arm 8 (LAC10V1). Dots represent mean ± SEM of technical replicas of qPCR of one time point per period.

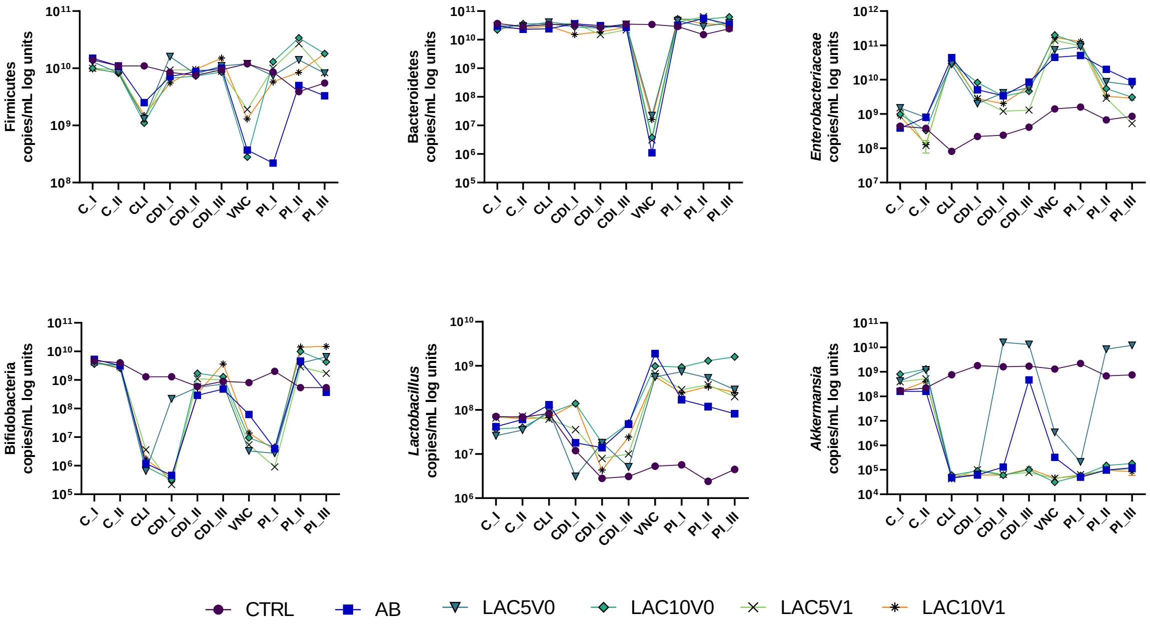

Supplement: Supplementary file 1 [file antibiotics-11-01464-s001.zip › antibiotics-1969207-supplementary.pdf]
